# Diphosphine Bioconjugates via Pt(0)-Catalyzed Hydrophosphination. A Versatile Chelator Platform for Technetium-99m and Rhenium-188 Radiolabeling of Biomolecules

**DOI:** 10.1021/acs.inorgchem.2c04008

**Published:** 2023-01-31

**Authors:** Rachel E. Nuttall, Truc Thuy Pham, Ailis C. Chadwick, Ingebjørg N. Hungnes, George Firth, Martin A. Heckenast, Hazel A. Sparkes, M. Carmen Galan, Michelle T. Ma, Paul G. Pringle

**Affiliations:** † School of Chemistry, University of Bristol, Cantock’s Close, Bristol, BS8 1TS, United Kingdom; ‡ School of Biomedical Engineering and Imaging Sciences, King’s College London, 4th Floor Lambeth Wing, St Thomas’ Hospital, London, SE1 7EH, United Kingdom

## Abstract

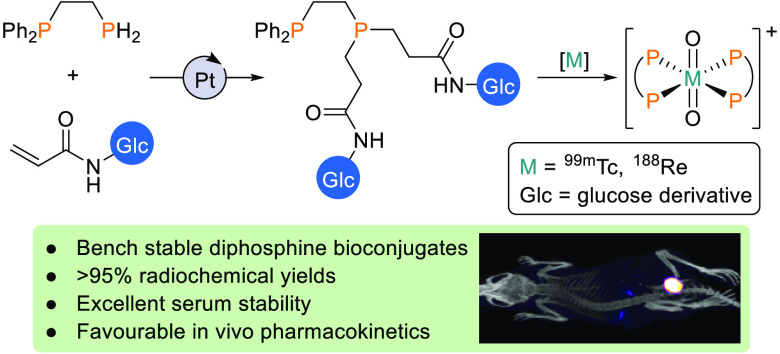

The ability to append targeting biomolecules to chelators that efficiently coordinate to the diagnostic imaging radionuclide, ^99m^Tc, and the therapeutic radionuclide, ^188^Re, can potentially enable receptor-targeted “theranostic” treatment of disease. Here we show that Pt(0)-catalyzed hydrophosphination reactions are well-suited to the derivatization of diphosphines with biomolecular moieties enabling the efficient synthesis of ligands of the type Ph_2_PCH_2_CH_2_P(CH_2_CH_2_–Glc)_2_ (**L**, where Glc = a glucose moiety) using the readily accessible Ph_2_PCH_2_CH_2_PH_2_ and acryl derivatives. It is shown that hydrophosphination of an acrylate derivative of a deprotected glucose can be carried out in aqueous media. Furthermore, the resulting glucose–chelator conjugates can be radiolabeled with either ^99m^Tc(V) or ^188^Re(V) in high radiochemical yields (>95%), to furnish separable mixtures of *cis*- and *trans*-[M(O)_2_**L**_2_]^+^ (M = Tc, Re). Single photon emission computed tomography (SPECT) imaging and *ex vivo* biodistribution in healthy mice show that each isomer possesses favorable pharmacokinetic properties, with rapid clearance from blood circulation via a renal pathway. Both *cis*-[^99m^Tc(O)_2_**L**_2_]^+^ and *trans*-[^99m^Tc(O)_2_**L**_2_]^+^ exhibit high stability in serum. This new class of functionalized diphosphine chelators has the potential to provide access to receptor-targeted dual diagnostic/therapeutic pairs of radiopharmaceutical agents, for molecular ^99m^Tc SPECT imaging and ^188^Re systemic radiotherapy.

## Abstract 2

Pt(0)−catalyzed hydrophosphination reactions are well−suited to the derivatization of diphosphines with glucose moieties. Ligands of the type Ph_2_PCH_2_CH_2_P(CH_2_CH_2_−Glc)_2_ (where Glc = a glucose moiety) have been prepared from the readily accessible Ph_2_PCH_2_CH_2_PH_2_ and acrylamide derivatives of glucosamine. The diphos-glucose conjugates are efficiently radiolabeled with ^99m^Tc and ^188^Re. This work shows that hydrophosphination of acrylated biomolecules could provide a versatile route to the bioconjugation of diphos ligands.

## Introduction

**Scheme 1:**
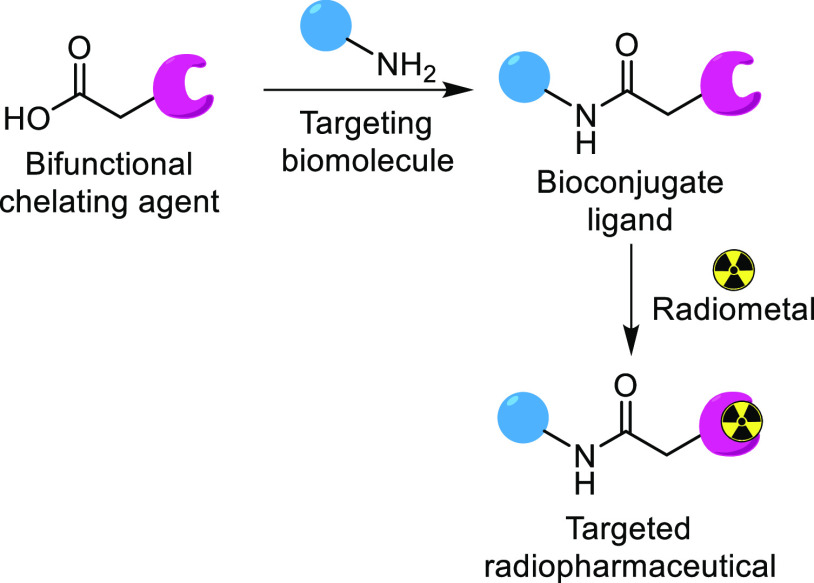
General Strategy for Targeted Radiopharmaceuticals, with Amide Coupling Exemplifying a Method of Bioconjugation

The radioactive, γ-emitting isotope, technetium-99m (^99m^Tc, *t*_1/2_ = 6 h, 90% γ, 140 keV), is widely and routinely used in radiotracers for clinical diagnostic SPECT (Single Photon Emission Computed Tomography) or γ-scintigraphy imaging of disease. Currently, ^99m^Tc imaging uses radiotracers that measure perfusion physiological processes. These ^99m^Tc-labeled radiopharmaceuticals are based on relatively simple, low molecular weight Tc complexes, and their physicochemical properties determine the biodistribution and disease-targeting capabilities.^1^ By contrast, radiopharmaceuticals that have more recently entered routine clinical use target receptors that are overexpressed in diseased tissue. These radiopharmaceuticals include PET (Positron Emission Tomography) diagnostic imaging agents, as well as therapeutic systemic agents that emit cytotoxic β^–^ or α particles. Pairs of diagnostic imaging and therapeutic radiotracers that target the same receptor are often considered “companion” or “theranostic” agents. Many of these compounds are based on radiometallic ions that are coordinated by a chelator, which in turn is attached to a biomolecule that targets receptors of diseased tissue (Scheme 1).^2^ For example, paired ^68^Ga-labeled peptides for diagnostic PET imaging and ^177^Lu-labeled peptides for systemic therapeutic agents have improved treatment outcomes for neuroendocrine and prostate cancer patients.^3−5^ New chelator platforms that enable attachment of a chelator to a biomolecule, and form stable complexes of ^99m^Tc, have potential utility for developing novel ^99m^Tc radiopharmaceuticals for use in receptor-targeted SPECT/γ-scintigraphy imaging of disease.

The chemistry of Re and Tc are closely similar, and, as Tc has no stable isotopes, the isostructural, naturally occurring rhenium (^nat^Re) compounds are often used to aid chemical characterization of new ^99m^Tc-labeled tracers.^6^ Significantly, there are two β^–^-emitting radioisotopes of Re that have suitable decay properties for systemic radiotherapy: ^186^Re (β^–^, 1.07 MeV, 92.5%; γ, 137 and 123 keV, 7.5%; *t*_1/2_ = 90 h) and ^188^Re (β^–^, 2.1 MeV, 71%; γ, 155 keV, 15%; *t*_1/2_ = 17 h). Pairs of isostructural ^99m^Tc and ^186/188^Re complexes have potential applications as dual diagnostic/therapeutic radiopharmaceuticals.^7^ Additionally, both ^99m^Tc and ^188^Re are available from benchtop generators, allowing economically viable and reliable access to these isotopes—essential criteria for many clinical applications.

**Chart 1:**
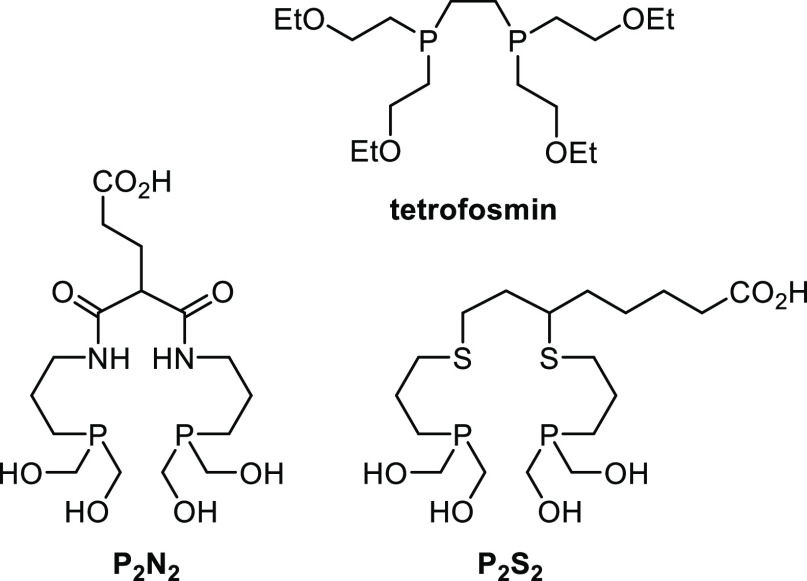
Literature Examples of Phosphine-Containing Chelators for [M(O)_2_]^+^ core (M = ^99m^Tc or ^188^Re)^8−11^

Diphosphine chelators have actual and potential utility in ^99m^Tc and ^188^Re radiopharmaceuticals. The radiopharmaceutical [^99m^Tc(O)_2_(tetrofosmin)_2_]^+^, known as *Myoview*, is used routinely for imaging cardiac perfusion; it is a ^99m^Tc(V) complex containing two **tetrofosmin** chelates (Chart 1).^8^ Tetradentate **P**_**2**_**N**_**2**_ and **P**_**2**_**S**_**2**_ ligands (Chart 1) have also previously been synthesized and radiolabeled with [^99m^Tc(O)_2_]^+^ and [^188^Re(O)_2_]^+^ cores in high radiochemical yield, using simple radiosynthetic protocols suitable for clinical application.^9−11^ In addition, the **P**_**2**_**N**_**2**_ and **P**_**2**_**S**_**2**_ ligands have been conjugated with peptides via amidation of the carboxylic acid groups.^9−11^

Orthogonal azide–alkyne chemistry is frequently employed to attach chelators to biological vectors that target receptors of diseased tissue.^12−15^ However, this “click” approach is problematic for tertiary phosphines since they generally react rapidly with azides to form iminophosphines, R′N=PR_3_ (i.e., the Staudinger Reaction) which eliminates the P-binding function. The two following strategies have been previously employed to circumvent this Staudinger problem for click chemistry with phosphines (designed for applications as ligands in homogeneous catalysis), but neither is appropriate for bioconjugation. (1) The phosphine is introduced into an existing triazole group, but reactive chlorophosphine and organolithium reagents are required,^16^ which are incompatible with the presence of many O- and N- functional groups without extensive protection/deprotection protocols. (2) The phosphine is protected by peroxide oxidation to O=PR_3_ prior to undertaking a click reaction, and then after the click reaction is completed, hydridic reduction is carried out to regenerate the PR_3_ donor,^17^ but again, these manipulations involve reagents that are generally incompatible with functional groups in biomolecules.

**Scheme 2:**
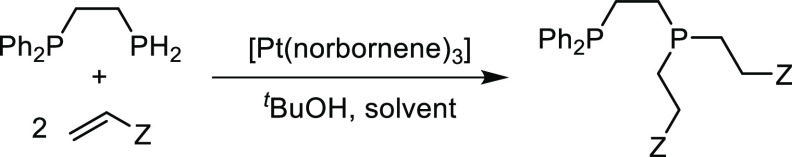
Synthesis of Functionalized Diphosphines via Pt(0)-Catalyzed Hydrophosphination of CH_2_=CHZ, (Z = CO_2_R, CONR_2_, CN) by a Tertiary-Primary Diphosphine The protic additive *t*-BuOH inhibits the formation of telomeric byproducts.^37^

The addition of a H–P^III^ bond to an unsaturated C–C bond (hydrophosphination) is an atom-efficient, versatile, and generally irreversible way of making phosphine ligands.^18−29^ Platinum(0)-catalyzed hydrophosphination is a reliable route to phosphines that contain reactive functional groups suitable for further elaboration.^30−36^ We have recently demonstrated that the air-stable diphosphine Ph_2_PCH_2_CH_2_PR_2_ (where R = CH_2_CH_2_CO_2_Me) (**L1**) is readily prepared from Ph_2_PCH_2_CH_2_PH_2_ and methyl acrylate via the Pt(0)-catalyzed hydrophosphination reaction shown in Scheme 2.^30^ Many acryl derivatives are readily accessible synthetically, potentially paving the way for hydrophosphination to provide a simple route to diphosphines with appended biologically targeting motifs.

Carbohydrates and their glycoconjugates mediate a wide range of biological processes and therefore can provide highly specific glycan markers of diseased cells that can be exploited for early diagnosis and in drug development. Although carbohydrates are the most diverse and one of the most important classes of biomolecules in nature, there are relatively few carbohydrate-based drugs in clinical use.^38^ Glucose derivatives have been widely used to target glucose-avid diseased tissue, such as cancer, or to improve the solubility, biological stability, or other pharmacological properties of drugs.^39^ The radioactive ^18^F-labeled glucose derivative, [^18^F]-FDG ([^18^F]-2-fluoro-2-deoxyglucose), is taken up by the GLUT1 transporter which is overexpressed in cancer, and [^18^F]-FDG is routinely used as a diagnostic PET imaging agent in oncology.^40,41^ We have selected glucose derivatives as model biomolecules to assess the feasibility of bioconjugation using Pt(0)-catalyzed hydrophosphination. One attraction of glucose as the target motif is that it can be derivatized from either hydroxy-protected or unprotected precursors, allowing the versatility and scope of Pt(0)-catalyzed hydrophosphination to be gauged. Furthermore, the resulting phosphine-glucose bioconjugates are likely to be highly hydrophilic, allowing the stability of the bioconjugates and their complexes in aqueous solutions and biological media to be assessed.

Herein we report the application of Pt(0)-catalyzed hydrophosphination (Scheme 2),^30^ in the production of diphosphine glycoconjugates with potential utility as Tc/Re radiotheranostics. It is shown that diphosphine derivatives of the type Ph_2_PCH_2_CH_2_P(CH_2_CH_2_CO_2_Z)_2_ (Z = CO_2_Me, CO_2_Na), coordinate to Tc(V) and Re(V), to yield complexes of stoichiometry [M(O)_2_(diphos)_2_]^+^ (M = ^nat^Re, ^99m^Tc). We further show that diphosphine-glucose bioconjugates can be prepared from acrylamide derivatives of glucose and these new diphosphine-glucose derivatives can be labeled with ^99m^Tc and ^188^Re in near-quantitative radiochemical yields. The resulting ^99m^Tc radiotracers exhibit high stability in biological milieu and have favorable biodistribution properties, as exemplified by *in vivo* SPECT imaging of these novel ^99m^Tc-tracers in mice.

**Scheme 3:**
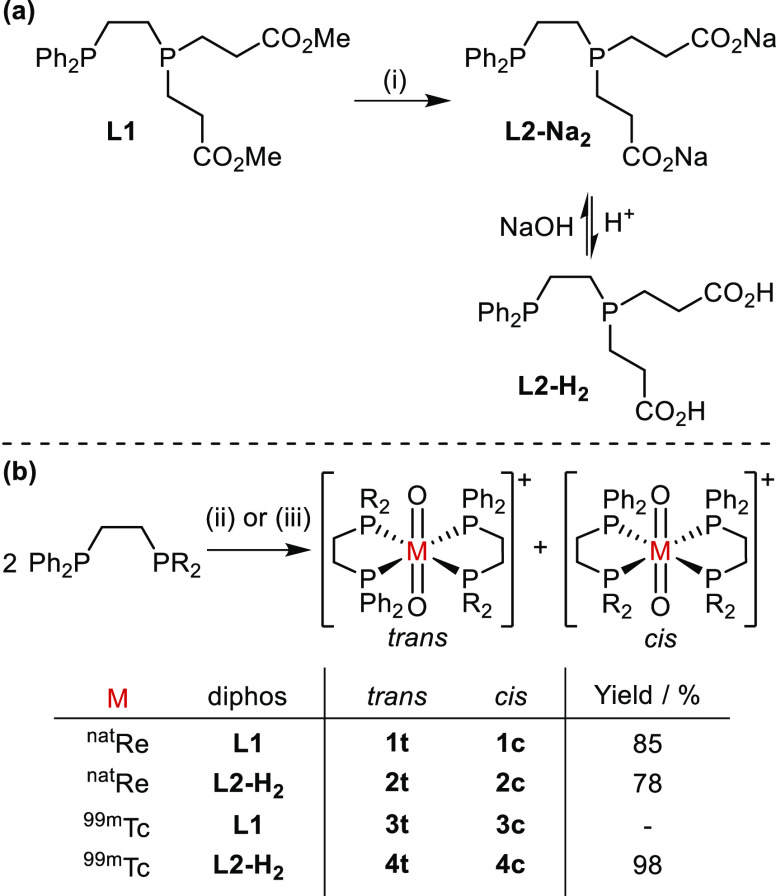
(a) Synthesis of **L2-Na**_**2**_/**L2-H**_**2**_; (b) Preparation of [M(O)_2_(**L1**)_2_]^+^ and [M(O)_2_(**L2-H**_**2**_)_2_]^+^ (M = ^99m^Tc, ^nat^Re) Conditions: (i) NaOH (2 equiv), MeOH:H_2_O (1:1), 79%; (ii) [ReI(O)_2_(PPh_3_)_2_], DCM; (iii) [^99m^TcO_4_]^−^, Sn-containing kit. Yields given for combined *cis*/*trans*-[M(O)_2_(L)_2_]^+^ and as radiochemical yields for ^99m^Tc. Note that the overall charge on complexes of **L2-H**_**2**_ is dependent on pH since this will determine the degree of deprotonation of the carboxylic acid groups.

**Scheme 4:**
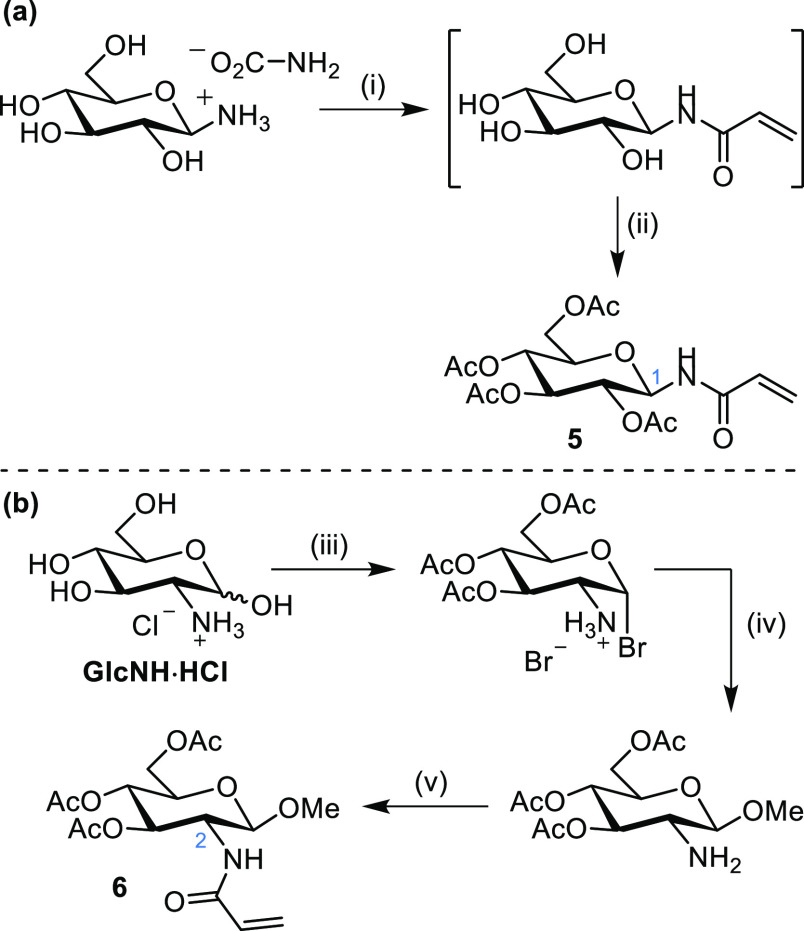
Synthesis of Glucose Substrates **5** and **6** Bearing an Activated Alkene at *C*1 and *C*2, Respectively Conditions: (i) Na_2_CO_3_ (5.7 equiv), MeOH:H_2_O (1:1) then acryloyl chloride (3.2 equiv) in THF at 0 °C; (ii) Ac_2_O, pyridine, 58% over two steps; (iii) acetyl bromide, 78%; (iv) MeOH, pyridine, 65%; (v) acryloyl chloride (1.2 equiv), NEt_3_ (1.5 equiv), DCM, 91%.

## Results

### Initial Re(V) Coordination Studies

Reaction of the previously reported hydrophosphination-derived ligand **L1** with [ReI(O)_2_(PPh_3_)_2_] yielded a mixture of geometric isomers of [Re(O)_2_(**L1**)_2_]^+^ (**1c** and **1t**) with the *trans-*Re(O)_2_ core and the unsymmetrical diphosphines coordinated *cis* or *trans* (Scheme 3).^30^ The ^31^P{^1^H} NMR spectrum showed two multiplets centered at δ_P_ 8.6 and 9.0 ppm with a coordination shift Δδ of ca. 25 ppm for both isomers (Figure S1).

**Figure 1:**
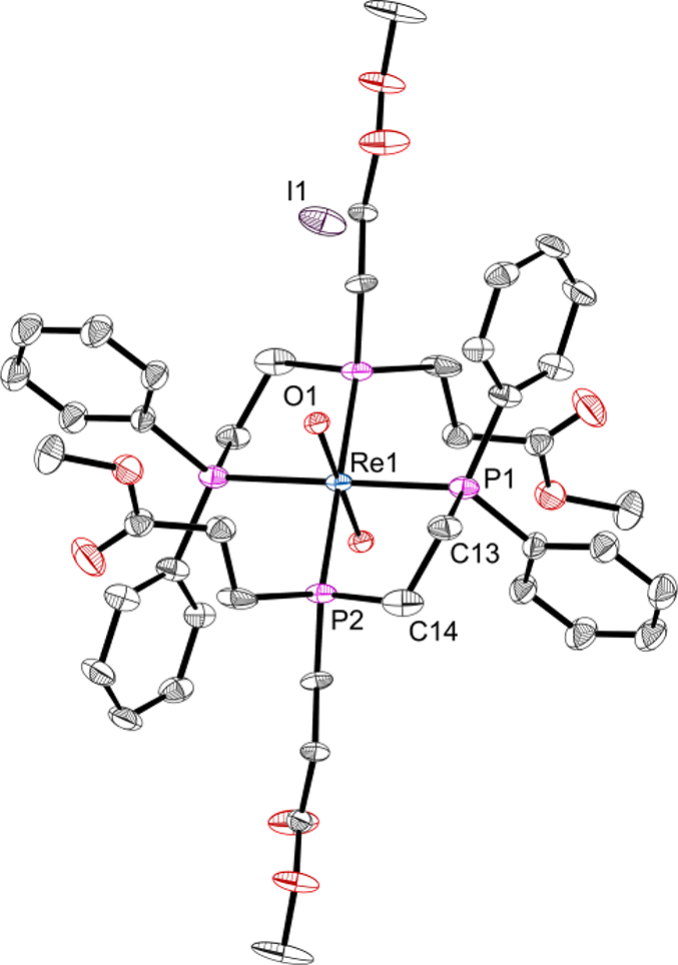
Molecular structure of *trans*-[Re(O)_2_(**L1**)_2_]I from X-ray crystallographic analysis. Hydrogen atoms are omitted for clarity. Selected bond lengths (Å) and bond angles (deg): Re(1)–P(1) 2.4651(16), Re(1)–P(2) 2.4619(17), Re(1)–O(1) 1.778(3), O(1)–Re(1)–O(1) 180, P(1)–Re(1)–P(2) 80.29(5), P(1)–Re(1)–O(1) 96.60(12), P(2)–Re(1)–O(1) 90.49(13).

Crystals of *trans*-[Re(O)_2_(**L1**)_2_]I (**1t**) suitable for X-ray crystallography were grown by slow diffusion of pentane into its methanol solution (Figure 1). The Re=O bond lengths (1.778(3) Å) and Re–P bond lengths (2.4651(16) and 2.4619(17) Å) are similar to the corresponding distances in the complex [Re(O)_2_(dmpe)_2_]PF_6_·2H_2_O.^42^

Diester **L1** was hydrolyzed with NaOH in MeOH:H_2_O (1:1) at ambient temperature and the product isolated as the moderately air-stable sodium salt **L2-Na**_**2**_ (Scheme 3(a)). Treatment of [ReI(O)_2_(PPh_3_)_2_] with the derived dicarboxylate ligand **L2-Na**_**2**_ gave cationic Re(V) complexes as a mixture of *trans* and *cis* isomers **2t** and **2c** (Scheme 3(b)). The two geometric isomers were separated by reverse-phase HPLC (using an acidic mobile phase containing trifluoroacetic acid) and fully characterized; under these HPLC conditions, *trans*-[Re(O)_2_(**L2-H**_**2**_)_2_][CF_3_CO_2_] (**2t**) eluted first.

**Figure 2:**
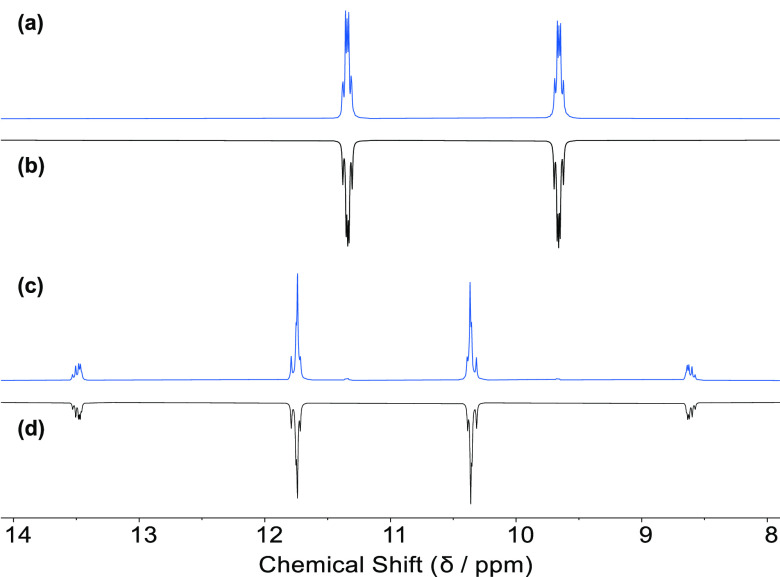
(a) Experimental and (b) simulated ^31^P{^1^H} NMR spectrum of *trans*-[Re(O)_2_(**L2-H**_**2**_)_2_]^+^ (**2t**); (c) experimental and (d) simulated ^31^P{^1^H} NMR spectrum of *cis*-[Re(O)_2_(**L2-H**_**2**_)_2_]^+^ (**2c**). See SI for the details of the simulations.

The signals observed by ^31^P{^1^H} NMR spectroscopy for **2t** and **2c**, were each simulated as AA′BB′ spin systems and the good agreement between the experimental and simulated spectra supports the assignment of the isomers (Figure 2).

### ^99m^Tc-Radiolabeling of Diester L1 and Dicarboxylate L2-Na_2_

^99m^Tc-based radiopharmaceuticals are often formulated from an instant “kit” that contains all the required nonradioactive chemicals.^43^ These kits enable routine, simple, one-step preparations of ^99m^Tc-labeled radiotracers in hospital radiopharmacies, simply by addition of generator-produced [^99m^TcO_4_]^−^ dissolved in saline solution to a kit vial, followed by incubation at ambient or elevated temperatures. The ratios of the components of the lyophilized kits produced in this work were based on *Myoview* kits and consisted of either **L1** or **L2-Na**_**2**_ diphosphine ligand, stannous chloride as reducing agent, sodium bicarbonate as buffer, and either sodium tartrate or sodium d-gluconate which coordinates to reduced ^99m^Tc intermediates, to prevent their hydrolysis and formation of insoluble ^99m^Tc-containing suspensions (see Table S1).

In an initial ^99m^Tc-radiolabeling experiment, a solution of aqueous [^99m^TcO_4_]^−^ was added to a lyophilized kit containing **L1**, and the mixture was heated at 60 °C for 30 min (Scheme 3(b)). Reverse-phase HPLC analysis indicated that numerous ^99m^Tc-labeled species had formed in >90% radiochemical yield (see Figure S2); the complexity of the product mixture is attributed to the formation of hydrolyzed ester products as *cis* and *trans* isomers.

To obviate the difficulties of analyzing the mixture of reaction products formed from diester **L1**, ^99m^Tc-radiolabeling studies were instead undertaken with dicarboxylate chelator, **L2-Na**_**2**_. Aqueous [^99m^TcO_4_]^−^ was added to the kit containing **L2-Na**_**2**_, and the reaction mixture set aside at ambient temperature for 30 min. HPLC analysis revealed the formation of two major species, with retention times of 10 and 13 min, formed in 39% and 59% radiochemical yields, respectively (see Figure S3); these products are assigned to *trans*-[^99m^Tc(O)_2_(**L2**-**H**_**2**_)_2_]^+^ (**3t**) and *cis*-[^99m^Tc(O)_2_(**L2**-**H**_**2**_)_2_]^+^ (**3c**). A low intensity radioactive signal at 2 min (ca. 2% of detected ^99m^Tc) is attributed to ^99m^Tc intermediates or unreacted [^99m^TcO_4_]^−^. The ^99m^Tc radiolabeling experiment was repeated and spiked with [^99g^TcO_4_]^−^ (^99g^Tc, *t*_1/2_ = 2 × 10^5^ years) so that Tc complexes could be analyzed further. The ^99g^Tc/^99m^Tc-labeled products were collected and analyzed by negative ion MALDI MS and signals corresponding to [M–2H]^−^ ([C_40_H_48_O_10_P_4_^99^Tc]^−^) were observed at *m*/*z* = 909.1, consistent with the assigned stoichiometry of these complexes, and indicating that the two ^99^Tc-labeled species are isomeric.

### Synthesis and ^nat^Re(V) Coordination Chemistry of Diphosphine Glycoconjugates

Having established that simple, unsymmetrical diphosphines coordinate to ^99m^Tc(V), we aimed to prepare more complex diphosphine bioconjugates, and selected two glucose derivatives to demonstrate the feasibility of applying a Pt(0)-catalyzed hydrophosphination reaction to derivatize diphosphines with biologically relevant molecules.

The glucose substrates **5** and **6** bearing an activated alkene at *C*1 and *C*2, respectively, were synthesized as shown in Scheme 4. Initially, **5** was synthesized via an azide derivative, as described in the SI, while the larger scale synthesis was achieved in two steps in 58% yield from acrylation and then acetylation of β-d-glucopyranosylammonium carbamate. Substrate **6** was synthesized in 3 steps and 46% overall yield from glucosamine hydrochloride (GlcNH·HCl) by installation of the β-OMe followed by acrylation.

**Scheme 5:**
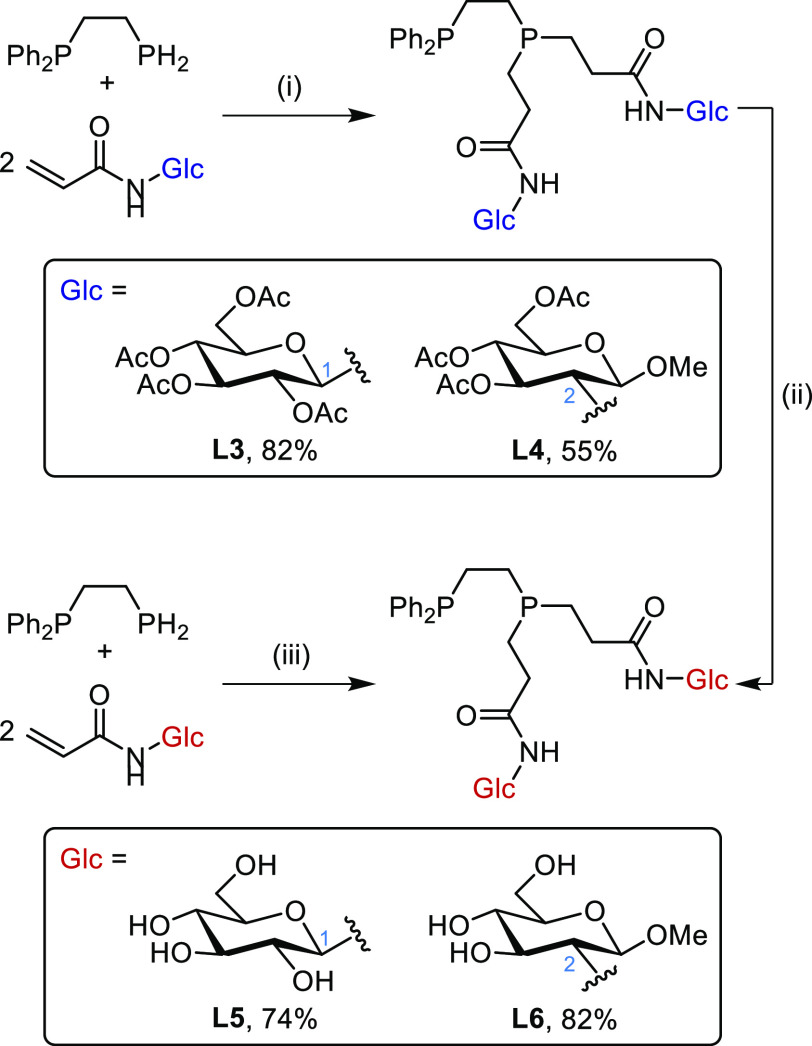
Synthesis of Diphosphine Glycoconjugates via Pt(0)-Catalyzed Hydrophosphination Conditions: (i) 2.5% or 5.0% [Pt(nbe)_3_], *i*-PrOH (20 equiv); (ii) NaOMe (0.2 mol %) in MeOH; (iii) 5.0% [Pt(nbe)_3_] in 20% aqueous *i*-PrOH gave **L6** in 80% yield (determined by ^31^P{^1^H} NMR spectroscopy).

The *C*1-conjugate **5** was subjected to the Pt(0)-catalyzed hydrophosphination conditions, using 2.5 mol % [Pt(nbe)_3_] (nbe = norbornene) and *i*-PrOH (20 equiv) as the protic additive, to give diphosphine glycoconjugate **L3** in 82% isolated yield (Scheme 5). It was found that *i-*PrOH was also a suitable protic additive in these reactions, and more convenient than *t*-BuOH which is a semisolid at room temperature. Hydrophosphination of the *C*2-conjugate **6** gave an unknown byproduct which was characterized by two doublet signals at δ_P_ +38.6 and −12.9 ppm (^3^*J*_P,P_ = 44.6 Hz) in the ^31^P{^1^H} NMR spectrum of the crude material (Figure S4). Increasing the catalyst loading to 5.0 mol % reduced the amount of byproduct and pure **L4** was isolated in 55% yield. Investigations into the identity of the unknown byproduct are ongoing. Acetate deprotection of the glucose motifs in both diphosphine glycoconjugates, **L3** and **L4**, using NaOMe gave ligands **L5** (74%) and **L6** (82%), respectively.

The Pt(0)-catalyzed hydrophosphination of activated alkenes is a reliable reaction, and we have now found that this reaction takes place even in aqueous media. Thus, **L6** was made by Pt(0)-catalyzed hydrophosphination of the unprotected glucose acylamide precursor in 20% aqueous isopropanol (Scheme 5). Significantly, this aqueous chemistry opens up the possibility of using highly hydrophilic activated alkenes as substrates for hydrophosphination.

**Scheme 6:**
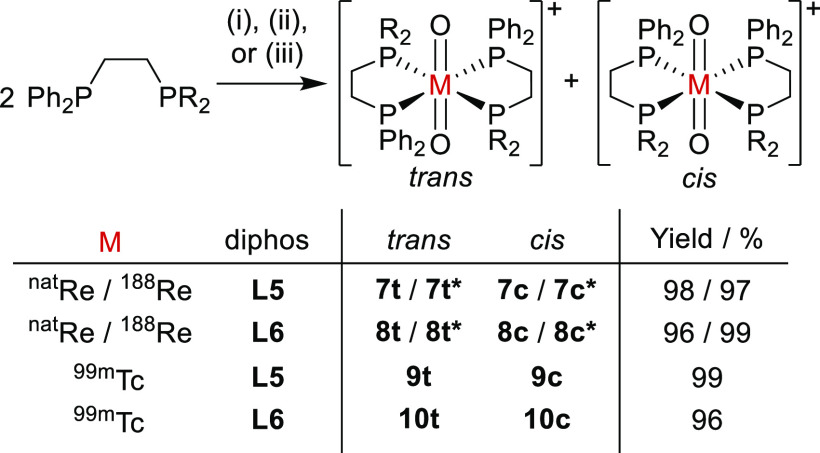
Coordination and Radiolabeling of **L5** and **L6** to Form [M(O)_2_(L)_2_]^+^, where M = ^nat^Re, ^188^Re, and ^99m^Tc Conditions: (i) [ReI(O)_2_(PPh_3_)_2_], MeOH; (ii) ^99m^TcO_4_^–^, Sn-containing kit; (iii) ^188^ReO_4_^–^, SnCl_2_, sodium citrate. Yields given for combined *cis*/*trans*-[M(O)_2_(**L**)_2_]^+^ and as radiochemical yields for ^99m^Tc and ^188^Re. The asterisk in compound numbers (e.g., 7t*) denotes a ^188^Re radiolabeled isotopologue.

The diphosphine glycoconjugate ligands **L5** and **L6** were each reacted with [ReI(O)_2_(PPh_3_)_2_] (Scheme 6), furnishing *cis*- and *trans*-[Re(O)_2_(**L**)_2_]^+^ (**7c**/**7t** and **8c**/**8t**) as expected. The *cis* and *trans* isomers of these complexes are distinguishable by ^31^P{^1^H} NMR spectroscopy based on the characteristic AA′BB′ patterns for each isomer, which are very similar to those observed for the analogous **L2-H**_**2**_ complexes (**2c** and **2t**, see Figure 2). The two geometric isomers of [Re(O)_2_(**L5**)_2_]^+^ (**7c** and **7t**) were separated by reverse-phase HPLC and characterized separately (Figure S5). After 4 days in solution, isomerization of originally pure samples of **7c** and **7t** was detected by ^31^P{^1^H} NMR spectroscopy (Figures S6 and S7). After 20 days, there were no further spectroscopic changes in either of the samples indicating that equilibration was complete. At equilibrium, an approximately 1:1 mixture of **7c** and **7t** was observed indicating that the equilibrium constant *K* ≈ 1. The kinetics of the *cis*/*trans* isomerization are slow enough to suggest that the individual isomers would retain their integrity on a clinical time scale.

### ^99m^Tc and ^188^Re Radiolabeling of Diphosphine Glycoconjugates L5 and L6

**Figure 3:**
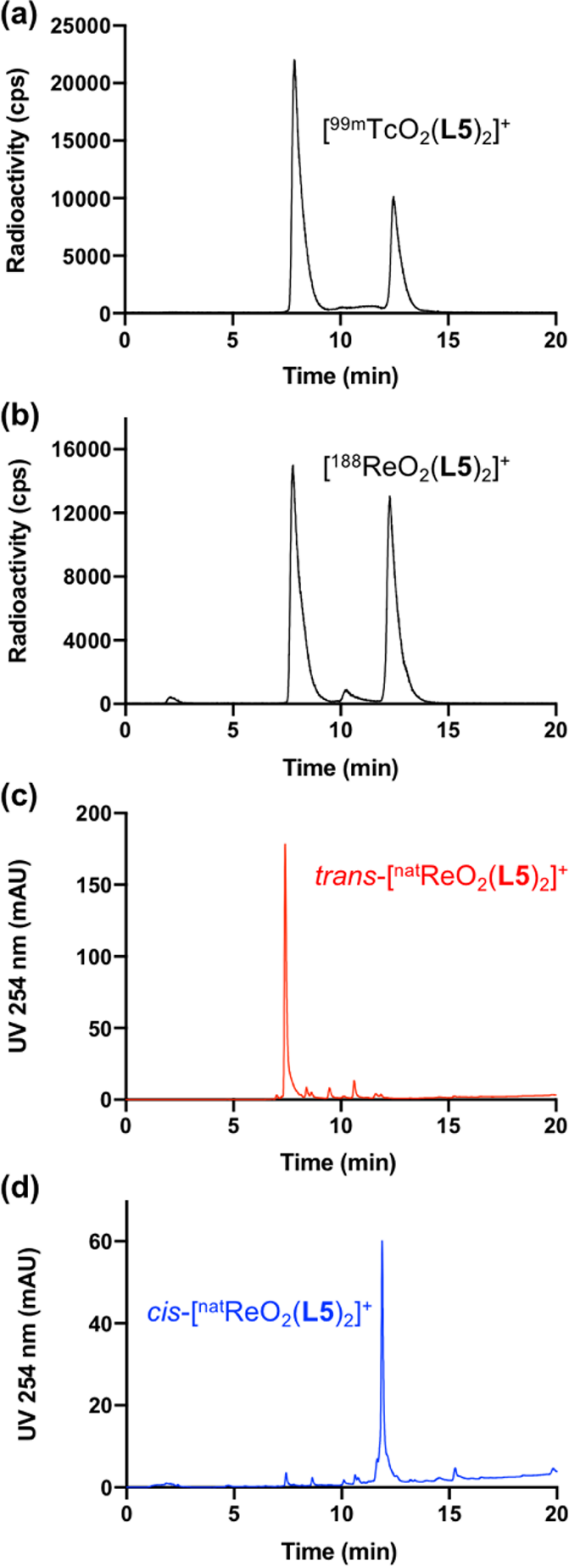
Analytical reverse-phase HPLC chromatograms of (a) [^99m^Tc(O)_2_(**L5**)_2_]^+^ (**9c** and **9t**); (b) [^188^Re(O)_2_(**L5**)_2_]^+^ (7c* and 7t*); (c) *trans*-[^188^Re(O)_2_(**L5**)_2_]^+^ (**7t**); (d) *cis*-[^188^Re(O)_2_(**L5**)_2_]^+^ (**7c**).

The new glycoconjugate **L5** was selected for more detailed radiochemical and biological evaluation, as it was thought that the fully unprotected glucose motif in **L5** would be more likely to retain glucose recognition when compared with **L6**, which is linked via *C*2 and contains a OMe group at the *C*1 position. Lyophilized mixtures of **L5**, stannous chloride, sodium gluconate, and sodium bicarbonate were prepared, providing prefabricated “kits”, similar to those prepared for ^99m^Tc-radiolabeling reactions with **L1** and **L2-Na**_**2**_ (see above). A saline solution containing generator-produced [^99m^TcO_4_]^−^ (220–280 MBq) was added to a kit, and the mixture was then left to react at ambient temperature (20–25 °C) for 5 min. Radio-HPLC analysis of this mixture revealed formation of *trans*-[^99m^Tc(O)_2_(**L5**)_2_]^+^ (**9t**) (7.88 min) and *cis*-[^99m^Tc(O)_2_(**L5**)_2_]^+^ (**9c**) (12.51 min) in exceptionally high radiochemical yields of 70% and 29%, respectively, with <1% of ^99m^Tc present as unreacted [^99m^TcO_4_]^−^ or other ^99m^Tc species (Figure 3a).

The radionuclide, ^188^Re, is available from a ^188^W/^188^Re generator. Radioactive decay of parent ^188^W (*t*_1/2_ = 69 days) to ^188^Re enables a continuous source of ^188^Re for 6–24 months (depending on user requirements). ^188^Re is eluted from the generator by saline solution, in the form of [^188^ReO_4_]^−^. To assess the ability of diphosphines to complex medically useful radioisotopes of Re, **L5** was radiolabeled with ^188^Re. First, [^188^ReO_4_]^−^ was reduced to a Re(V)-citrate precursor, by heating a saline solution of [^188^ReO_4_]^−^ with stannous chloride at 90 °C in the presence of sodium citrate.^9,11^ Diphosphine **L5** was then added to a solution containing the ^188^Re(V)-citrate precursor, and the mixture heated at 90 °C for 30 min to yield *trans*-[^188^Re(O)_2_(**L5**)_2_]^+^ (7t*) (7.80 min) and *cis*-[^188^Re(O)_2_(**L5**)_2_]^+^ (7c*) (12.31 min) in 52% and 45% radiochemical yield, respectively (Figure 3b). The very similar retention times of ^99m^Tc and ^188^Re analogues are consistent with these complexes being isostructural. A third, unidentified species (10.57 min) was formed in 2% radiochemical yield.

The ^188^Re radiolabeling could also be accomplished at ambient temperature, with a reaction time of only 5 min; under these conditions, lower radiochemical yields of 45% for 7t* and 40% for 7c* were obtained (Figure S8). At ambient temperature, unreacted [^188^ReO_4_]^−^/^188^Re(V)-citrate precursor accounted for 7% of ^188^Re radioactive species and an unidentified species was also formed in 8% radiochemical yield.

To confirm the identity of these radiolabeled products, the analogous, naturally occurring Re complexes were also analyzed by HPLC: **7t** eluted at 7.41 min and **7c** eluted at 11.89 min (Figure 3c–d). These ^nat^Re species exhibit similar chromatographic properties to the analogous radioactive ^99m^Tc and ^188^Re compounds. The small difference in retention times between ^nat^Re and ^188^Re isotopologues is an artifact of the configuration of the UV and radioactivity (scintillation) detectors in series.

The diphosphine-glucose conjugate, **L6**, was also radiolabeled with ^99m^Tc and ^188^Re, using the same radiosynthetic procedures. Diphosphine **L6** was similarly incorporated into a lyophilized kit mixture for radiolabeling with ^99m^Tc to give [^99m^Tc(O)_2_(**L6**)_2_]^+^ (**10c** and **10t**); **L6** was added to solutions of ^188^Re(V)-citrate for radiolabeling to give [^188^Re(O)_2_(**L6**)_2_]^+^ (8c* and 8t*). Similar to **L5**, the putative *cis* and *trans* isomers were formed in high radiochemical yields (>96% for ^99m^Tc isomers, >99% for ^188^Re isomers; Figure S9).

### Stability of *cis*- and *trans*-[^99m^Tc(O)_2_(L5)_2_]^+^ (9c and 9t) in Serum

The stabilities of the new radiotracers, **9c** and **9t**, in serum were evaluated. First, each isomer was isolated from the radiolabeling reaction mixture which included their separation from unreacted **L5**. Then **9c** and **9t** were separately incubated in human serum. Radio-HPLC analysis indicated that both isomers were very stable in serum: for **9c**, 99.7% remained intact after 2 h, decreasing to 95.0% after 24 h; for **9t**, 98.6% of the complex remained intact after 2 h, decreasing to 93.4% after 24 h (Figure S10).

### SPECT/CT and Biodistribution of *cis*- and *trans*-[^99m^Tc(O)_2_(L5)_2_]^+^ (9c and 9t) in Healthy Mice

**Figure 4:**
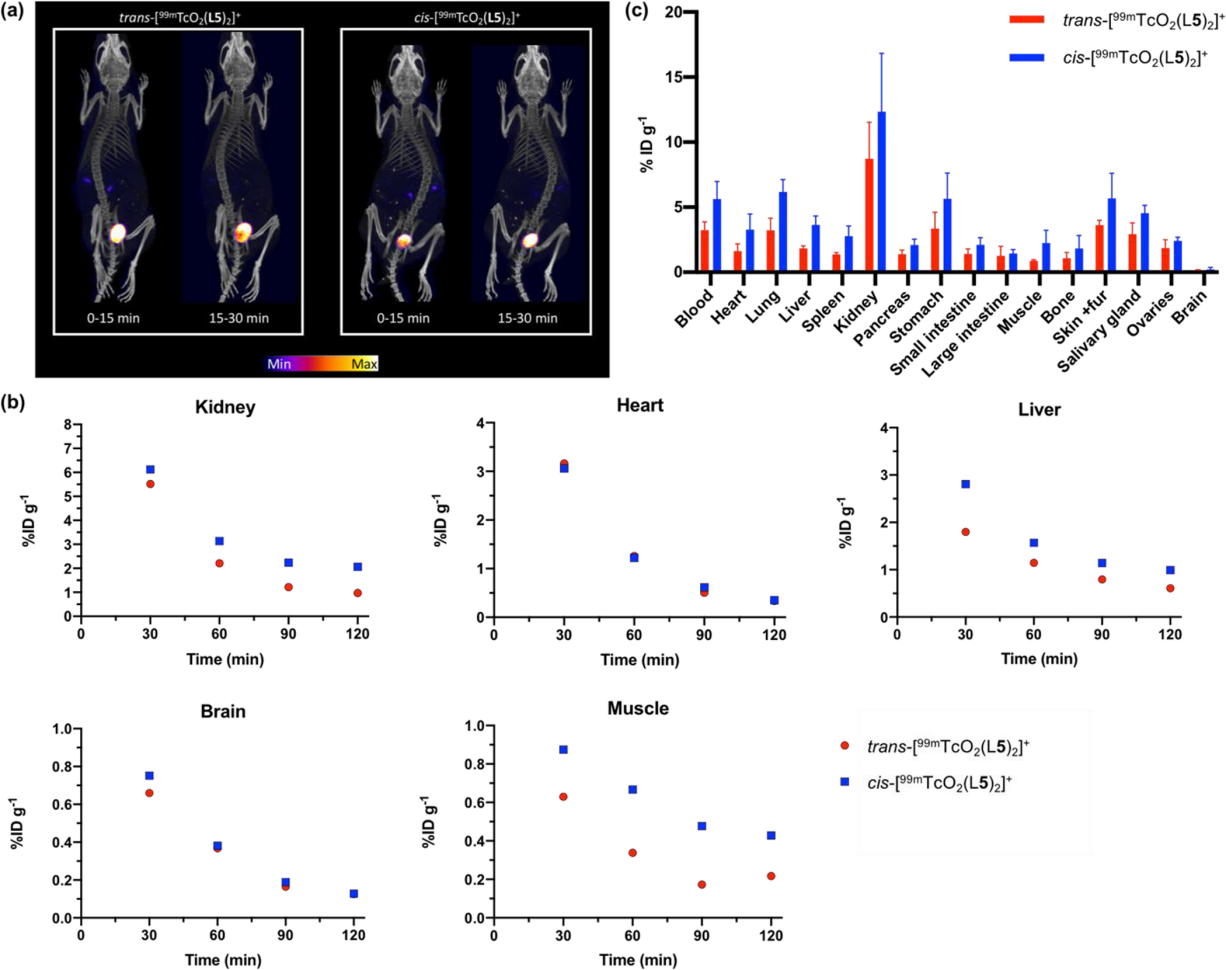
(a) SPECT/CT maximum intensity projections of healthy Balb/c mice administered and **9c** and **9t**; (b) SPECT image analysis of healthy Balb/c mice administered **9c** and **9t**. Regions of interest were selected on VivoQuant (inviCRO, LLC, Boston, USA), and percentage injected dose per milliliter (% ID/mL) were calculated for each of **9c** (*n* = 1) and **9t** (*n* = 1). (c) Biodistribution (30 min postinjection) of mice, administered either **9c** or **9t** intravenously (*n* = 4 per group).

SPECT/CT images were acquired using **9c** and **9t** (Figure 4a). Each isomer was separately injected intravenously to healthy Balb/c female mice (fasted for 12–14 h prior to tracer administration), followed by SPECT scanning for 2 h. Both **9c** and **9t** cleared circulation rapidly, predominantly via a renal pathway. For mice that had been administered **9c**, 60% of ^99m^Tc activity was associated with the bladder/urine 30 min postinjection. Similarly, for mice that had been administered **9t**, image quantification showed that by 30 min postinjection, 60% of ^99m^Tc activity was associated with the bladder/urine. In other measured organs and tissue (including liver, muscle, kidneys, heart/blood pool, and brain) the concentration of ^99m^Tc activity for both isomers decreased from 30 min postinjection to 2 h postinjection (Figure 4b).

Biodistribution studies were also undertaken, in which healthy Balb/c female mice were administered radiotracer (Figure 4c). Mice were culled 30 min postinjection, and their organs harvested for *ex vivo* weighing and tissue counting for radioactivity. For measured organs, the highest ^99m^Tc radioactivity concentration was observed in the kidneys, with 12.3 ± 4.5%ID g^–1^ for **9c**, and 8.7 ± 2.8%ID g^–1^ for **9t**, consistent with SPECT images showing high renal excretion. Relatively low levels of ^99m^Tc radioactivity concentration (<7%ID g^–1^) were observed for all other measured organs. Notably, for many measured organs, average ^99m^Tc radioactivity concentration was higher for animals administered **9c**, compared with animals administered **9t**. This is possibly a result of slightly higher blood retention of **9c**: blood activity measured 5.6 ± 1.3%ID g^–1^ for **9c**, and 3.2 ± 0.6%ID g^–1^ for **9t**, 30 min postinjection.

**Figure 5:**
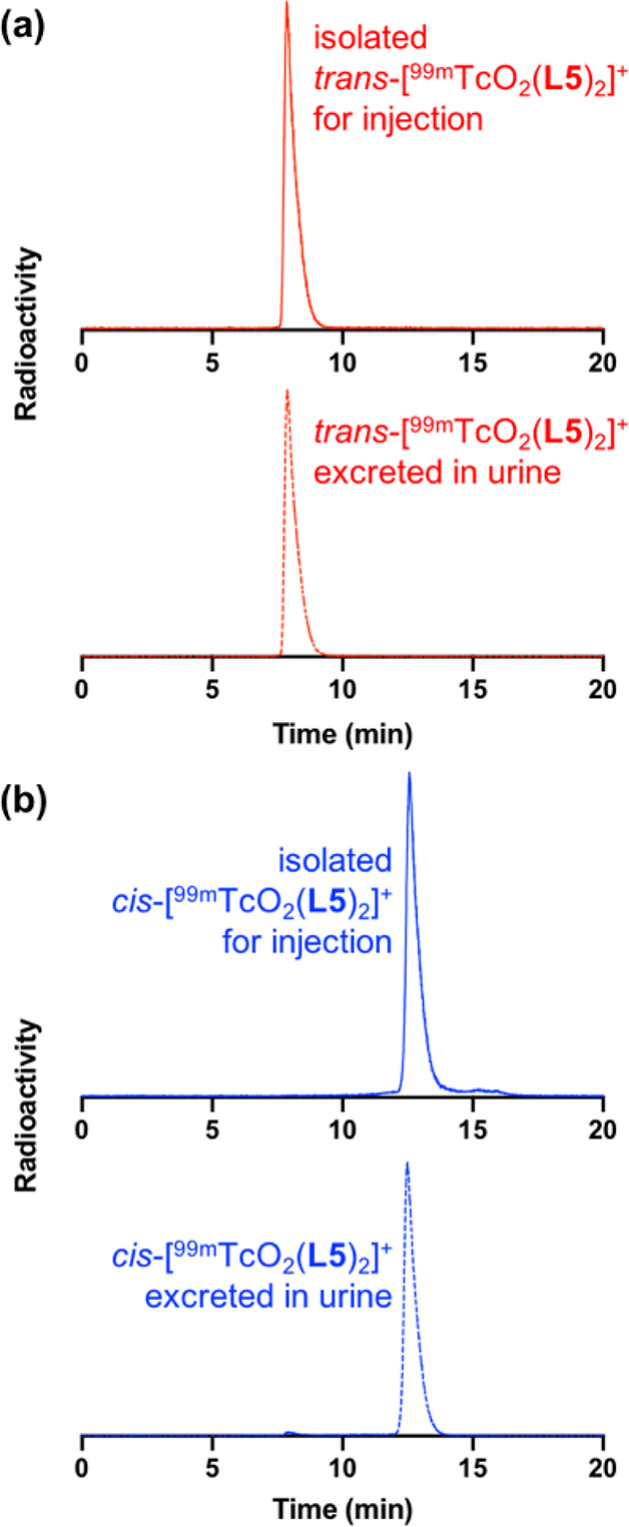
Radio-HPLC analysis of urine administered to mice intravenously administered either (a) **9t** or (b) **9c** shows that both radiotracers are excreted intact.

Urine was also collected from these mice. Radio-HPLC analysis (Figure 5) of urine showed that both **9c** and **9t** were excreted intact, consistent with the high serum stability observed for both radiotracers.

## Discussion

We have shown that Pt(0)-catalyzed hydrophosphination gives efficient access to biomolecule-functionalized diphosphines. The requisite acrylamide groups were straightforwardly introduced on two different glucose sites, namely, *C*1 or *C*2. Moreover, the ability to perform the reaction in *i*-PrOH (with 0–20% H_**2**_O), with highly polar biomolecular derivatives to form diphosphine bioconjugates such as **L6**, expands the potential substrate scope to a large range of targeted, biologically active molecules. Additionally, it negates the need for common protecting groups, which are typically used to solubilize biomolecular precursors in nonpolar organic solvents to allow for synthetic derivatization. In turn, this removes the need for a deprotection reaction, in which conditions are often incompatible with newly introduced and sensitive phosphine groups. We envisage that other classes of biomolecules, including peptides and small molecules, could also be derivatized with acryl groups, allowing many compounds of biological relevance to be functionalized with a diphosphine.

Significantly, the resulting air-stable diphosphines are capable of quantitative radiolabeling of ^99m^Tc(V) under mild reaction conditions. We undertook ^99m^Tc radiolabeling of **L2-Na**_**2**_, **L5**, and **L6** using lyophilized kits (containing phosphine ligand and reducing agent) and generator-produced [^99m^TcO_4_]^−^, demonstrating the feasibility of using this diphosphine platform for rapid, one-step, kit-based ^99m^Tc radiolabeling of receptor-targeted radiopharmaceuticals. The simple kit-based ^99m^Tc radiolabeling methods described here are inspired by, and similar to, clinical protocols used for aseptic preparation of widely and routinely used perfusion agents. Notably, these radiolabeling methods contrast with the multistep, complicated procedures used for some newer, receptor-targeted ^99m^Tc radiotracers currently being evaluated in clinical studies.^44^ The ability to prepare ^99m^Tc-labeled receptor-targeted radiotracers using a kit could increase access to receptor-targeted radiopharmaceuticals, and increase the utility and healthcare benefits of ^99m^Tc and SPECT/γ-scintigraphy infrastructure.

Additionally, these new diphosphine bioconjugates chelate both ^99m^Tc(V) and ^188^Re(V)—the latter also in high radiochemical yields (≥85%, even with only 5 min reaction at ambient temperature)—leading to the possibility of using this diphosphine bioconjugate platform for the development of a receptor-targeted, isostructural dual diagnostic/therapeutic pair (a “theranostic” agent) for molecular ^99m^Tc SPECT imaging and ^188^Re systemic radiotherapy.^7,45,46^

A further potential advantage of this platform is that multiple copies of a targeting motif can be introduced into a single molecule with relative ease. The hydrophosphination reaction here appends two copies of a targeting motif to each diphosphine; upon Tc(V) or Re(V) coordination, the number of copies increases to four per radiotracer molecule. Multimeric imaging agents, particularly those that incorporate peptides, have demonstrated increased *in vivo* accumulation at target tissue relative to their monomeric analogues, and are effective contrast agents.^47−49^ There are a few examples in which ^99m^Tc coordination by two equivalents of a chelator-peptide bioconjugate yields a radiotracer containing two copies of a targeting motif.^50−52^ Our new diphosphine platform is eminently suited to developing molecular ^99m^Tc/^188^Re radiotracers containing multiple copies of a targeting group.

The new diphosphine bioconjugates yield radiotracers consisting of *cis* and *trans* geometric isomers. This is potentially a disadvantage, as prior to clinical application of any new radiotracer based on this platform, the isomers would likely require separate assessment to determine whether or not they are biologically equivalent to each other. However, we note that the ^68^Ga-labeled prostate cancer radiotracer, ^68^Ga-HBED-PSMA, consists of at least two distinct chemical species,^53,54^ and yet despite this, the individual *in vivo* behavior of each of these species has not been assessed, and this has not prevented widespread and routine use of ^68^Ga-HBED-PSMA.^55^ Notably, the slow isomerization in solutions observed for *cis*- and *trans*-[Re(O)_2_(**L5**)_2_]^+^ (**7c** and **7t**) was not observed for ^99m^Tc analogues after 24 h in serum, nor in the excreted urine, suggesting that this isomerization appears to take place over a longer time scale than clinically relevant timeframes. Our initial *in vivo* characterization of each of *cis*-[^99m^Tc(O)_2_(**L5**)_2_]^+^ and *trans*-[^99m^Tc(O)_2_(**L5**)_2_]^+^ (**9c** and **9t**) shows that the two isomers have similar pharmacokinetic profiles to each other, with both exhibiting fast blood clearance via a renal pathway.

The development of a ^99m^Tc-labeled, GLUT1-targeted radiotracer would enable γ-scintigraphy/SPECT imaging of metabolic status, providing a SPECT-equivalent radiopharmaceutical of the widely used PET diagnostic glucose analogue, [^18^F]-FDG. There have been several previous attempts to label glucose or other carbohydrate derivatives with ^99m^Tc for imaging of glucose uptake *in vivo*.^56^ Some of these derivatives have demonstrated inhibitory activity of the hexokinase enzyme, which phosphorylates glucose in glucose metabolic pathways.^57−59^ However, only a few of these ^99m^Tc tracers demonstrate uptake in glucose-avid tissue.^59−62^ Presumably this is due to lack of molecular recognition of modified/conjugated glucose moieties by GLUT1 and other GLUT receptors, which transport glucose into cells. Furthermore, it is possible that some of the ^99m^Tc tracers that do show tumor accumulation are taken up via nonspecific mechanisms unrelated to GLUT1 expression. Although our new radiotracers contain four glucose units per molecule, SPECT/CT scanning in fasted mice showed no evidence of ^99m^Tc retention in glucose-avid organs, such as the heart or brain, both of which demonstrate significant accumulation of ^18^F in [^18^F]-FDG PET scans.^63,64^ Similar to the case of the great majority of ^99m^Tc-labeled glucose derivatives, GLUT1 and other glucose transporters do not recognize the modified glucose moieties of **L5**.

**Chart 2:**
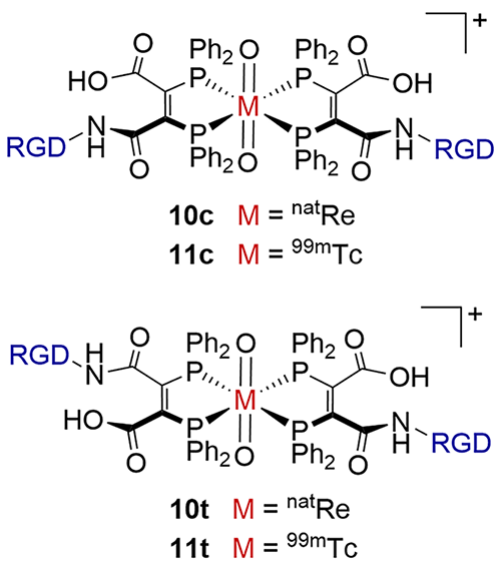
RDG-Conjugated Diphosphine Complexes

Despite this absence of uptake of [^99m^Tc(O)_2_(**L5**)_2_]^+^ (**9c** and **9t**) in highly metabolic tissue, the high stability and rapid blood clearance of both **9c** and **9t**, and the lack of accumulation of ^99m^Tc activity in healthy organs is auspicious. It suggests that other, more targeted radiotracers based on this platform will be similarly stable *in vivo* and have low off-target accumulation, and will hence provide high contrast SPECT/γ-scintigraphy images. In the case of any complementary ^188^Re derivatives, the rapid clearance of these agents from healthy tissue would minimize radiation doses to healthy organs. While the four glucose units of [^99m^Tc(O)_2_(**L5**)_2_]^+^ no doubt contribute to the favorable clearance of [^99m^Tc(O)_2_(**L5**)_2_]^+^, it is plausible that other radiotracers based on this platform (for example, derivatives containing hydrophilic receptor-targeted peptides) will possess similarly favorable biodistribution properties. In this regard, we recently reported the RGD-conjugated diphosphine complexes of ^nat^Re (**10c**/**10t**) and ^99m^Tc (**11c**/**11t**) shown in Chart 2, where RGD is a cyclic peptide that targets α_v_β_3_-integrin receptors. While complexes **10** and **11** were shown to target diseased tissue, the synthesis of the diphosphine has some limitations including the variation of the diphosphine structure. In contrast, it can readily be envisaged how RGD could be incorporated into a wide range of diphosphines by adaptation of the versatile hydrophosphination route reported here.

## Conclusions

New chelator platforms that enable simple and efficient labeling of receptor-targeted biomolecules with radiometals have utility in nuclear medicine. We have demonstrated that diphosphines functionalized with glucose units can be efficiently prepared using Pt(0)-catalyzed hydrophosphinations of acrylamides. Notably, it has also been demonstrated that the hydrophosphinations of hydrophilic, unprotected glucose derivatives can be accomplished in aqueous media. The resulting diphosphine-glucose bioconjugates can be radiolabeled with both ^99m^Tc and ^188^Re in near-quantitative radiochemical yields. SPECT imaging studies in mice provide evidence that the new ^99m^Tc radiotracers possess propitious pharmacokinetic properties, and the requisite high metabolic stability. Our new chemical technology therefore has significant potential for the future development of theranostic pairs of chemically analogous diagnostic ^99m^Tc-labeled radiotracers and radiotherapeutic ^188^Re-labeled agents.

## Supporting Information Available

Experimental procedures and characterization data, including radiolabeling procedures, selected NMR spectra, supplementary figures, and X-ray crystallographic tables (PDF)
