# Supplementary material for: Diphosphine Bioconjugates via Pt(0)-Catalyzed Hydrophosphination. A Versatile Chelator Platform for Technetium-99m and Rhenium-188 Radiolabeling of Biomolecules
Source: Inorg Chem. 2023 Jan 31;62(50):20582–92. doi: 10.1021/acs.inorgchem.2c04008 (PMC10731653; doi:10.1021/acs.inorgchem.2c04008)
Supplement: Supplementary file 1 — ic2c04008_si_001.pdf [file ic2c04008_si_001.pdf]

Supporting Information

Diphosphine bioconjugates via Pt(0)-catalysed hydrophosphination. A versatile chelator platform for technetium-99m and rhenium-188 radiolabelling of biomolecules

Rachel E. Nuttall,[a,b] Truc Thuy Pham,[b] Ailis C. Chadwick,[a] Ingebjørg N. Hungnes,[b] George Firth,[b] Martin A. Heckenast,[a] Hazel A. Sparkes,[a] M. Carmen Galan,*[a] Michelle T. Ma,*[b] Paul G. Pringle*[a]

[a] School of Chemistry, University of Bristol, Cantock's Close, Bristol, BS8 1TS, UK.

[b] School of Biomedical Engineering and Imaging Sciences, King's College London, 4th Floor Lambeth Wing, St Thomas' Hospital, London, SE1 7EH, UK.

TABLE OF CONTENTS

Table of Contents 2

1 Additional Figures 3

2 General Experimental 8

3 Experimental Procedures 10

3.1.1 Synthesis of $Ph_2PCH_2CH_2P(CH_2CH_2CO_2Me)_2$, **L1** 10

3.1.2 Synthesis of $[Re(O)_2($**L1**$)_2]I$, **1c** and **1t** 10

3.1.3 Synthesis of $Ph_2PCH_2CH_2P(CH_2CH_2CO_2Na)_2$, **L2-Na₂** 10

3.1.4 Synthesis of *cis*- and *trans*-$[Re(O)_2($**L2-H₂**$)_2]^+$, **2c** and **2t** 11

3.1.5 Synthesis of 2,3,4,6-tetra-*O*-acetyl-α-D-glucopyranosyl bromide, **S1** 12

3.1.6 Synthesis of 1-azido-1-deoxy-2,3,4,6-tetra-*O*-acetyl-β-D-glucopyranose, **S2** 12

3.1.7 Synthesis of 1-amino-1-deoxy-2,3,4,6-tetra-*O*-acetyl-β-D-glucopyranose, **S3** 12

3.1.8 Synthesis of 1-acrylamido-1-deoxy-2,3,4,6-tetra-*O*-acetyl-β-D-glucopyranose, **5** 12

3.1.9 Synthesis of protected $Ph_2PCH_2CH_2P(CH_2CH_2CONH^{C1}Glc)_2$, **L3** 13

3.1.10 Synthesis of $Ph_2PCH_2CH_2P(CH_2CH_2CONH^{C1}Glc)_2$, **L5** 14

3.1.11 Synthesis of *cis*- and *trans*-$[Re(O)_2($**L5**$)_2]^+$, **7c** and **7t** 14

3.1.12 Synthesis of 2-amino-2-deoxy-3,4,6-tri-*O*-acetyl-α-D-glucopyranosyl bromide·HBr, **S4** 15

3.1.13 Synthesis of Methyl 2-amino-2-deoxy-3,4,6-tri-*O*-acetyl-β-D-glucopyranose, **S5** 15

3.1.14 Synthesis of Methyl 2-acrylamido-2-deoxy-3,4,6-tri-*O*-acetyl-β-D-glucopyranose, **6** 15

3.1.15 Synthesis of Methyl 2-acrylamido-2-deoxy-β-D-glucopyranose, **S6** 15

3.1.16 Synthesis of protected $Ph_2PCH_2CH_2P(CH_2CH_2CONH^{C2}Glc)_2$, **L4** 16

3.1.17 Synthesis of $Ph_2PCH_2CH_2P(CH_2CH_2CONH^{C2}Glc)_2$, **L6** 16

3.1.18 Synthesis of $[Re(O)_2($**L6**$)_2]I$, **8c** and **8t** 17

4 Radiolabelling Procedures 17

4.1.1 Kit preparation: 17

4.1.2 Radiolabelling with $^{99m}TcO_4^-$: 18

4.1.3 Radiolabelling of **L5** and **L6** with $^{188}ReO_4^-$: 18

4.1.4 Serum stability of *cis* and *trans*-$[^{99m}Tc(O)_2($**L5**$)_2]^+$ (**9c** and **9t**): 19

4.1.5 *In vivo* imaging of *cis* and *trans*-$[^{99m}Tc(O)_2($**L5**$)_2]^+$ (**9c** and **9t**) in healthy mice: 19

4.1.6 Biodistribution of *cis* and *trans*-$[^{99m}Tc(O)_2($**L5**$)_2]^+$ (**9c** and **9t**) in healthy mice: 19

5 Crystallographic Details 20

6 Selected NMR Spectra 21

7 References 36

1 ADDITIONAL FIGURES

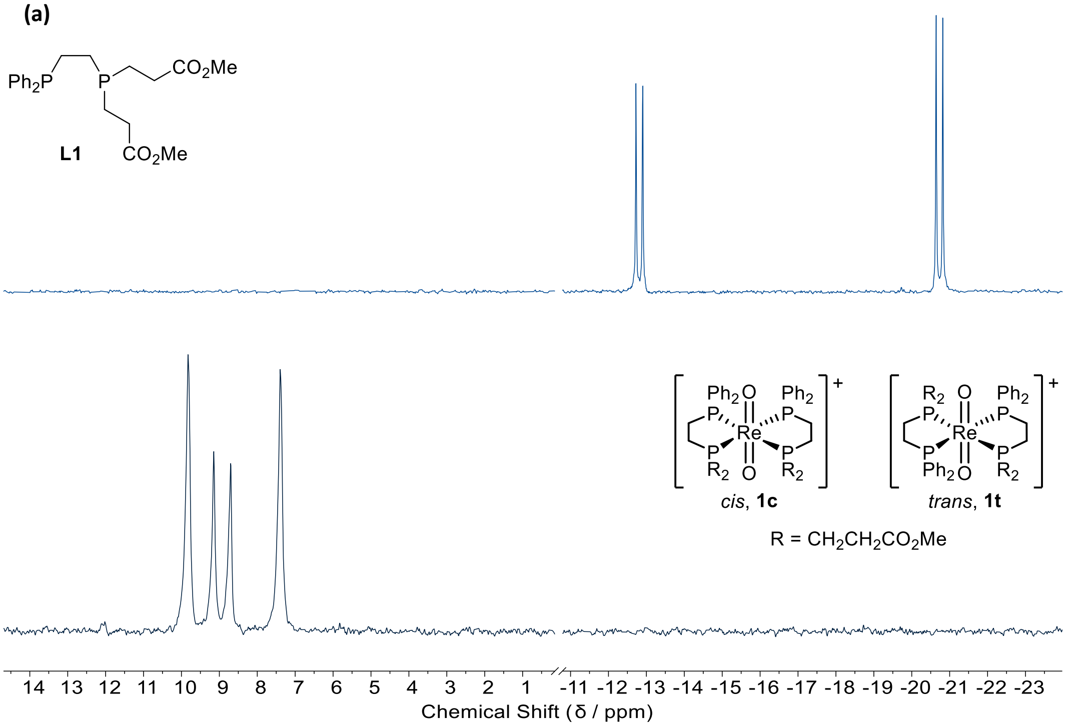

Figure S1 $^{31}P\{^1H\}$ NMR (122 MHz, CD_2Cl_2) spectra of (a) diester ligand **L1**; (b) *cis*- and *trans*-$[Re(O)_2(\mathbf{L1})_2]I$ (**1c** and **1t**).

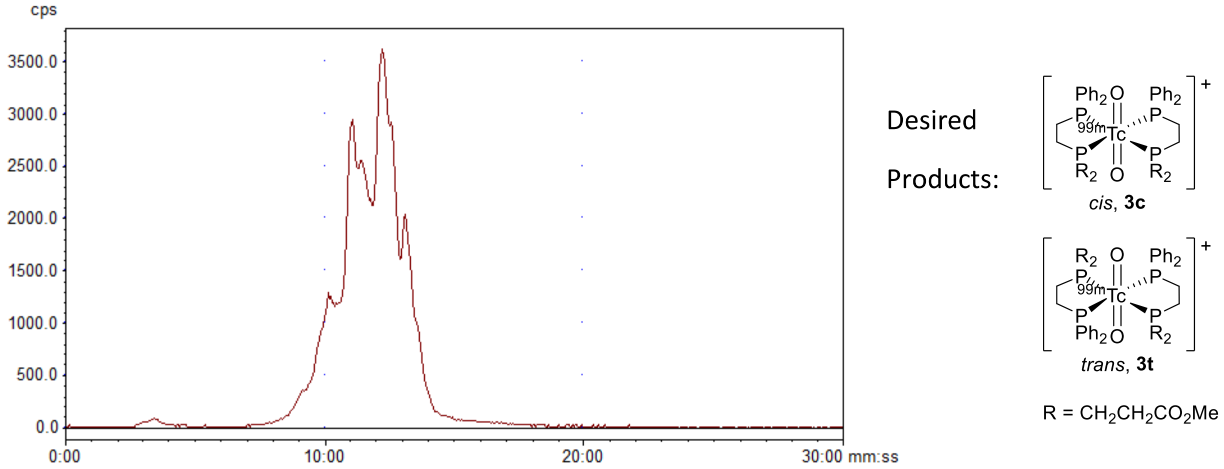

Figure S2 A reaction mixture containing **L1**, $^{99m}TcO_4^-$ and reducing agent yielded multiple radiolabelled products, as evidenced by analysis using reverse phase radio-HPLC chromatography.

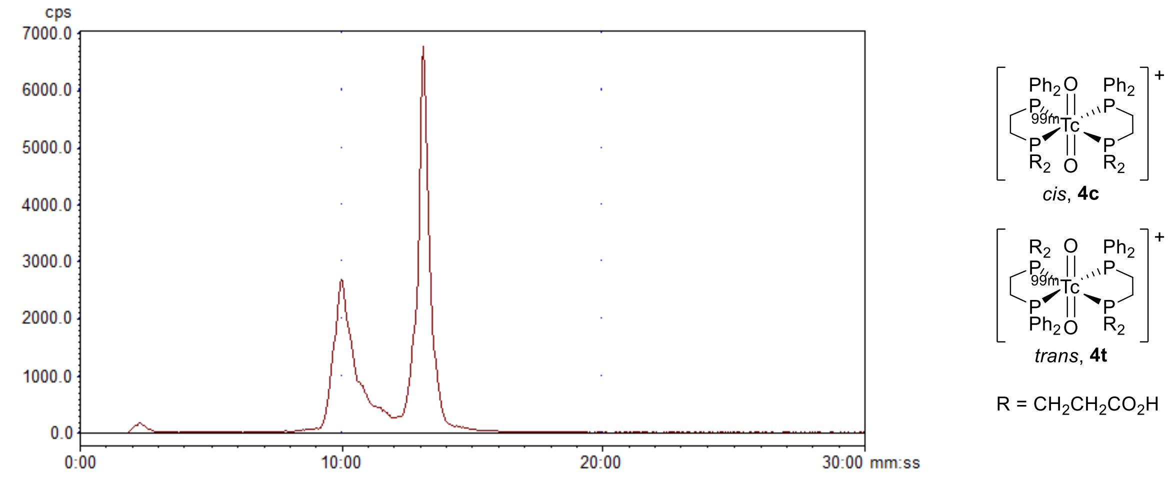

Figure S3 Radio-HPLC chromatogram showing formation of *cis* and *trans*-[^{99m}Tc(O)$_2$(**L2-H$_2$**)$_2$]$^+$ (**4c** and **4t**).

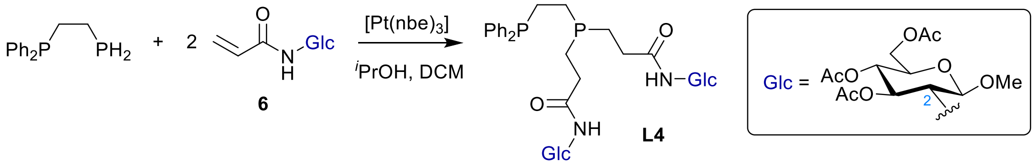

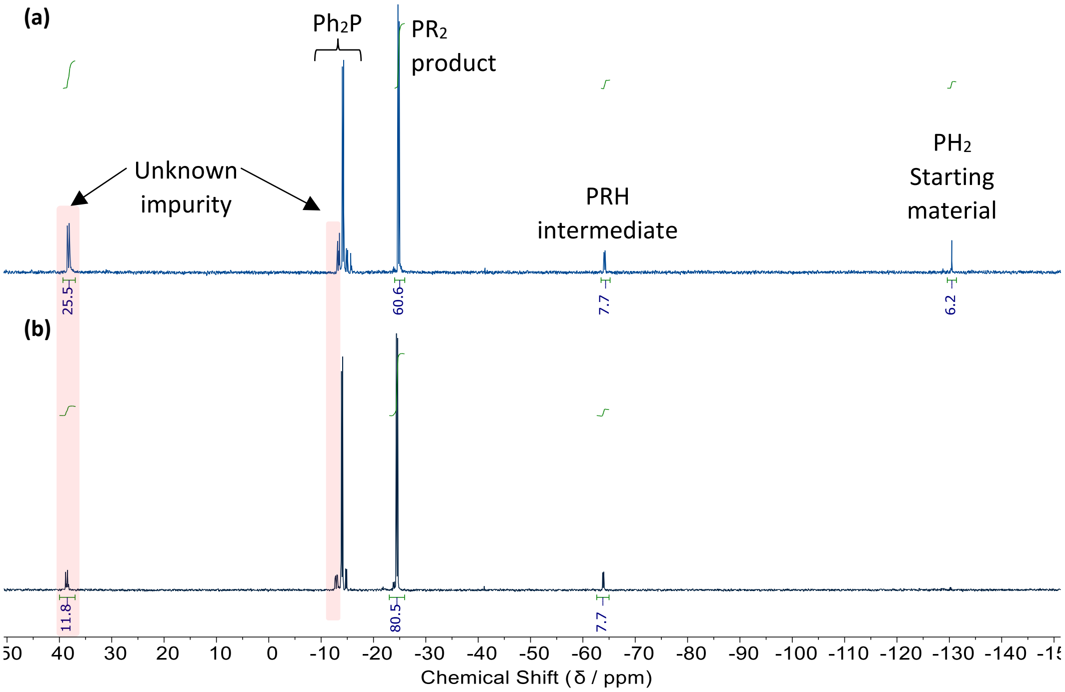

Figure S4 ^{31}P{^{1}H} NMR (122 MHz, DCM) spectra of Pt(0)-catalysed hydrophosphination of *C*2 conjugated glucose substrate **6** at (a) 2.5% and (b) 5.0% [Pt(nbe)$_3$].

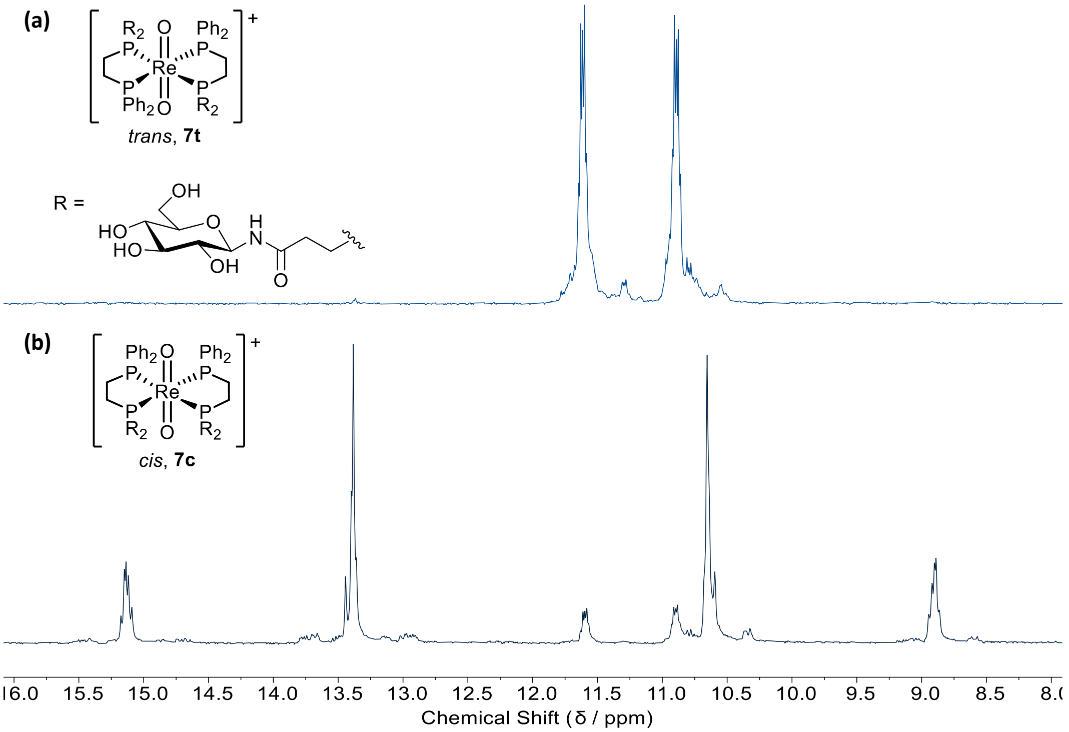

Figure S5 $^{31}P\{^{1}H\}$ NMR (202 MHz, CD_3OD) spectra of (a) *trans*- and (b) *cis*-$[Re(O)_2(\mathbf{L5})_2]^+$ (**7t** and **7c**), separated by reverse-phase HPLC.

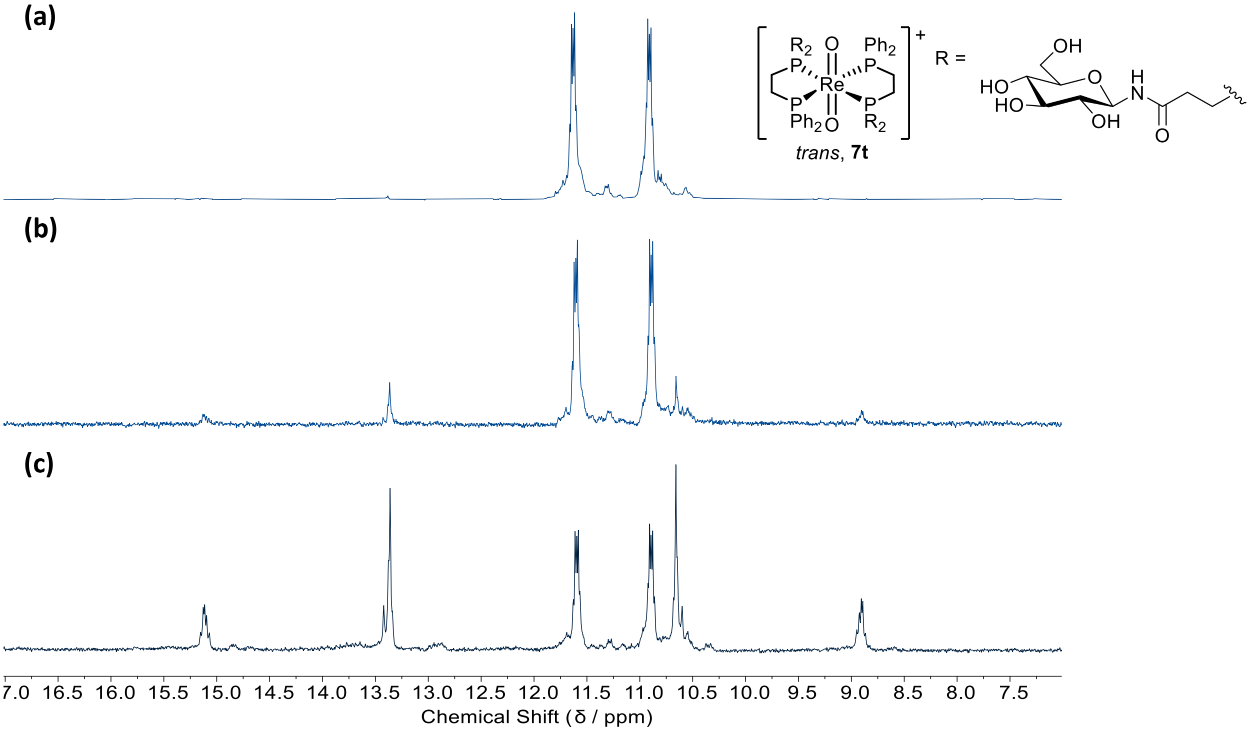

Figure S6 $^{31}P\{^{1}H\}$ NMR (202 MHz, CD_3OD) spectra of *trans*-$[Re(O)_2(\mathbf{L5})_2]^+$, **7t**, (a) freshly purified (b) after 4 days in solution and (c) after approx. 5 weeks in solution.

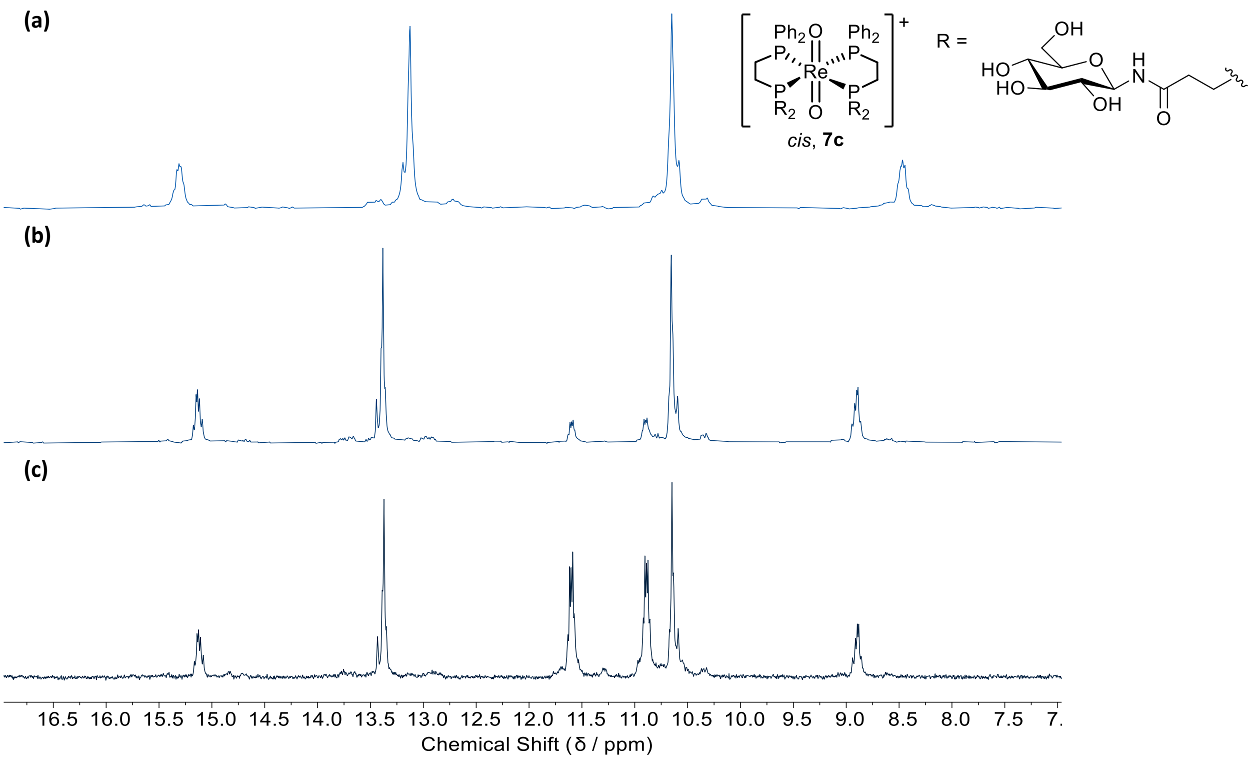

Figure S7 $^{31}P\{^{1}H\}$ NMR (162 MHz for (a) and 202 MHz for (b) and (c), CD_3OD) spectra of *cis*-$[Re(O)_2(\mathbf{L5})_2]^+$, **7c**, (a) freshly purified (b) after 4 days in solution and (c) after approx. 5 weeks in solution.

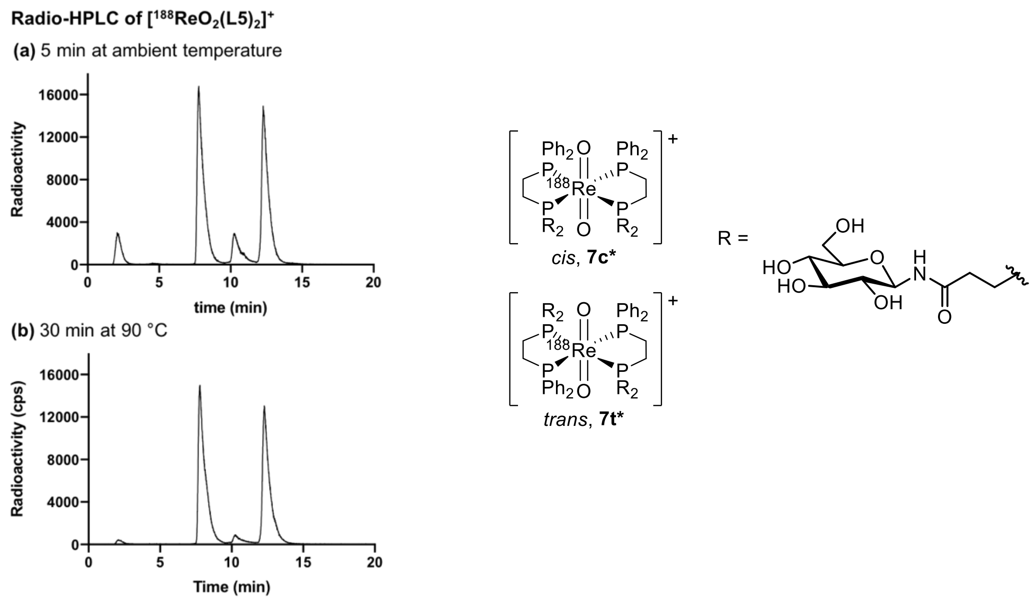

Figure S8 Analytical reverse-phase HPLC chromatograms of radiolabeling reactions between **L5** and ^{188}Re(V)-citrate to form $[^{188}Re(O)_2(\mathbf{L5})_2]^+$, **7c*** and **7t***, at (a) ambient temperature and 5 min reaction time; (b) 90 °C and 30 min reaction time.

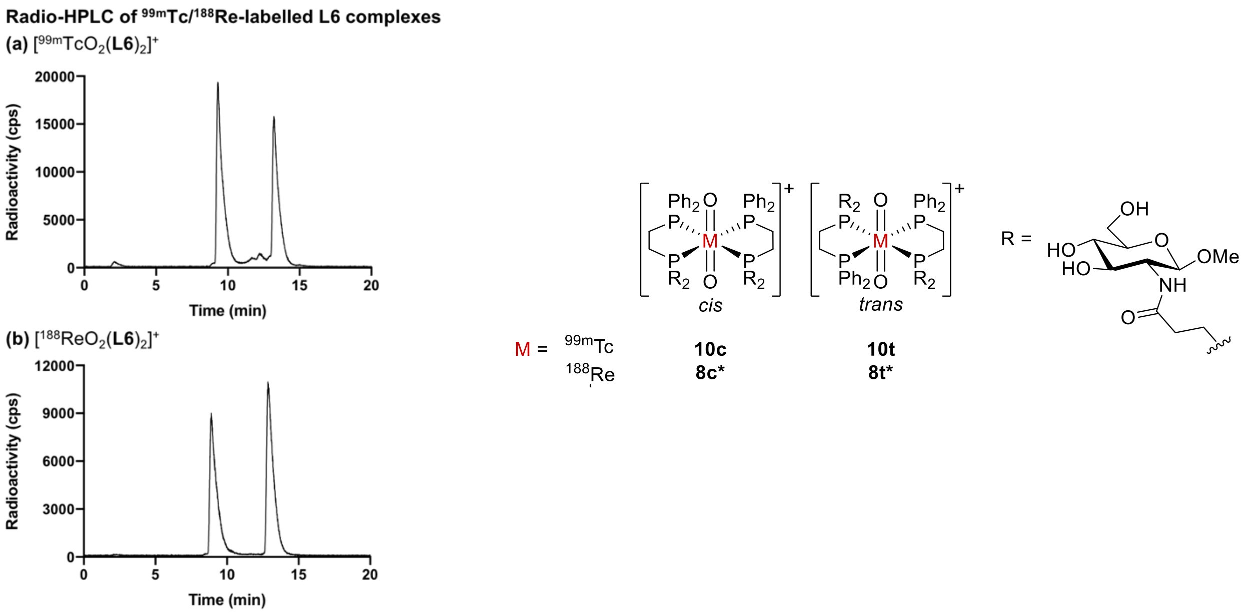

Figure S9 Analytical reverse-phase HPLC chromatograms of (a) [^{99m}Tc(O)$_2$(**L6**)$_2$]$^+$, **10c** and **10t**; (b) [^{188}Re(O)$_2$(**L6**)$_2$]$^+$, **8c*** and **8t***. The two signals in each chromatogram are putatively assigned to *trans* and *cis* geometric isomers of each complex.

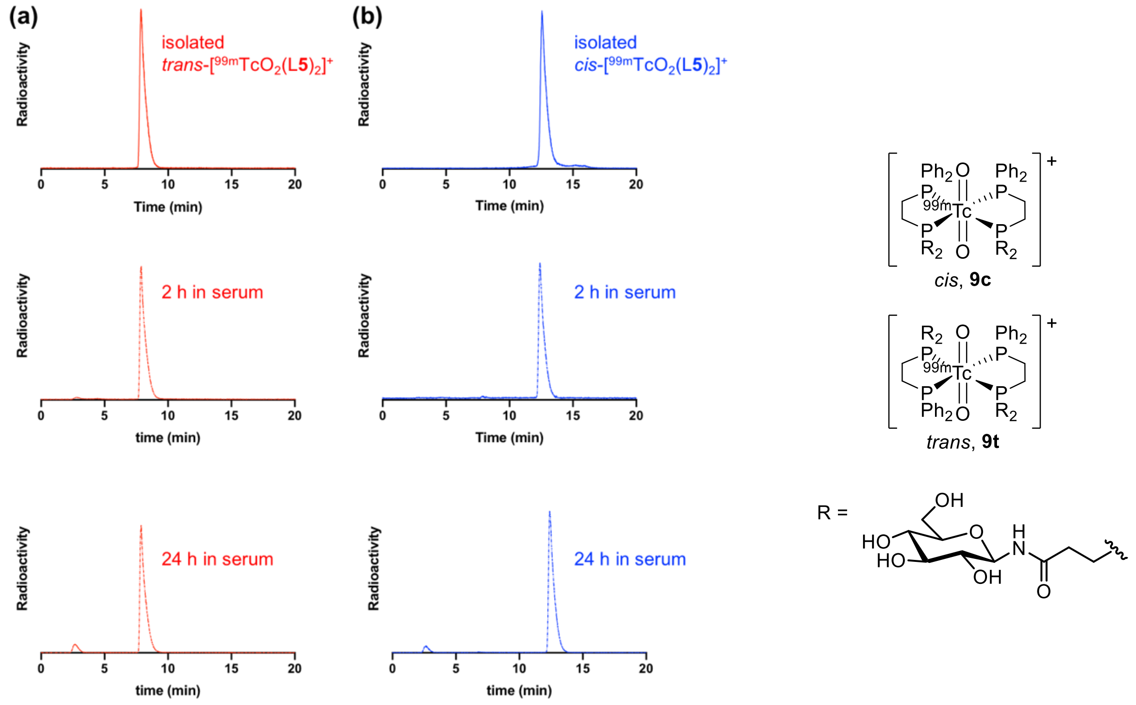

Figure S10 Analytical reverse-phase HPLC chromatography was used to assess the stability of both (a) *trans*-[^{99m}Tc(O)$_2$(**L5**)$_2$]$^+$, **9t**, and (b) *cis*-[^{99m}Tc(O)$_2$(**L5**)$_2$]$^+$, **9c**, after their incubation in serum at 37 °C for either 2 h or 24 h.

2 GENERAL EXPERIMENTAL

All reactions were carried out under a pre-purified nitrogen atmosphere, where the solvents are stated as dry or deoxygenated, using standard Schlenk line techniques. All air and moisture sensitive compounds were manipulated and stored in an Ar-atmosphere glovebox. DCM and MeCN were dried by Grubbs-type solvent purification system with activated alumina columns and deoxygenated by 3 freeze-pump-thaw cycles or bubbling with N_2 for 30 min. MeOH, ^{i}PrOH and ^{t}BuOH were dried over 3 or 4 Å molecular sieves and deoxygenated by bubbling with N_2 for 30 min. Bottled CD_2Cl_2 was purchased from Sigma-Aldrich and stirred with calcium hydride overnight, distilled, deoxygenated by 3 freeze-pump-thaw cycles and stored over 3 Å molecular sieves. Bottled $CDCl_3$, CD_3OD and DMSO-d_6 were purchased from Sigma-Aldrich, deoxygenated by bubbling with N_2 for 30 min and dried over 3 or 4 Å molecular sieves. β-D-glucopyranosylammonium carbamate,[1] $Ph_2PCH_2CH_2PH_2$ [2] and [Pt(nbe)$_3$][3] were synthesised according to literature methods and the latter two stored in an Ar-atmosphere glovebox. Other commercial reagents and chemicals were used without further purification unless otherwise stated. TLC visualisation was performed by examination under UV light (254 nm) and/or charring with 10% H_2SO_4 in EtOH for carbohydrate containing molecules.

^{1}H, ^{13}C and ^{31}P spectra were recorded on Jeol ECP(Eclipse) 300, Jeol ECS300, Jeol ECS400, Jeol ECZ400 (Jastec or Varian magnet), Bruker AV400, Varian VNMRS 500 or Bruker cryo500 MHz spectrometers. Chemical shifts (δ) are reported in parts per million (ppm) and coupling constants (*J*) in Hz. Chemical shifts for ^{1}H and ^{13}C spectra are referenced to residual solvent peaks, while ^{31}P NMR spectra are reported relative to 85% H_3PO_4 as an external standard. Two-dimensional NMR experiments were routinely used to confirm NMR assignments. Mass spectra were obtained by the University of Bristol Mass Spectrometry Service using an ESI (Bruker Daltonics micrOTOF II), Nanospray (Waters Synapt G2S), or MALDI (Bruker ultrafleXtreme 2) spectrometer. *X*-ray crystallography was performed by the University of Bristol Crystallography Service using a Bruker Kappa Apex II diffractometer. Optical rotations were recorded on an ADP220 polarimeter (Bellingham and Stanley) using the sodium D line (λ = 589 nm).

Purification by preparative reverse-phase HPLC on a Grace Discovery Sciences Reveleris Prep System with a Phenomenex Luna C18(2) AXIA packed column (250 x 21.2 mm, 5 µM particle size, 100 Å pore size), with flow rate of 14 mL min^{-1} and monitoring via ELSD and UV detection at 200, 220 and 254 nm wavelengths. Specific gradient mobile phases are described below using solvent A (0.1% trifluoroacetate in water) and solvent B (0.1% trifluoroacetate in acetonitrile).

Throughout this work, complexes of the general type [M(O)$_2$(diphos)$_2$]$^+$, where M = Re or Tc, *cis* and *trans* refers to the geometry of the diphosphine ligands, not the *trans*-MO_2 dioxo core.

Below are schemes detailing the synthetic routes to the *C*1 and *C*2 glucose acrylamides.

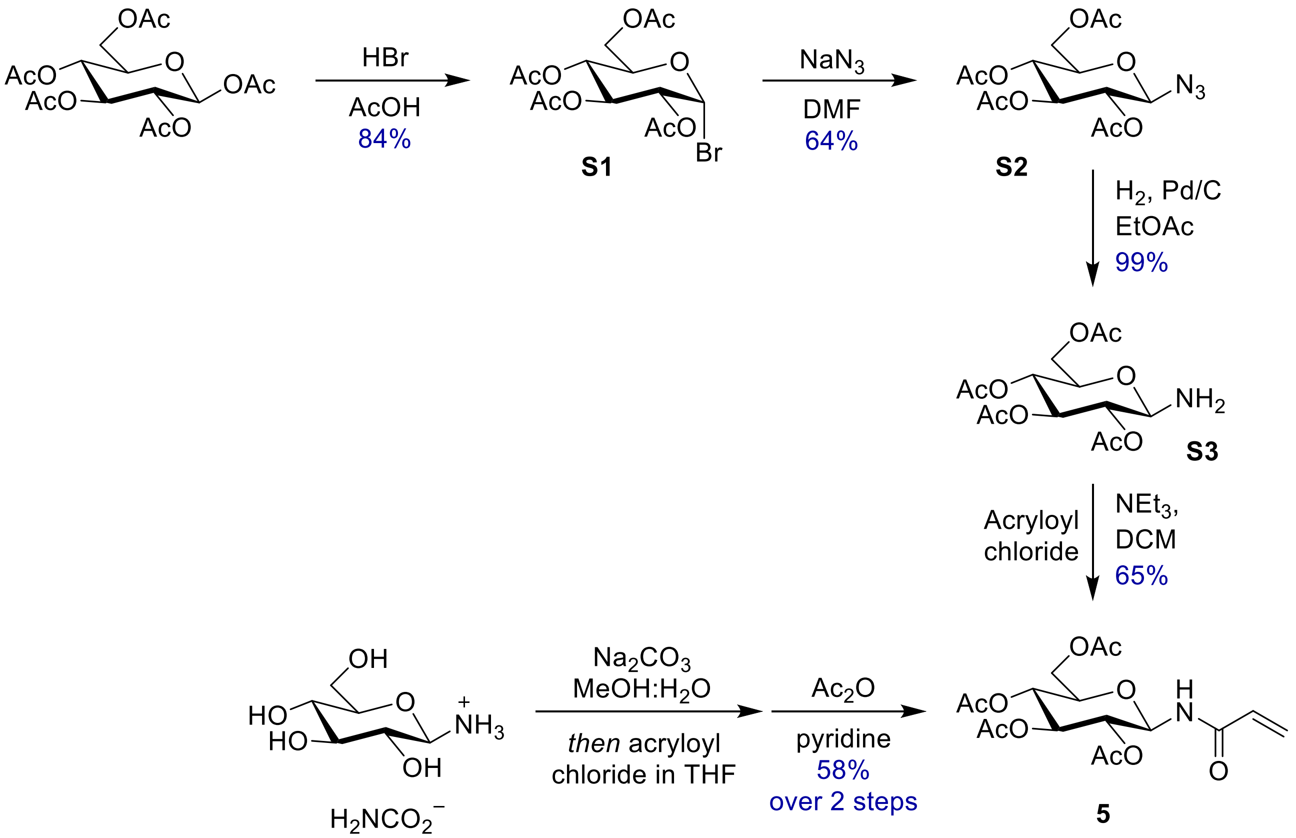

Scheme S1: The initial (top) and later (bottom) synthesis of *C*1-acrylamide derivative of glucose, **5**. The bottom route avoids the use of azides and is therefore safer for scaling up.

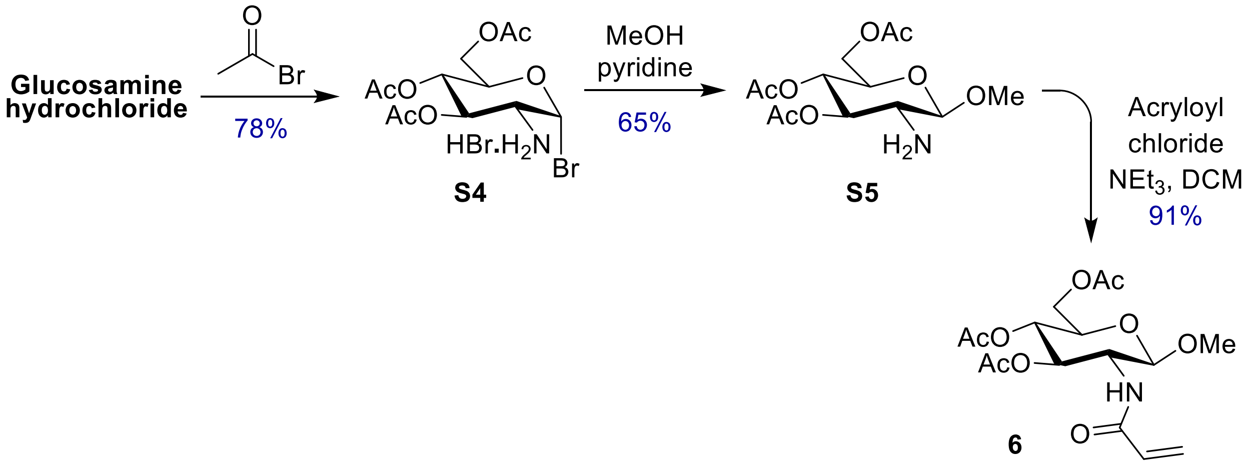

Scheme S2: Synthesis of *C*2-acrylamide derivative of glucose, **6**.

3 EXPERIMENTAL PROCEDURES

3.1.1 Synthesis of $Ph_2PCH_2CH_2P(CH_2CH_2CO_2Me)_2$, **L1**

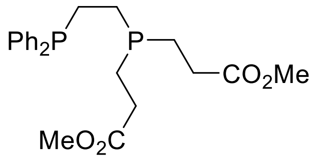

To a solution of $Ph_2PCH_2CH_2PH_2$ (100 mg, 0.406 mmol) in dry DCM (2 mL) was added [Pt(nbe)$_3$] (9.00 mg, 0.0200 mmol) and stirred at room temperature to afford a deep orange solution. After 10 min, ^{t}BuOH (8.10 mmol, 0.8 mL) was added, followed by dropwise addition of methyl acrylate (73 µL, 0.81 mmol) and the mixture was left to stir for 2 h at room temperature. The reaction mixture was passed through a silica plug and the filtrate concentrated to dryness to give **L1** as a colourless oil (122 mg, 0.292, 72%). **^{31}P{^{1}H} NMR** (162 MHz, CD_2Cl_2): δ_P (ppm) −12.8 (d, $^3J_{P,P}$ = 29.0 Hz, PPh_2), −20.7 (d, $^3J_{P,P}$ = 28.0 Hz, $P(CH_2CH_2CO_2Me)_2$). **^{1}H NMR** (400 MHz, CD_2Cl_2): δ_H (ppm) 7.44–7.38 (m, 4H, Ar-*H*), 7.38–7.30 (m, 6H, Ar-*H*), 3.63 (s, 6H, Me), 2.39–2.30 (m, 4H, $P(CH_2CH_2CO_2Me)_2$), 2.16–2.08 (m, 2H, Ph_2PCH_2), 1.72–1.66 (m, 4H, $P(CH_2CH_2CO_2Me)_2$), 1.51–1.43 (m, 2H, $Ph_2PCH_2CH_2P$). The spectroscopic data is in accordance with the literature.[4]

3.1.2 Synthesis of [Re(O)$_2$(**L1**)$_2$]I, **1c** and **1t**

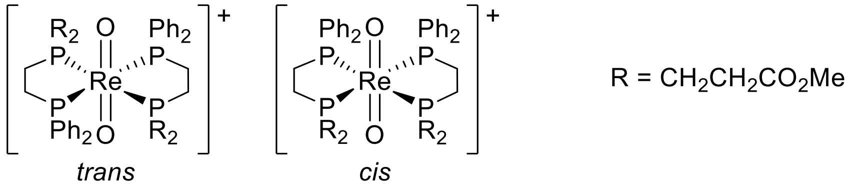

To a solution of [ReI(O)$_2$(PPh$_3$)$_2$] (25.0 mg, 29.0 µmol) in dry DCM (2 mL) was added **L1** (24.0 mg, 57.0 µmol). The solution turned from violet to dark orange and was left to stir for 10 min. The solution was concentrated to dryness and the resulting dark yellow precipitate was washed sequentially with pentane (3 x 1 mL) and toluene (3 x 1 mL). The product was obtained as mixture of *trans* and *cis* isomers, with respect to the diphosphine ligands (26.0 mg, 24.6 µmol, 85%). Yellow crystals of the *trans* complex suitable for *X*-ray crystallography were obtained *via* vapour diffusion recrystallization from methanol and pentane. **^{31}P{^{1}H} NMR** (162 MHz, CD_2Cl_2): δ_P (ppm) 11.6 (m), 9.0 (m). **^{1}H NMR** (400 MHz, CD_2Cl_2): δ_H (ppm) 7.68 (m, 8H, Ar-*H*), 7.58 (m, 4H, Ar-*H*), 7.51 (m, 8H, Ar-*H*), 3.55 (s, 12H, CH_3), 2.73 (m, 4H, Ph_2PCH_2), 2.30 (m, 8H, $CH_2CH_2CO_2Me$), 2.01 (m, 8H, $CH_2CH_2CO_2Me$), 1.69 (m, 4H, $Ph_2PCH_2CH_2P$). **MS** (ESI+) *m*/*z* calcd. for $C_{44}H_{56}O_{10}P_4Re$ ([*M*+H]$^+$) = 1054.6; obs. = 1055.2.

3.1.3 Synthesis of $Ph_2PCH_2CH_2P(CH_2CH_2CO_2Na)_2$, **L2-Na$_2$**

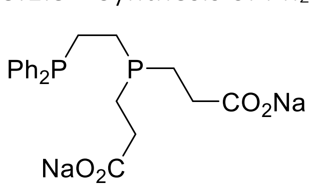

To a solution of **L1** (849 mg, 2.03 mmol) in deoxygenated MeOH:H_2O (1:1, 20 mL) was added sodium hydroxide (162 mg, 4.06 mmol) at room temperature and stirred for 16 h. The solvent was removed *in vacuo* to yield the title product as a white crystalline solid (700 mg, 1.61 mmol, 79%). **^{31}P{^{1}H} NMR** (162 MHz, CD_3OD): δ_P (ppm) −12.4 (d, $^3J_{P,P}$ = 26.9 Hz, *P*Ph$_2$), −22.5 (d, $^3J_{P,P}$ = 26.9 Hz, *P*$(CH_2CH_2CO_2Me)_2$). **^{1}H NMR** (400 MHz, CD_3OD): δ_H (ppm) 7.44–7.39 (m, 4H, Ar-*H*), 7.36–7.30 (m, 6H, Ar-*H*), 2.27–2.14 (m, 6H, Ph_2PCH_2, $P(CH_2CH_2CO_2Na)_2$), 1.77–1.68 (m, 4H, $P(CH_2CH_2CO_2Na)_2$), 1.50–1.42 (m, 2H, $Ph_2PCH_2CH_2P$). **^{13}C{^{1}H} NMR** (162 MHz, CD_3OD): δ_C (ppm) 182.2 (d, $^3J_{P,C}$ = 12.3 Hz, *C*=O), 139.9 (d, $^1J_{P,C}$ = 13.7 Hz, Ar*C*), 133.8 (d, $^2J_{P,C}$ = 18.5 Hz, *o*-Ar*C*H), 129.7 (s, *p*-Ar*C*H), 129.6 (d, $^3J_{P,C}$ = 6.6 Hz, *m*-Ar*C*H), 35.1 (d, $^2J_{P,C}$ = 13.7 Hz, $P(CH_2CH_2CO_2Na)_2$), 24.6 (t, $J_{P,C}$ = 12.8 Hz, Ph_2PCH_2), 24.4 (d, $^1J_{P,C}$ = 12.4 Hz,

P($CH_2CH_2CO_2Na)_2$), 23.3 (t, $J_{P,C}$ = 14.8 Hz, $Ph_2PCH_2CH_2P$). **HR-MS** (Nano) *m/z* calcd. for $C_{20}H_{22}O_4P_2$ ([*M*+H]$^-$) = 389.1072; obs. = 389.1079.

3.1.4 Synthesis of *cis*- and *trans*-[Re(O)$_2$(**L2-H$_2$**)$_2$]$^+$, **2c** and **2t**

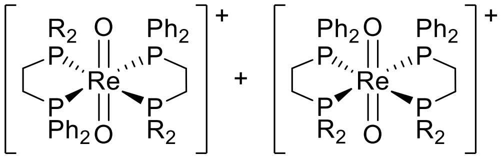

trans *cis*

R = $CH_2CH_2CO_2H$

To a solution of [ReI(O)$_2$(PPh$_3$)$_2$] (50.1 mg, 57.6 µmol) in dry DCM (3 mL) was added **L2-Na$_2$** (50.0 mg, 0.115 mmol). The solution turned from violet to clear yellow and was left to stir for 2 h. The solution was concentrated to dryness and the resulting dark yellow precipitate was washed sequentially with diethyl ether (3 x 1 mL). The product was obtained as mixture of *trans* and *cis* isomers, with respect to the diphosphine ligands **L2-Na$_2$** (54.3 mg, 44.7 µmol, 78%).

A portion of the mixture of *trans* and *cis* isomers was separated by reverse-phase prep-HPLC (5 min at 5% solvent B, 20 min gradient to 40% solvent B, 10 min ramp to 95% solvent B) and characterised separately: t_R (*trans*) = 28.5 min, t_R (*cis*) = 32.4 min. It was assumed that after HPLC purification using acidified eluents, the carboxylates would be protonated (**L2-H$_2$**).

The $^{31}P\{^1H\}$ NMR spectrum of the *cis* complex was simulated in Mestrenova (see Figure 2), and the *J* values are given in the experimental data below. Due to the lack of information visible in the $^{31}P\{^1H\}$ NMR spectrum of the *trans* complex (only 5 of the 12 possible lines for each half of the AA'BB' spin system), calculation of the exact *J* values of the *trans* complex **2t** has not been achieved thus far. The **2t** simulation was particularly insensitive to changes in the *trans*-P atom coupling, and the observed pattern could only be achieved through $J_{AA'}$ = $J_{BB'}$ = large (>100 Hz) and the rest small (< ±10 Hz). Using the numerical values obtained for the *cis* complex (*e.g.* the large *trans*-P splitting, $J_{AB'}$ = $J_{A'B}$ = 352 Hz, for **2c** was used for **2t** $J_{AA'}$ and $J_{BB'}$) a reasonable simulation fit was achieved, and these values are quoted below:

$J_{AA'}$ = $J_{BB'}$ = +352 Hz
J_{AB} = $J_{A'B'}$ = +4.7 Hz
$J_{AB'}$ = $J_{A'B}$ = −9.3 Hz

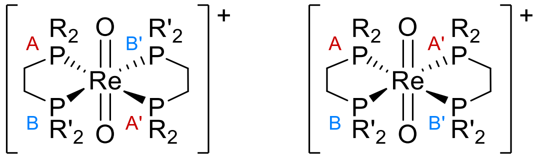

trans *cis*

$J_{AA'}$ = $J_{BB'}$ = −9.3 Hz
J_{AB} = $J_{A'B'}$ = +4.7 Hz
$J_{AB'}$ = $J_{A'B}$ = +352 Hz

trans-[Re(O)$_2$(**L2-H$_2$**)$_2$]$^+$, **2t**: **^{31}P{^{1}H} NMR** (121 MHz CD$_3$OD): δ_P (ppm) 11.3 (m, $J_{AA'}$ = 352 Hz, J_{AB} = $J_{A'B'}$ = 4.7 Hz, $J_{AB'}$ = $J_{A'B}$ = −9.3 Hz), 9.7 (m, $J_{BB'}$ = 352 Hz, J_{AB} = $J_{A'B'}$ = 4.7 Hz, $J_{AB'}$ = $J_{A'B}$ = −9.3 Hz). **^{1}H NMR** (500 MHz CD$_3$OD): δ_H (ppm) 7.81–7.75 (m, 8H, Ar-*H*), 7.64–7.53 (m, 12H, Ar-*H*), 2.96–2.85 (m, 4H, C*H*$_2$), 2.49–2.38 (4H, C*H*$_2$), 2.30–2.20 (m, 4H, C*H*$_2$), 2.18–2.04 (m, 8H, C*H*$_2$), 1.83–1.73 (m, 4H, C*H*$_2$). **^{13}C{^{1}H} NMR** (125 MHz, CD$_3$OD): δ_C (ppm) 175.0 (t, *C*=O), 134.5 (t, Ar*C*), 133.5 (s, Ar*C*), 130.7 (s, Ar*C*), 130.5 (t, Ar*C*), 31.5 (m, *C*H$_2$), 28.6 (s, *C*H$_2$), 22.1 (m, *C*H$_2$), 21.1 (t, *C*H$_2$). **HR-MS** (Nano) *m/z* calcd. for $C_{40}H_{46}O_{10}P_4{}^{187}Re$ ([*M*−2H]$^-$) 997.1599; obs. = 997.1611.

cis-[Re(O)$_2$(**L2-H$_2$**)$_2$]$^+$, **2c**: **^{31}P{^{1}H} NMR** (121 MHz CD$_3$OD): δ_P (ppm) 12.4 (m, $J_{AB'}$ = $J_{A'B}$ = 352 Hz, J_{AB} = $J_{A'B'}$ = 4.7 Hz, $J_{AA'}$ = −9.3 Hz), 9.8 (m, $J_{AB'}$ = $J_{A'B}$ = 352 Hz, J_{AB} = $J_{A'B'}$ = 4.7 Hz, $J_{BB'}$ = −9.3 Hz). **^{1}H NMR** (400

MHz CD_3OD): δ_H (ppm) 7.43 (t, 4H, Ar-*H*), 7.33–7.21 (m, 16H, Ar-*H*), 2.91–2.66 (m, 12H, CH_2), 2.65–2.47 (m, 8H, CH_2), 2.38-2.23 (m, 4H, CH_2). **$^{13}C\{^1H\}$ NMR** (101 MHz, CD_3OD): δ_C (ppm) 175.3 (m, *C*=O), 134.5 (m, Ar*C*), 132.8 (s, Ar*C*), 130.0 (m, Ar*C*), 29.5 (m, CH_2 observed by HSQC), 28.7 (s, CH_2), 22.8 (m, CH_2, CH_2). **HR-MS** (ESI+) *m/z* calcd. for $C_{40}H_{46}O_{10}P_4{}^{187}Re$ ($[M{-}2H]^-$) 997.1599; obs. = 997.1621.

3.1.5 Synthesis of 2,3,4,6-tetra-*O*-acetyl-α-D-glucopyranosyl bromide, S1

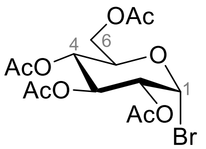

The title compound (3.52 g, 8.56 mmol, 84% as 4:1 ratio of α:β) was synthesised from peracetylated glucose according to the literature procedure.[5] **^1H NMR** (400 MHz, $CDCl_3$): δ_H (ppm) 6.60 (d, 1H, *J* = 4.0 Hz, *H*-1), 5.55 (t, 1H, *J* = 9.7 Hz, *H*-3), 5.15 (t, 1H, *J* = 9.8 Hz, *H*-4), 4.83 (dd, 1H, *J* = 10.0, 4.0 Hz, *H*-2), 4.36–4.20 (m, 2H, *H*-5, *H*-6a), 4.16–4.08 (m, 1H, *H*-6b), 2.09–2.02 (4 x s, 12H, CH_3CO_2). **$^{13}C\{^1H\}$ NMR** (101 MHz, $CDCl_3$): δ_C (ppm) 170.7–169.6 (4 x s, CH_3*C*O_2), 86.9 (s, *C*-1), 72.3 (s, *C*-5), 70.7 (s, *C*-2), 70.3 (s, *C*-3), 67.3 (s, *C*-4), 61.1 (s, *C*-6), 20.8–20.6 (4 x s, *C*H_3CO_2). The spectroscopic data is in accordance with the literature.[6] *The* β *anomer was observed in the ^1H NMR spectrum at ca. 20%, and was removed during purification at the next step.*

3.1.6 Synthesis of 1-azido-1-deoxy-2,3,4,6-tetra-*O*-acetyl-β-D-glucopyranose, S2

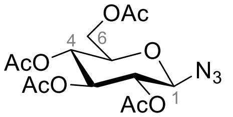

The title compound (583 mg, 1.56 mmol, 64%) was synthesised from **S1** according to the literature procedure.[7] **^1H NMR** (400 MHz, $CDCl_3$): δ_H (ppm) 5.21 (t, 1H, *J* = 9.5 Hz, *H*-3), 5.09 (t, 1H, *J* = 9.7 Hz, *H*-4), 4.95 (t, 1H, *J* = 9.2 Hz, *H*-2), 4.64 (d, 1H, *J* = 8.9 Hz, *H*-1), 4.26 (dd, 1H, *J* = 12.5, 4.8 Hz, *H*-6a), 4.16 (dd, 1H, *J* = 12.5, 2.3 Hz, *H*-6b), 3.79 (ddd, 1H, *J* = 10.1, 4.8, 2.3 Hz, *H*-5), 2.09–2.00 (4 x s, 12H, CH_3CO_2). **$^{13}C\{^1H\}$ NMR** (101 MHz, $CDCl_3$): δ_C (ppm) 170.7–169.3 (4 x s, CH_3*C*O_2), 88.0 (s, *C*-1), 74.2 (s, *C*-5), 72.7 (s, *C*-3), 70.8 (s, *C*-2), 68.0 (s, *C*-4), 61.8 (s, *C*-6), 21.0–20.4 (4 x s, *C*H_3CO_2). The spectroscopic data is in accordance with the literature.[7]

3.1.7 Synthesis of 1-amino-1-deoxy-2,3,4,6-tetra-*O*-acetyl-β-D-glucopyranose, S3

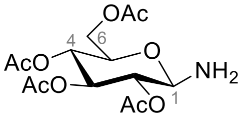

Palladium on carbon (20 wt%, 110 mg) was added to a stirred solution of azide **S2** (558 mg, 1.49 mmol) in deoxygenated EtOAc (20 mL). The reaction mixture was stirred under an atmosphere of H_2 (1 atm, balloon) at room temperature for 18 h. After purging with N_2, the reaction mixture was filtered over a pad of celite, eluting with MeOH. The filtrate was concentrated and azeotroped with EtOAc (3 x 20 mL) to afford the title compound (515 mg, 1.48 mmol, 99%) as a white solid. **^1H NMR** (400 MHz, $CDCl_3$): δ_H (ppm) 5.23 (t, 1H, *J* = 9.6 Hz, *H*-3), 5.02 (dd, 1H, *J* = 10.1, 9.4 Hz, *H*-4), 4.81 (dd, 1H, *J* = 9.7, 9.0 Hz, *H*-2), 4.24–4.15 (m, 2H, *H*-1, *H*-6a), 4.09 (dd, 1H, *J* = 12.3, 2.3 Hz, *H*-6b), 3.68 (ddd, 1H, *J* = 10.1, 4.9, 2.4 Hz, *H*-5), 2.09–1.98 (4 x s, 12H, CH_3CO_2). **$^{13}C\{^1H\}$ NMR** (101 MHz, $CDCl_3$): δ_C (ppm) 170.9–169.6 (4 x s, CH_3*C*O_2), 85.1 (s, *C*-1), 73.3 (s, *C*-3), 72.9 (s, *C*-5), 72.2 (s, *C*-2), 68.9 (s, *C*-4), 62.4 (s, *C*-6), 21.1–20.6 (4 x s, *C*H_3CO_2). The spectroscopic data is in accordance with the literature.[8]

3.1.8 Synthesis of 1-acrylamido-1-deoxy-2,3,4,6-tetra-*O*-acetyl-β-D-glucopyranose, 5

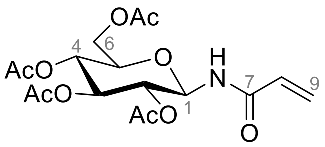

Triethylamine (135 µL, 1.70 mmol) was added to a solution of amine **S3** (493 mg, 1.42 mmol) in dry DCM (3.6 mL) and cooled to 0 °C. Acryloyl chloride (135 µL, 1.70 mmol) was added dropwise, and the reaction mixture allowed to warm to RT. After stirring for 3 h at RT the volatiles were removed *in vacuo*. The crude product was purified by column chromatography (70%

EtOAc in pet. ether) to afford the title compound (371 mg, 0.924 mmol, 65%) as a white foam. **^{1}H NMR** (400 MHz, $CDCl_3$): δ_H (ppm) 6.44 (d, 1H, *J* = 9.3 Hz, NH), 6.29 (dd, 1H, *J* = 17.1, 1.1 Hz, *H*-9a), 6.05 (dd, 1H, *J* = 17.1, 10.4 Hz, *H*-8), 5.73 (dd, 1H, *J* = 10.4, 1.1 Hz, *H*-9b), 5.35–5.29 (m, 2H, *H*-1, *H*-3), 5.07 (dd, 1H, *J* = 10.1, 9.4 Hz, *H*-4), 4.95 (t, 1H, *J* = 9.6 Hz, *H*-2), 4.31 (dd, 1H, *J* = 12.5, 4.4 Hz, *H*-6a), 4.08 (dd, 1H, *J* = 12.5, 2.2 Hz, *H*-6b), 3.85 (ddd, 1H, *J* = 10.1, 4.4, 2.2 Hz, *H*-5), 2.09–1.98 (4 x s, 12H, CH_3CO_2). **$^{13}C\{^1H\}$ NMR** (101 MHz, $CDCl_3$): δ_C (ppm) 171.3–169.7 (4 x s, CH_3CO_2), 165.6 (s, *C*-7), 130.1 (s, *C*-8), 128.7 (s, *C*-9), 78.5 (s, *C*-1), 73.8 (s, *C*-5), 72.8 (s, *C*-3), 70.8 (s, *C*-2), 68.3 (s, *C*-4), 61.8 (s, *C*-6), 21.0–20.5 (4 x s, CH_3CO_2). The spectroscopic data is in accordance with the literature.[9]

Alternative Synthesis of **5**: The following were synthesised according to the adapted literature procedure.[1] Freshly dried β-D-glucopyranosylammonium carbamate (1.2 g, 5.00 mmol) and Na_2CO_3 (3.00 g, 28.3 mmol) were dissolved in MeOH:H_2O (60 mL, 1:1) and cooled to 0 °C. After 30 min, a solution of acryloyl chloride (1.28 mL, 16.1 mmol) in THF (7 mL) was added dropwise. After stirring for a further 1 h at 0 °C, the solution was concentrated under reduced pressure, triturated with MeOH (*ca.* 50 mL) and the filtrate concentrated to afford the crude *N*-(prop-2-enoyl)-β-D-galactopyranosylamine as a white solid. Acetic anhydride (7.7 mL) was then added to a solution of crude *N*-(prop-2-enoyl)-β-D-galactopyranosylamine and 4-dimethylaminopyridine (*ca.* 15 mg) in pyridine (15 mL) at 0 °C. The solution was allowed to warm to RT and stirred overnight. The reaction mixture was then concentrated, dissolved in DCM (20 mL) and washed with 1 M HCl (20 mL). The organic layer was then washed with H_2O (3 x 10 mL), dried over $MgSO_4$ and concentrated. The crude product was then purified by flash column chromatography (70% EtOAc in pet. ether) to afford the title compound **5** (1.17 g, 2.92 mmol, 58% over 2 steps) as a white solid.

3.1.9 Synthesis of protected $Ph_2PCH_2CH_2P(CH_2CH_2CONH^{C1}Glc)_2$, **L3**

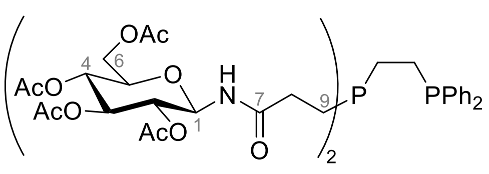

To a solution of $Ph_2PCH_2CH_2PH_2$ (25.0 mg, 0. mmol) in dry DCM (0.4 mL) was added [Pt(nbe)$_3$] (1.2 mg, 2.51 µmol) and stirred at room temperature to afford a deep orange solution. After 10 min, ^{i}PrOH (2.03 mmol, 0.16 mL) was added, followed by dropwise addition of acrylamide **5** (81.5 mg, 0.203 mmol) in DCM (0.4 mL) and the mixture was left to stir at room temperature. The crude product was purified by flash column chromatography (1% MeOH and 1% NEt_3 in DCM) to afford the product (87.9 mg, 83.8 µmol, 82%) as a white solid. **$^{31}P\{^1H\}$ NMR** (162 MHz, $CDCl_3$): δ_P (ppm) −12.4 (d, $^3J_{P,P}$ = 28.0 Hz, *P*Ph$_2$), −23.2 (d, $^3J_{P,P}$ = 28.0 Hz, *P*R$_2$). **^{1}H NMR** (400 MHz, $CDCl_3$): δ_H (ppm) 7.42–7.35 (m, 4H, Ar-*H*), 7.34–7.28 (m, 6H, Ar-*H*), 6.65 (d, 2H, *J* = 9.4, NH), 5.30 (q, 2H, *J* = 9.5 Hz, *H*-3), 5.24 (td, 2H, *J* = 9.5, 2.2 Hz, *H*-1), 5.07 (ddd, 2H, *J* = 10.4, 9.4, 1.2 Hz, *H*-4), 4.94 (td, 2H, *J* = 9.5, 1.7 Hz, *H*-2), 4.30 (ddd, 2H, *J* = 12.5, 4.4, 1.6 Hz, *H*-6a), 4.06 (dd, 2H, *J* = 12.5, 2.2 Hz, *H*-6b), 3.82 (ddd, 2H, *J* = 9.7, 4.7, 2.4 Hz, *H*-5), 2.31–1.92 (m, 30H, *H*-8/*H*-9, 8 x CH_3CO_2, Ph_2PCH_2), 1.76–1.53 (m, 4H, *H*-8/*H*-9), 1.50–1.35 (m, 2H, $Ph_2PCH_2CH_2$). **^{13}C NMR** (101 MHz, $CDCl_3$): δ_C (ppm) 172.9 (m, 2 x *C*-7), 171.2 (2 x s, 2 x CH_3CO_2), 170.7 (s, 2 x CH_3CO_2), 170.0 (s, 2 x CH_3CO_2), 169.7 (s, 2 x CH_3CO_2), 138.0 (m, Ar*C*), 132.8, (2 x d, $^2J_{P,C}$ = 18.5 Hz, *o*-Ar*C*H), 128.9 (2 x s, *p*-Ar*C*H), 128.7 (2 x d, $^3J_{P,C}$ = 6.4 Hz, *m*-Ar*C*H), 78.2 (2 x s, *C*-1), 73.7 (s, *C*-5), 72.8 (2 x s, *C*-3), 70.9 (2 x s, *C*-2), 68.2 (2 x s, *C*-4), 61.8 (2 x s, *C*-6), 32.4 (m, *C*-8/*C*-9), 23.6 (t, *J* = 13.7 Hz, CH_2), 22.2 (t, *J* = 15.0 Hz, CH_2), 21.9–20.3 (m, *C*-8/*C*-9, 6 x CH_3CO_2). **HR-MS** (Nanospray): *m/z* calcd. for $C_{48}H_{63}N_2O_{20}P_2$ ([*M*+H]$^+$) = 1049.3449; obs. = 1049.3446.

3.1.10 Synthesis of $Ph_2PCH_2CH_2P(CH_2CH_2CONH^{C1}Glc)_2$, **L5**

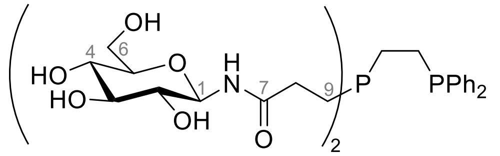

NaOMe in MeOH (56 µL, 25 wt% in MeOH) was added to a stirred solution of **L3** (205 mg, 0.195 mmol) in deoxygenated MeOH (11 mL). After the reaction had reached completion, as monitored by TLC-MS, the reaction was neutralised by the addition of Amberlite IR 120. The reaction mixture was then filtered and concentrated *in vacuo* to give the title compound (103 mg, 145 µmol, 74%) as a white solid. **$^{31}P\{^1H\}$ NMR** (162 MHz, CD_3OD): δ_P (ppm) −12.8 (d, $^3J_{P,P}$ = 29.2 Hz, *P*Ph_2), −22.6 (d, $^3J_{P,P}$ = 29.2 Hz, *P*R_2). **^1H NMR** (400 MHz, D_2O): δ_H (ppm) 7.39–7.26 (m, 4H, Ar-*H*), 7.24–7.10 (m, 6H, Ar-*H*), 5.00–4.93 (m, 2H, *H*-1), 3.91–3.99 (m, 4H, *H*-6a/b), 3.61–3.36 (m, 8H, *H*-2, *H*-3, *H*-4, *H*-5), 2.39–2.22 (m, 4H, *H*-8), 2.15–2.00 (m, 2H, Ph_2PCH_2), 1.73–1.56 (m, 4H, *H*-9), 1.46–1.33 (m, 2H, $Ph_2PCH_2CH_2$). **^{13}C NMR** (101 MHz, CD_3OD): δ_C (ppm) 176.6 (d, $^3J_{P,C}$ = 10.7 Hz, *C*-7), 139.7 (d, $J_{P,C}$ = 13.5 Hz, Ar*C*), 133.8, (d, $^2J_{P,C}$ = 18.6 Hz, *o*-Ar*C*H), 129.9 (s, *p*-Ar*C*H), 129.6 (d, $^3J_{P,C}$ = 6.5 Hz, *m*-Ar*C*H), 81.1 (s, *C*-1), 79.6 (s), 78.9 (s), 74.0 (s), 71.4 (s), 62.6 (s, *C*-6), 33.2 (d, *J* = 15.4 Hz, *C*-8), 24.6 (m, Ph_2PCH_2), 23.3 (m, $Ph_2PCH_2CH_2$), 22.4 (m, *C*-9). **HR-MS** (Nanospray): *m/z* calcd. for $C_{32}H_{47}N_2O_{12}P_2$ ($[M+H]^+$) = 713.2604; obs. = 713.2618. **$[\alpha]_D^{24.0}$** +4.34 (c = 1.38, MeOH).

3.1.11 Synthesis of *cis*- and *trans*-$[Re(O)_2(\mathbf{L5})_2]^+$, **7c** and **7t**

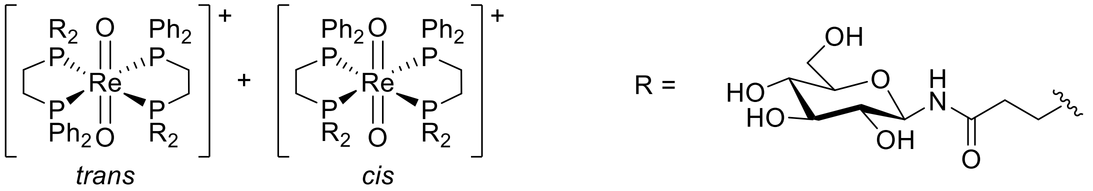

To a solution of $[ReI(O)_2(PPh_3)_2]$ (15.5 mg, 17.9 µmol) in dry MeOH (1 mL) was added **L5** (25.5 mg, 35.8 µmol) in dry MeOH (1 mL). The solution turned from violet to pale brown and was left to stir for 4 h. The solution was concentrated to *ca.* 0.5 mL and dry Et_2O (3 mL) was added to afford a pale brown precipitate. The supernatant was removed, the precipitate washed with Et_2O (2 x 2 mL) and then dried under vacuum. The product was obtained as mixture of *trans* and *cis* isomers, with respect to the diphosphine ligands, (31.1 mg, 17.6 µmol, 98%) as a pale brown solid. **$^{31}P\{^1H\}$ NMR** (162 MHz, CD_3OD): δ_P (ppm) 16.3–8.8 (m). **HR-MS** (Nanospray): *m/z* calcd. for $C_{64}H_{92}N_4O_{26}P_4{}^{187}Re$ ($[M]^+$) = 1643.4508; obs. = 1643.4539.

Purification of a portion of the mixture by reverse-phase prep-HPLC (20 min gradient of 5–30% solvent B) afforded the separated isomers: t_R (*trans*) = 10.4 min, t_R (*cis*) = 18.7 min. $^{31}P\{^1H\}$ NMR simulation was achieved using the same method and $J_{P,P}$ values as outlined for **2c/2t** (see Section 3.1.4), and the data are given below:

trans-$[Re(O)_2(\mathbf{L5})_2]^+$, **7t**: **$^{31}P\{^1H\}$ NMR** (202 MHz, CD_3OD): δ_P (ppm) 11.6 (m, $J_{AA'}$ = 352 Hz, $J_{AB} = J_{A'B'}$ = 4.7 Hz, $J_{AB'} = J_{A'B}$ = −9.3 Hz), 10.9 (m, $J_{BB'}$ = 352 Hz, $J_{AB} = J_{A'B'}$ = 4.7 Hz, $J_{AB'} = J_{A'B}$ = −9.3 Hz). **HR-MS** (Nanospray): *m/z* calcd. for $C_{64}H_{92}N_4O_{26}P_4{}^{187}Re$ ($[M]^+$) = 1643.4508; obs. = 1643.4529.

cis-$[Re(O)_2(\mathbf{L5})_2]^+$, **7c**: **$^{31}P\{^1H\}$ NMR** (202 MHz, CD_3OD): δ_P (ppm) 14.1 (m, $J_{AB'} = J_{A'B}$ = 352 Hz, $J_{AB} = J_{A'B'}$ = 4.7 Hz, $J_{AA'}$ = −9.3 Hz), 9.9 (m, $J_{AB'} = J_{A'B}$ = 352 Hz, $J_{AB} = J_{A'B'}$ = 4.7 Hz, $J_{BB'}$ = −9.3 Hz). **HR-MS** (Nanospray): *m/z* calcd. for $C_{64}H_{92}N_4O_{26}P_4{}^{187}Re$ ($[M]^+$) = 1643.4508; obs. = 1643.4529.

3.1.12 Synthesis of 2-amino-2-deoxy-3,4,6-tri-*O*-acetyl-α-D-glucopyranosyl bromide·HBr, S4

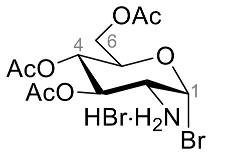

The title compound (2.44 g, 5.43 mmol, 78%) was synthesised according to the literature procedure.[10] **^{1}H NMR** (400 MHz, $CDCl_3$): δ_H (ppm) 8.63 (br. s, 3H, NH_3), 7.08 (d, 1H, *J* = 3.6 Hz, *H*-1), 5.47 (dd, 1H, *J* = 10.4, 9.2 Hz, *H*-3), 5.21 (dd, 1H, *J* = 10.1, 9.2 Hz, *H*-4), 4.39–4.22 (m, 2H, *H*-5, *H*-6a), 4.13 (dd, 1H, *J* = 12.4, 1.8 Hz, *H*-6b), 4.01–3.84 (m, 1H, *H*-2), 2.23 (3 x s, 9H, CH_3CO_2). **^{13}C NMR** (101 MHz, $CDCl_3$): δ_C (ppm) 171.7–169.3 (3 x s, CH_3CO_2), 85.5 (s, *C*-1), 73.0 (s, *C*-5), 70.0 (s, *C*-3), 66.8 (s, *C*-4), 60.9 (s, *C*-6), 54.4 (s, *C*-2), 21.9–20.6 (3 x s, CH_3CO_2). The ^{1}H NMR data is in accordance with the literature.[10]

3.1.13 Synthesis of Methyl 2-amino-2-deoxy-3,4,6-tri-*O*-acetyl-β-D-glucopyranose, S5

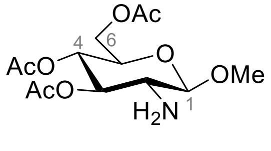

The title compound (1.11 g, 3.47 mmol, 65% as a 1:19 ratio of α:β) was synthesised according to the literature procedure.[10] **^{1}H NMR** (400 MHz, $CDCl_3$): δ_H (ppm) 5.00 (t, 1H, *J* = 9.6 Hz, *H*-4), 4.95 (t, 1H, *J* = 9.6 Hz, *H*-3), 4.28 (dd, 1H, *J* = 12.2, 4.6 Hz, *H*-6a), 4.14 (d, 1H, *J* = 8.0 Hz, *H*-1), 4.10 (dd, 1H, *J* = 12.2, 2.4 Hz, *H*-6b), 3.67 (ddd, 1H, *J* = 9.6, 4.6, 2.4 Hz, *H*-5), 3.53 (s, 3H, OMe), 2.89 (dd, 1H, *J* = 9.6, 8.0 Hz, *H*-2), 2.06–2.00 (3 x s, 9H, CH_3CO_2), 1.50 (s, 2H, NH_2). **^{13}C NMR** (101 MHz, $CDCl_3$): δ_C (ppm) 170.9–169.9 (3 x s, CH_3CO_2), 105.1 (s, *C*-1), 75.5 (s, *C*-3), 72.0 (s, *C*-5), 69.0 (s, *C*-4), 62.3 (s, *C*-6), 57.5 (s, OMe), 56.0 (s, *C*-2), 20.9–20.8 (3 x s, 9H, CH_3CO_2). The spectroscopic data is in accordance with the literature.[11] *The α anomer was observed in the ^{1}H NMR spectrum at ca. 5%, which is removed during the purification of **6**.*

3.1.14 Synthesis of Methyl 2-acrylamido-2-deoxy-3,4,6-tri-*O*-acetyl-β-D-glucopyranose, 6

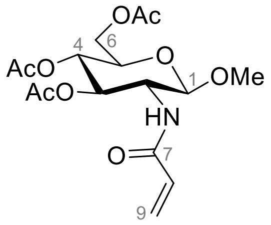

Triethylamine (0.68 mL, 4.70 mmol) was added to a solution of amine **S5** (974 mg, 3.05 mmol) in dry DCM (8.0 mL) and cooled to 0 °C. Acryloyl chloride (0.30 mL, 3.76 mmol) was added dropwise, and the reaction mixture allowed to warm to RT. After stirring for 1 h at RT the volatiles were removed *in vacuo*. The crude product was purified by column chromatography (50–60% EtOAc in pet. ether) to afford the title compound (1.06 g, 2.83 mmol, 93%) as a white solid. **^{1}H NMR** (400 MHz, $CDCl_3$): δ_H (ppm) 6.26 (dd, 1H, *J* = 17.0, 1.4 Hz, *H*-9a), 6.04 (dd, 1H, *J* = 16.9, 10.3 Hz, *H*-8), 5.85 (d, 1H, *J* = 8.9 Hz, N*H*), 5.65 (dd, 1H, *J* = 10.3, 1.3 Hz, *H*-9b), 5.32 (dd, 1H, *J* = 10.6, 9.3 Hz, *H*-3), 5.09 (dd, 1H, *J* = 10.0, 9.3 Hz, *H*-4), 4.62 (d, 1H, *J* = 8.3 Hz, *H*-1), 4.28 (dd, 1H, *J* = 12.3, 4.8 Hz, *H*-6a), 4.15 (dd, *J* = 12.3, 2.5 Hz, *H*-6b), 3.99 (dt, *J* = 10.5, 8.6 Hz, *H*-2), 3.73 (ddd, *J* = 9.9, 4.8, 2.5 Hz, *H*-5), 3.48 (s, 3H, OMe), 2.08–1.99 (3 x s, 9H, CH_3CO_2). **^{13}C NMR** (101 MHz, $CDCl_3$): δ_C (ppm) 171.1–169.6 (3 x s, CH_3CO_2), 165.8 (s, *C*-7), 130.5 (s, *C*-8), 127.5 (s, *C*-9), 101.8 (s, *C*-1), 72.5 (s, *C*-3), 72.0 (s, *C*-5), 68.8 (s, *C*-4), 62.3 (s, *C*-6), 56.9 (s, OMe), 54.6 (s, *C*-2), 20.9–20.6 (m, 3 x CH_3CO_2). **HR-MS** (ESI+): *m/z* calcd. for $C_{16}H_{23}NNaO_9$ ($[M+Na]^+$) = 396.1265; obs. = 396.1273.

3.1.15 Synthesis of Methyl 2-acrylamido-2-deoxy-β-D-glucopyranose, S6

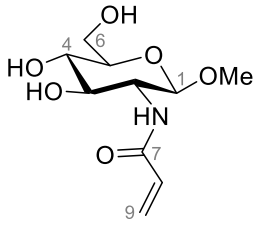

NaOMe in MeOH (0.2 mL, 25 wt% in MeOH) was added to a stirred solution of **6** (0.804 mmol) in MeOH (40 mL). After the reaction had reached completion, as monitored by TLC, the reaction was neutralised by the addition of Amberlite IR 120. The mixture was then filtered and concentrated *in vacuo* to give the title compound (191 mg, 0.773 mmol, 96%) as a white solid. **^{1}H NMR** (400 MHz, DMSO-d_6): δ_H (ppm) 7.95 (d, 1H, *J* = 8.5 Hz, 1H, NH), 6.19 (dd, 1H, *J* = 17.1, 10.0 Hz,

H-8), 6.05 (dd, 1H, *J* = 17.1, 2.4 Hz, *H*-9a), 5.57 (dd, *J* = 10.0, 2.4 Hz, *H*-9b), 5.02 (br. s, 2H, 2 x OH), 4.58 (br. s, 1H, OH), 4.22 (d, *J* = 8.4 Hz, *H*-1), 3.69 (d, 1H, *J* = 11.6 Hz, *H*-6a), 3.56–3.43 (m, 2H, *H*-2, *H*-6b), 3.40–3.26 (m, 1H, *H*-3), 3.31 (s, 3H, OMe), 3.13–3.01 (m, 2H, *H*-4, *H*-5). **^{13}C NMR** (101 MHz, DMSO-d_6): δ_C (ppm) 164.5 (s, *C*-7), 132.4 (s, *C*-8), 124.9 (s, *C*-9), 101.9 (s, *C*-1), 77.1 (s, *C*-5), 74.3 (s, *C*-3), 70.7 (s, *C*-4), 61.1 (s, *C*-6), 55.7 (s, OMe), 55.3 (s, *C*-2). **HR-MS** (ESI+): *m/z* calcd. for $C_{10}H_{17}NNaO_6$ ($[M+Na]^+$) = 270.0948; obs. = 270.0950.

3.1.16 Synthesis of protected $Ph_2PCH_2CH_2P(CH_2CH_2CONH^{C2}Glc)_2$, **L4**

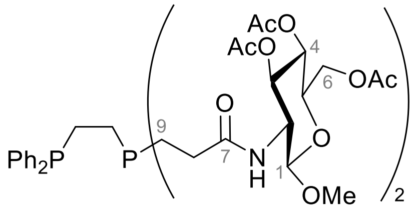

To a solution of $Ph_2PCH_2CH_2PH_2$ (75.0 mg, 0.305 mmol) in dry DCM (0.7 mL) was added [Pt(nbe)$_3$] (7.2 mg, 15.2 µmol) and stirred at room temperature to afford a deep orange solution. After 10 min, ^{i}PrOH (4.06 mmol, 0.9 mL) was added, followed by dropwise addition of acrylamide **6** (228 mg, 0.609 mmol) in DCM (0.6 mL) and the mixture was left to stir at RT until the reaction had reached completion, as monitored by *in situ* ^{31}P{^{1}H} NMR spectroscopy. The crude product was purified by flash column chromatography (2% MeOH in DCM) to afford the product (166 mg, 0.167 µmol, 55%) as a white solid. **^{31}P{^{1}H} NMR** (162 MHz, CDCl$_3$): δ_P (ppm) −12.4 (d, $^3J_{P,P}$ = 28.6 Hz, *P*Ph$_2$), −23.3 (d, $^3J_{P,P}$ = 28.6 Hz, *P*R$_2$). **^{1}H NMR** (500 MHz, CDCl$_3$): δ_H (ppm) 7.43–7.30 (m, 10H, Ar-*H*), 6.51 (t, 2H, *J* = 9.8, NH), 5.27 (ddd, 2H, *J* = 10.2, 9.3, 6.3 Hz, *H*-3), 5.06 (q, 2H, *J* = 10.2 Hz, *H*-4), 4.56 (d, 1H, *J* = 8.3 Hz, *H*-1a), 4.53 (d, 1H, *J* = 8.3 Hz, *H*-1b), 4.28 (ddd, 2H, *J* = 12.3, 4.8, 2.0 Hz, *H*-6a), 4.15 (dd, 2H, *J* = 12.3, 2.5 Hz, *H*-6b), 4.01–3.91 (m, 2H, *H*-2), 3.74 (ddd, 2H, *J* = 10.2, 4.8, 2.5 Hz, *H*-5), 3.48 (s, 6H, OMe), 2.32–1.93 (m, 24H, *H*-8/*H*-9, 6 x C*H*$_3$CO$_2$, C*H*$_2$), 1.73–1.49 (m, 4H, *H*-8/*H*-9), 1.47–1.37 (m, 2H, C*H*$_2$). **^{13}C NMR** (126 MHz, CDCl$_3$): δ_C (ppm) 173.1 (d, $^3J_{P,C}$ = 10.3 Hz, *C*-7a), 172.7 (d, $^3J_{P,C}$ = 6.8 Hz, *C*-7b), 171.6 (2 x s, 2 x CH$_3$*C*O$_2$), 170.9 (s, 2 x CH$_3$*C*O$_2$), 169.5 (s, 2 x CH$_3$*C*O$_2$), 138.1 (dd, $J_{P,C}$ = 13.5, 2.5 Hz, Ar*C*), 132.9, (2 x d, $^2J_{P,C}$ = 18.5 Hz, *o*-Ar*C*H), 128.9 (s, *p*-Ar*C*H), 128.7 (d, $^3J_{P,C}$ = 6.6 Hz, *m*-ArCH), 101.8 (2 x s, *C*-1), 73.0 (2 x s, *C*-3), 71.9 (s, *C*-5), 69.0 (2 x s, *C*-4), 62.3 (s, *C*-6), 56.8 (2 x s, OMe), 54.3 (2 x s, *C*-3), 33.1 (d, *J* = 15.6 Hz, *C*-8/*C*-9a), 32.1 (d, *J* = 11.6 Hz, *C*-8/*C*-9a), 23.9 (t, *J* = 13.7 Hz, *C*H$_2$), 22.4 (t, *J* = 15.0 Hz, *C*H$_2$), 21.9–20.7 (m, *C*-8/*C*-9b, 6 x *C*H$_3$CO$_2$). **HR-MS** (ESI+): *m/z* calcd. for $C_{46}H_{63}N_2O_{18}P_2$ ($[M+H]^+$) = 993.3531; obs. = 993.3551.

3.1.17 Synthesis of $Ph_2PCH_2CH_2P(CH_2CH_2CONH^{C2}Glc)_2$, **L6**

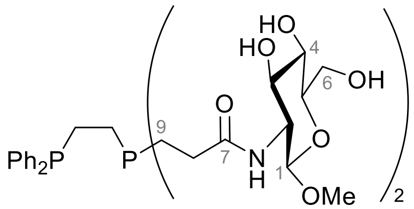

NaOMe in MeOH (36 µL, 25 wt% in MeOH) was added to a stirred solution of **L4** (123 mg, 0.124 mmol) in deoxygenated MeOH (7.2 mL). After the reaction had reached completion, as monitored by TLC-MS, the reaction was neutralised by the addition of Amberlite IR 120. The reaction mixture was then filtered and concentrated *in vacuo* to give the title compound (75.5 mg, 102 µmol, 82%) as a white solid. **^{31}P{^{1}H} NMR** (162 MHz, CD$_3$OD): δ_P (ppm) −12.7 (d, $^3J_{P,P}$ = 29.4 Hz, *P*Ph$_2$), −22.8 (d, $^3J_{P,P}$ = 29.4 Hz, *P*R$_2$). **^{1}H NMR** (500 MHz, DMSO-d_6): δ_H (ppm) 7.66 (d, 2H, *J* = 9.0 Hz, NH), 7.39 (m, 10H, Ar-*H*), 4.98 (br. s, 2H, OH), 4.87 (br. t, 2H, *J* = 5.0 Hz, OH), 4.53 (br. t, 2H, *J* = 6.0 Hz, OH), 4.18 (d, 2H, *J* = 8.4 Hz, *H*-1), 3.68 (dd, 2H, *J* = 11.4, 5.4 Hz, *H*-6a), 3.49–3.36 (m, 4H, *H*-2, *H*-6b), 3.32–3.22 (m, 8H, *H*-3, OMe), 3.11–3.02 (m, 4H, *H*-4, *H*-5), 2.17–2.05 (m, 6H, *H*-8/*H*-9, C*H*$_2$), 1.60 (dd, 4H, *J* = 10.2, 6.7 Hz, *H*-8/*H*-9), 1.44–1.35 (m, 2H, C*H*$_2$). **^{13}C NMR** (126 MHz, DMSO-d_6): δ_C (ppm) 171.8 (d, $^3J_{P,C}$ = 11.6 Hz, *C*-7), 138.2 (dd, $^1J_{P,C}$ = 14.7 Hz, $^3J_{P,C}$ = 3.0 Hz, Ar*C*),

132.4 (d, $^2J_{P,C}$ = 18.3 Hz, *o*-Ar*C*H), 128.8 (s, *p*-Ar*C*H), 128.6 (d, $^3J_{P,C}$ = 6.6 Hz, *m*-Ar*C*H), 101.9 (2 x s, *C*-1), 77.0 (s, *C*-5), 74.4 (2 x s, *C*-3), 70.6 (s, *C*-4), 61.1 (s, *C*-6), 55.7 (s, *C*-2), 55.3 (s, OMe), 31.9 (d, *J* = 14.3 Hz, CH_2), 22.6 (t, $^1J_{P,C}$ = 13.2 Hz, *C*-8/*C*-9), 21.7 (t, $^2J_{P,C}$ = 15.2 Hz, *C*-8/*C*-9), 21.4 (dd, *J* = 13.9, 2.8 Hz, CH_2). **HR-MS** (ESI+): *m/z* calcd. for $C_{34}H_{50}N_2O_{12}P_2Na$ ([*M*+Na]$^+$) = 763.2737; obs. = 763.2751. **$[\alpha]_D^{23.9}$** −17.2 (c = 0.81, MeOH).

Route B, utilising unprotected glucose acrylamide, **S6**:

To a solution of $Ph_2PCH_2CH_2PH_2$ (20.0 mg, 81.2 µmol) in aqueous ^{i}PrOH (0–20%, 0.7 mL) was added [Pt(nbe)$_3$] (1.9 mg, 3.99 µmol) and stirred at room temperature to afford a deep orange solution. After 30 min, acrylamide **S6** (228 mg, 0.609 mmol) was added, and the suspension was left to stir at RT overnight. Dry Et_2O (4 mL) was added, the supernatant removed, and the precipitate washed with Et_2O (2 x 4 mL). The crude product was purified by reverse-phase HPLC (5% solvent B for 5 min, then gradient to 95% solvent B for 30 min) to give **L6** in *ca.* 92% purity by $^{31}P\{^1H\}$ NMR spectroscopy, containing 8% mono-oxide. Optimisation of the purification conditions is ongoing.

3.1.18 Synthesis of [Re(O)$_2$(**L6**)$_2$]I, **8c** and **8t**

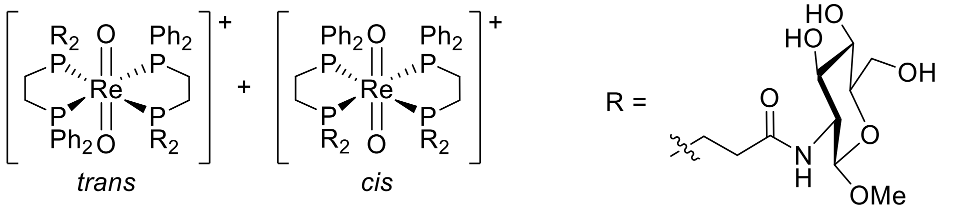

To a solution of [ReI(O)$_2$(PPh$_3$)$_2$] (17.6 mg, 20.3 µmol) in dry MeOH (2.0 mL) was added a solution of **L6** (30.0 mg, 40.5 µmol) in dry MeOH (1.5 mL). The solution turned from violet to clear yellow and was left to stir at RT. Once the complexation had reached completion, as monitored by $^{31}P\{^1H\}$ NMR spectroscopy, the solution was concentrated to dryness and the resulting pale brown precipitate was washed sequentially with diethyl ether (3 x 1 mL). The mixture of *cis* and *trans* isomers (35.5 mg, 19.4 µmol, 96%) was isolated as an off-white solid. **$^{31}P\{^1H\}$ NMR** (162 MHz, CD_3OD): δ_P (ppm) 16.1–7.3 (m). **HR-MS** (Nanospray): *m/z* calcd. for $C_{68}H_{100}N_4O_{26}P_4{}^{187}Re$ ([*M*]$^+$) = 1699.5134; obs. = 1699.5178.

4 RADIOLABELLING PROCEDURES

4.1.1 Kit preparation:

An aqueous stock solution was prepared containing the required amounts of sodium bicarbonate, tin chloride and sodium tartrate or sodium gluconate. Aliquots of the stock solution were mixed with the required amount of **L1**, **L2-Na$_2$**, **L5** or **L6** to form the kit solutions outlined in Table S1, which were immediately frozen and lyophilised using a freeze dryer. The lyophilised kits were stored in a freezer prior to use.

Table S1: Lyophilised kit formulations **L1**, **L2-Na$_2$, L5** and **L6** for radiolabelling.

	**L1 kit**	**L2 kit**	**L5 kit**	**L6 kit**
**Ligand**	0.65 μmol<br>0.27 mg	0.65 μmol<br>0.28 mg	0.65 μmol<br>0.46 mg	0.65 μmol<br>0.48 mg
**$SnCl_2.2H_2O$**	0.26 μmol<br>0.06 mg	0.26 μmol<br>0.06 mg	0.26 μmol<br>0.06 mg	0.26 μmol<br>0.06 mg
**Sodium tartrate**	3.60 μmol<br>1.00 mg	-	-	-
**Sodium gluconate**	-	4.60 μmol<br>1.00 mg	4.60 μmol<br>1.00 mg	4.60 μmol<br>1.00 mg
**$NaHCO_3$**	20.7 μmol<br>1.74 mg	20.7 μmol<br>1.74 mg	20.7 μmol<br>1.74 mg	20.7 μmol<br>1.74 mg

4.1.2 Radiolabelling with $^{99m}TcO_4^-$:

L1 and **L2-Na$_2$** were radiolabelled by simple addition of generator-produced $^{99m}TcO_4^-$ (50–60 MBq) in saline solution (300 μL, 0.9% NaCl in water, w/v), using the lyophilised kits described in Table S1. The radiolabelling reaction mixtures were left to react at either 60 °C (**L1**) or ambient temperature (**L2-Na$_2$**) for 30 min. Aliquots were analysed by analytical HPLC to determine radiochemical yields.

L5 and **L6** were radiolabelled by simple addition of generator-produced $^{99m}TcO_4^-$ (220–280 MBq) in saline solution (0.9% NaCl in water, w/v), using the lyophilised kits described in Table S1. The radiolabelling reaction mixtures were left to react at ambient temperature (20–23 °C) for 5 min. Aliquots were analysed by analytical HPLC to determine radiochemical yields.

HPLC was carried out on an Agilent 1200 HPLC system with ultraviolet (UV) spectroscopic detection at 254 nm. For radioactivity detection, the HPLC was attached to either a Raytest Gabi detector coupled with a sodium iodide probe, or a LabLogic Flow-Count detector coupled to a sodium iodide probe. Analytical HPLC was acquired using a reverse-phase analytical (4.6 x 150 mm, 5 µm) Agilent Zorbax Eclipse XDB-C18 column, a flow rate of 1 mL min^{-1}, and the following linear gradient: 100% A/0% B to 0% A/100% B over 30 min, A = water with 0.1% trifluoroacetic acid, B = acetonitrile with 0.1% trifluoroacetic acid.

For serum stability and *in vivo* studies using [^{99m}Tc(O)$_2$(**L5**)$_2$]$^+$ (**9c** and **9t**), these kit-based reaction solutions were further purified. Solutions of **9c** and **9t** were separated using reverse-phase HPLC, with each of *cis*-[^{99m}Tc(O)$_2$(**L5**)$_2$]$^+$ (**9c**) and *trans*-[^{99m}Tc(O)$_2$(**L5**)$_2$]$^+$ (**9t**) separated from other reaction components, including free **L5** chelator. Fractions containing either **9c** or **9t** were isolated, volatiles were removed, and the radiotracers reconstituted in either aqueous saline or PBS solution.

4.1.3 Radiolabelling of L5 and L6 with $^{188}ReO_4^-$:

Solutions of $^{188}ReO_4^-$ in saline solution were obtained from an Oncobeta ^{188}W/^{188}Re generator (Munich, Germany). Aqueous saline solution containing ^{188}Re (250 μL, 25 MBq) was added to an aqueous solution of sodium citrate (1 M, 100 μL) and stannous chloride (7 mg), and heated at 90 °C for 30 min. An aliquot of this solution (50 μL, 5 MBq) was then added to an aqueous solution of either **L5** or **L6** (0.5 mg in 50 μL deionised water), and either left to react for 5 min at ambient temperature, or heated at 90 °C for 30 min. Aliquots were analysed by analytical HPLC to determine radiochemical yields, using the same HPLC method described above.

4.1.4 Serum stability of *cis* and *trans*-[$^{99m}Tc(O)_2$(**L5**)$_2$]$^+$ (**9c** and **9t**):

A solution containing either *cis* or *trans*-[$^{99m}Tc(O)_2$(**L5**)$_2$]$^+$ (**9c** or **9t**) (20 µL, 2–30 MBq) was added to filtered human serum (100 µL) and incubated at 37 ˚C. At either 2 or 24 h, each sample (was treated with ice-cold acetonitrile (200 µL) to precipitate and remove serum proteins. Acetonitrile in the supernatant was then removed by evaporation under a stream of N_2 gas (40 ˚C, 30 min). The final solution was then analysed by reverse-phase analytical HPLC.

4.1.5 *In vivo* imaging of *cis* and *trans*-[$^{99m}Tc(O)_2$(**L5**)$_2$]$^+$ (**9c** and **9t**) in healthy mice:

Animal imaging studies were ethically reviewed and carried out in accordance with the Animals (Scientific Procedures) Act 1986 (ASPA) UK Home Office regulations governing animal experimentation. Mice were purchased from Charles River (Margate, UK). Mice were fasted for 12–14 h prior to tracer administration and imaging. A female Balb/c mouse (8–10 weeks old, n = 1) was anaesthetised (2.5% v/v isofluorane, 0.8-1.0 L/min O_2 flow rate) and injected intravenously *via* the tail vein with *cis*-[$^{99m}Tc(O)_2$(**L5**)$_2$]$^+$ (**9c**; 7 MBq, >99% RCP) or *trans*-[$^{99m}Tc(O)_2$(**L5**)$_2$]$^+$ (**9t**; 12 MBq, >99% RCP), followed by SPECT/CT scanning. SPECT/CT imaging was accomplished using a pre-clinical nanoScan SPECT/CT Silver Upgrade instrument (Mediso), calibrated for technetium-99m. The SPECT scans were acquired by helical SPECT (4-head scanner with 4 × 9 pinhole collimators), and CT scans by helical CT (55 kVP X-ray source, 1000 ms exposure time in 180 projections over 9 min). 1.0 mm pinhole collimators were used. SPECT acquisition was done in segments: the first segment was acquired at 0–15 min post-injection, and the second segment was acquired 15–30 min post injection (frame time of 12 s; 9 min acquisition time). This was followed by three additional imaging segments of 30 min each (frame time of 33 s; 24.75 min acquisition time) up until 2 h post-injection. At the end of the imaging procedure, the mouse was culled by cervical dislocation and a sample of the urine analysed by analytical HPLC. SPECT images were reconstructed at 0.3mm isotropic voxel size with the HiSPECT (Scivis GmbH) reconstruction software package using standard reconstruction with 35% smoothing and 9 iterations. The CT and SPECT images were further processed and analysed using VivoQuant software (inviCRO, USA).

4.1.6 Biodistribution of *cis* and *trans*-[$^{99m}Tc(O)_2$(**L5**)$_2$]$^+$ (**9c** and **9t**) in healthy mice:

Female Balb/c mice (8–10 weeks old) were fasted for 12–14 h, prior to being weighed, anaesthetised (2.0-2.5% v/v isofluorane, 1.0-1.0-1.5 L/min O_2 flow rate) and injected with *cis*-[$^{99m}Tc(O)_2$(**L5**)$_2$]$^+$ (**9c**; 2 MBq, >99% RCP, n = 4) or *trans*-[$^{99m}Tc(O)_2$(**L5**)$_2$]$^+$ (**9t**; 3 MBq, >99% RCP, n = 4), by intravenous tail vein injection. The mice were kept under anaesthesia until they were culled by cervical dislocation 30 min post-injection. The biodistribution of the tracer was assessed by dissecting, weighing and gamma counting organs/tissues, alongside standard solutions of known ^{99m}Tc radioactivity. The radioactivity measured for each organ/tissue was normalised to obtain values of percentage injected dose per gram (%ID/g).

5 CRYSTALLOGRAPHIC DETAILS

X-ray diffraction experiments on *trans*-[Re(O)$_2$(**L1**)$_2$]I (**1t**) were carried out at 100(2) K on a Bruker APEX II CCD diffractometer using Mo-K$_\alpha$ radiation (λ = 0.71073 Å). Intensities were integrated in SAINT[12] and absorption corrections based on equivalent reflections were applied using SADABS.[13] The structure was solved using Superflip,[14,15] and the structure was refined by full matrix least squares against F^2 in ShelXL[16,17] using Olex2.[18] All of the non-hydrogen atoms were refined anisotropically. While all of the hydrogen atoms were located geometrically and refined using a riding model. The phenyl rings were modelled as disordered over two positions, the occupancies were linked to a free variable with their sum set to equal 1 and refined, prior to being fixed at the refined values. The I^- counterion and methanol solvent were disordered over an inversion centre, on either side of this the occupancy was necessarily 50%, the methanol was further disordered over two positions. The occupancy of the methanol was linked to a free variable and refined with the sum of the occupancies set to equal 0.5, these were subsequently fixed at 0.3:0.2. Restraints were applied to the C–O distance in the methanol and the anisotropic displacement parameters of the disordered atoms to maintain chemically sensible values. Crystal structure and refinement data are given in Table S2.

Crystallographic data for *trans*-[Re(O)$_2$(**L1**)$_2$]I (**1t**) has been deposited with the Cambridge Crystallographic Data Centre as supplementary publication CCDC 2160396. Copies of the data can be obtained free of charge on application to CCDC, 12 Union Road, Cambridge CB2 1EZ, UK [fax(+44) 1223 336033, e-mail: deposit@ccdc.cam.ac.uk].

Table S2 Crystal data and structure refinement for $[Re(O)_2(\mathbf{L1})_2]I$, **1t**

Empirical formula	$C_{45}H_{60}IO_{11}P_4Re$
Formula weight	1213.91
Temperature / K	100(2)
Crystal system	monoclinic
Space group	$P2_1/n$
a / Å	9.6017(8)
b / Å	13.9749(19)
c / Å	18.5824(19)
α / °	90
β / °	100.025(5)
γ / °	90
Volume / Å^3	2455.4(5)
Z	2
ρ_{calc}g / cm^3	1.642
μ / mm^{-1}	3.289
F(000)	1212.0
Crystal size / mm^3	0.254 × 0.132 × 0.132
Radiation	MoKα (λ = 0.71073)
2θ range for data collection / °	3.668 to 52.738
Index ranges	-12 ≤ h ≤ 11, -17 ≤ k ≤ 16, -23 ≤ l ≤ 18
Reflections collected	14576
Independent reflections	5007 [R_{int} = 0.0718, R_{sigma} = 0.0831]
Data/restraints/parameters	5007/398/378
Goodness-of-fit on F^2	0.998
Final R indexes [I>=2σ (I)]	R_1 = 0.0426, wR_2 = 0.0755
Final R indexes [all data]	R_1 = 0.0831, wR_2 = 0.0866
Largest diff. peak/hole / e Å^{-3}	1.25/-1.06

6 SELECTED NMR SPECTRA

$^{31}P\{^1H\}$ NMR

$[Re(O)_2(\mathbf{L1})_2]^+$ **1c** and **1t**

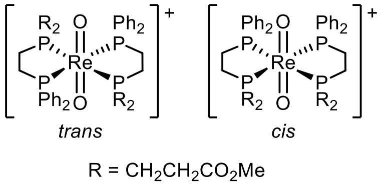

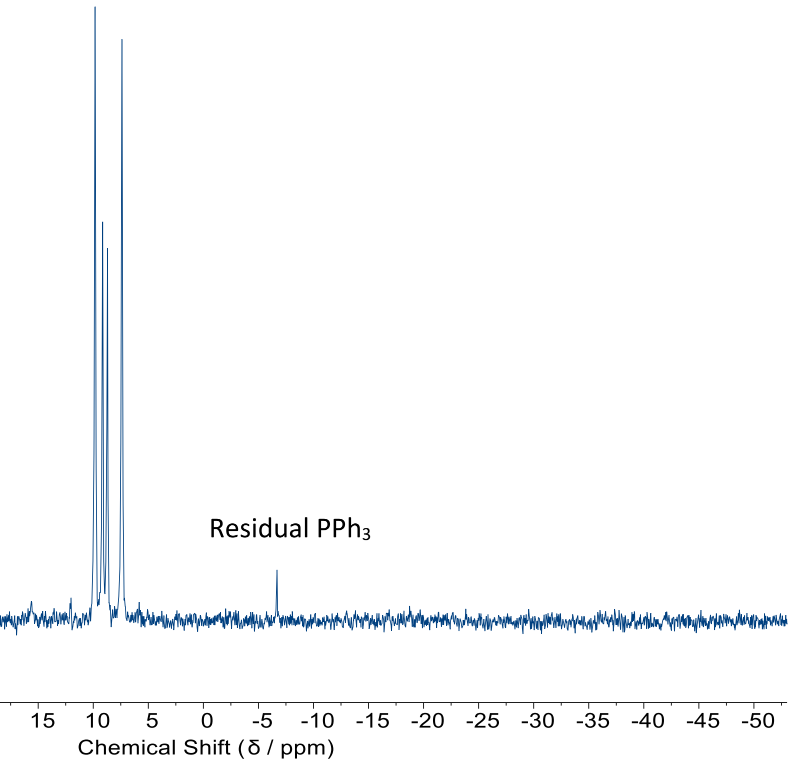

$^{31}P\{^1H\}$ NMR

L2

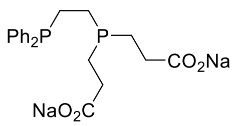

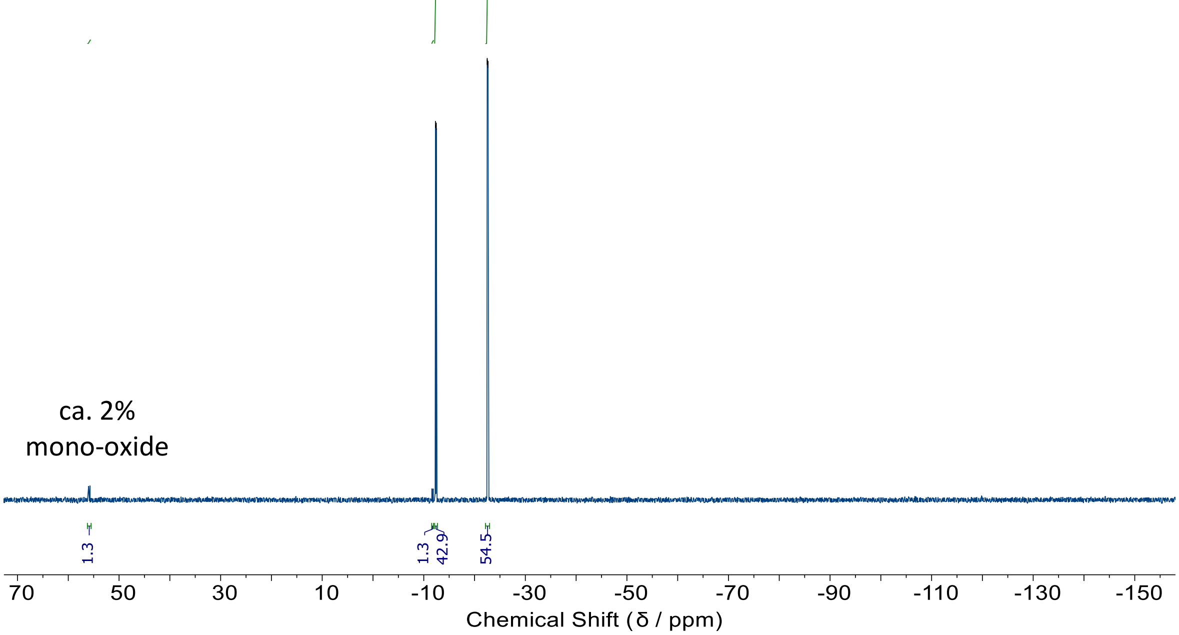

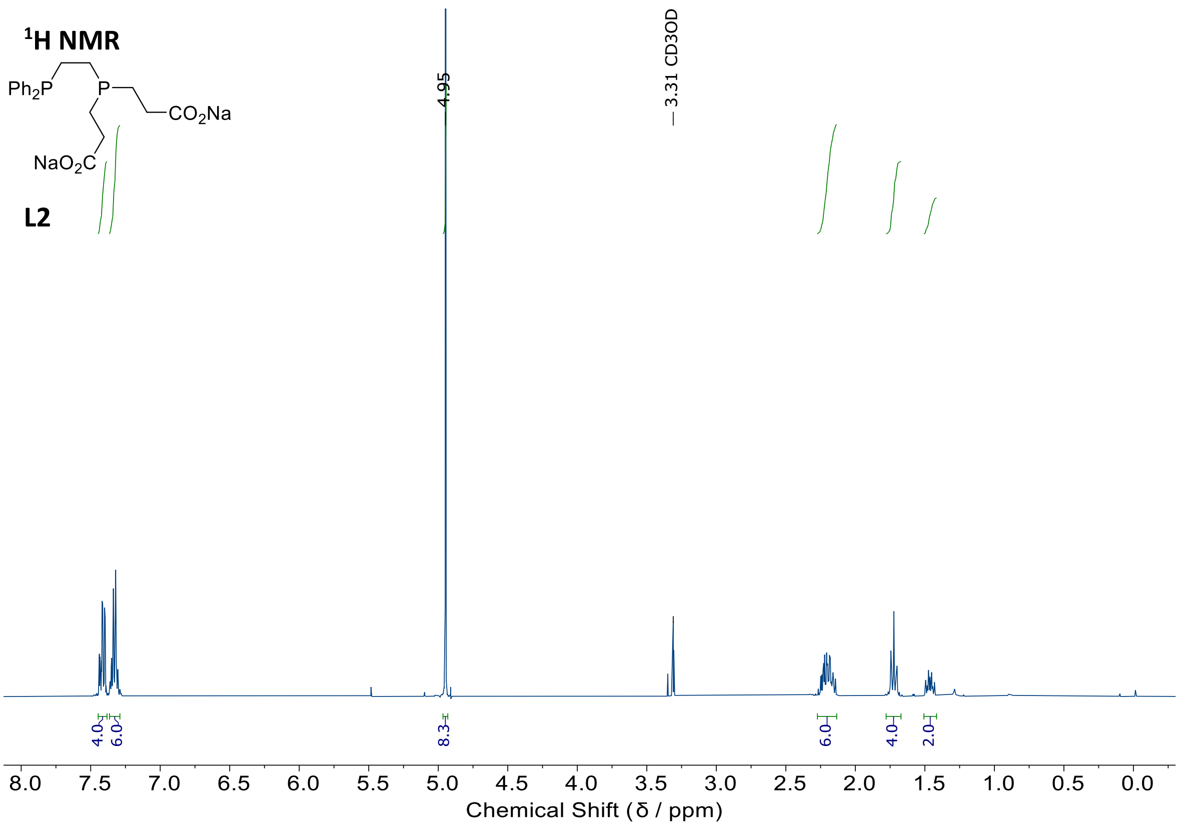

¹H NMR
Ph2P
P
CO2Na
NaO2C
L2
4.95
3.31 CD3OD
4.0
6.0
8.3
6.0
4.0
2.0
8.0 7.5 7.0 6.5 6.0 5.5 5.0 4.5 4.0 3.5 3.0 2.5 2.0 1.5 1.0 0.5 0.0
Chemical Shift (δ / ppm)

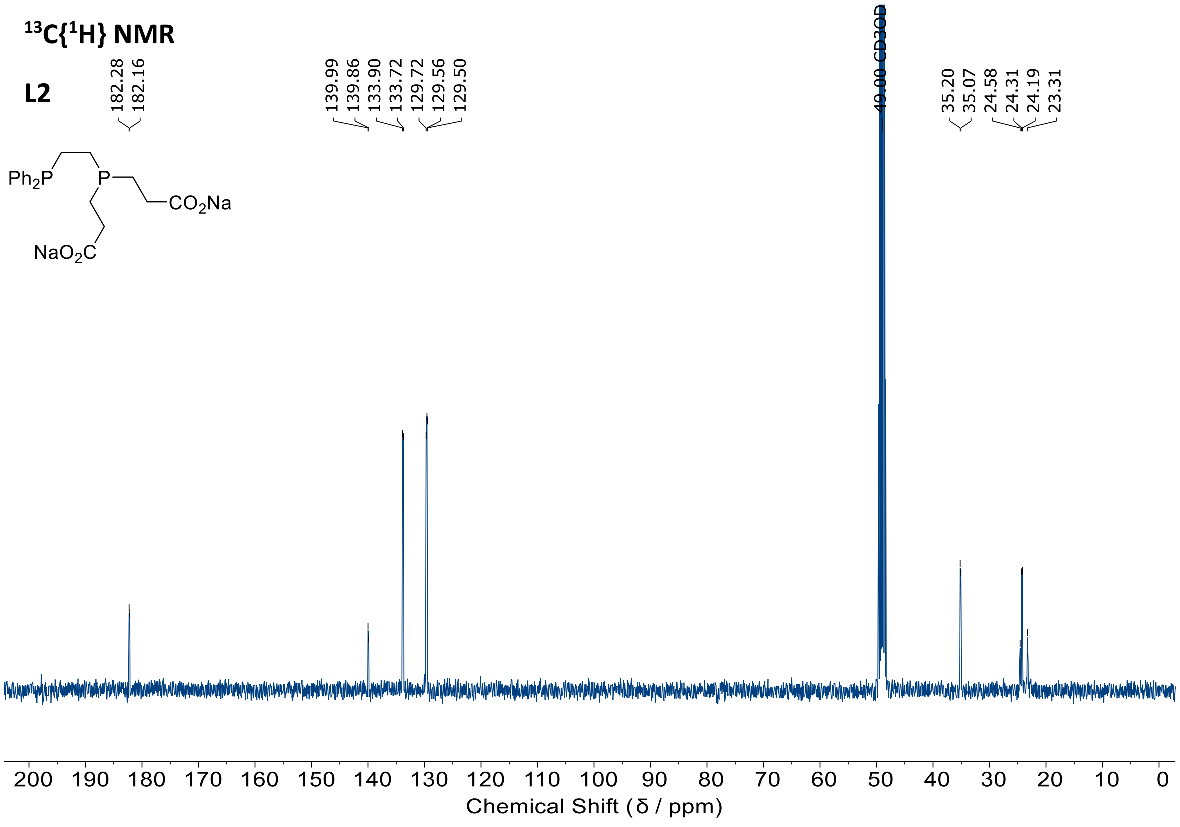

¹³C{¹H} NMR
L2
182.28
182.16
139.99
139.86
133.90
133.72
129.72
129.56
129.50
49.00 CD3OD
35.20
35.07
24.58
24.31
24.19
23.31
Ph2P
P
CO2Na
NaO2C
200 190 180 170 160 150 140 130 120 110 100 90 80 70 60 50 40 30 20 10 0
Chemical Shift (δ / ppm)

$^{31}P\{^1H\}$ NMR

trans-$[Re(O)_2($**L2-H$_2$**$)_2]^+$ **2t**

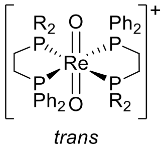

R = $CH_2CH_2CO_2H$

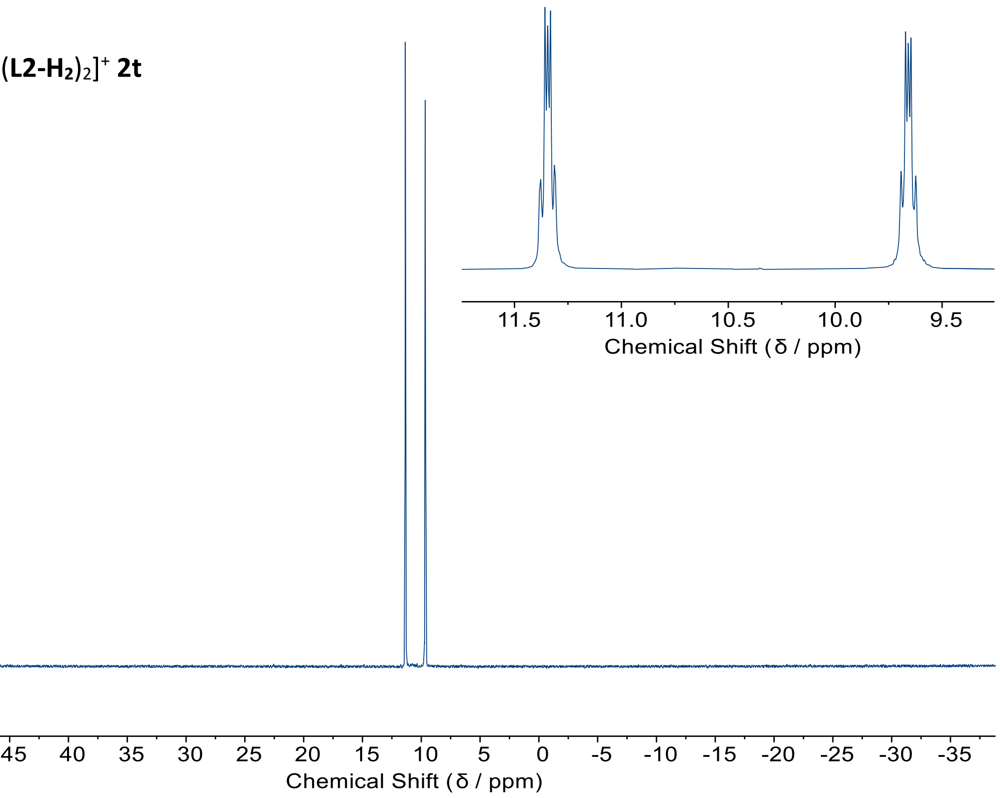

^1H NMR

trans-$[Re(O)_2($**L2-H$_2$**$)_2]^+$ **2t**

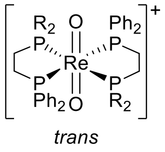

R = $CH_2CH_2CO_2H$

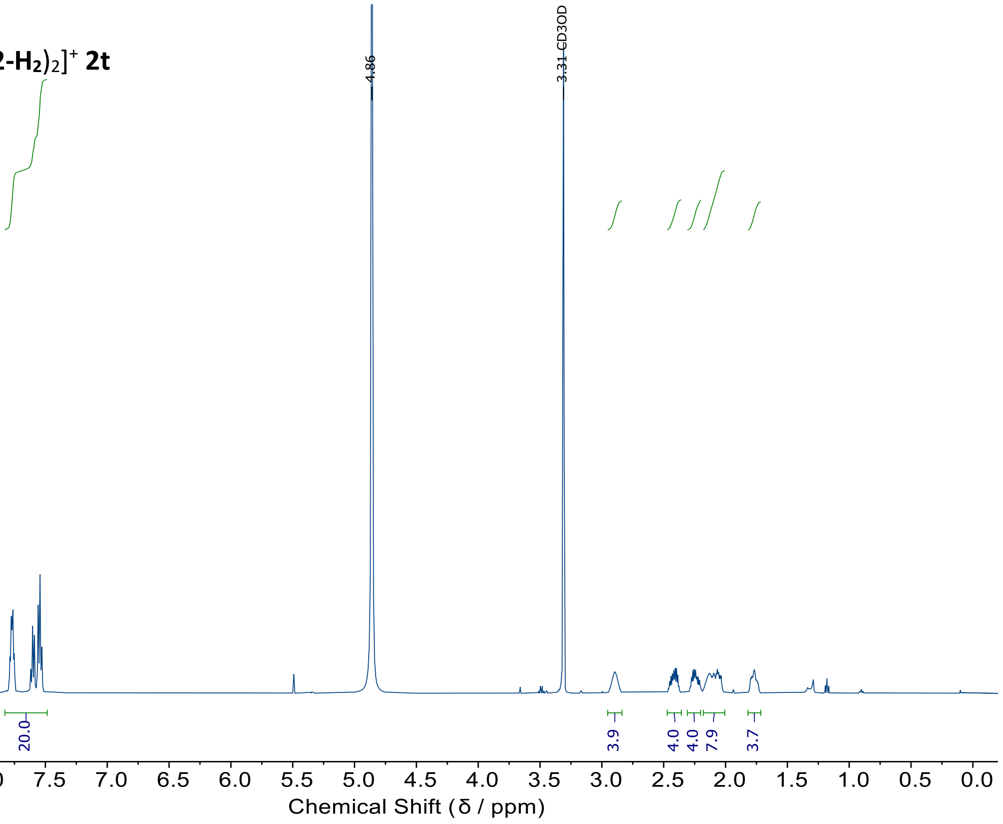

$^{13}C\{^1H\}$ NMR

trans-[Re(O)$_2$(**L2-H$_2$**)$_2$]$^+$ **2t**

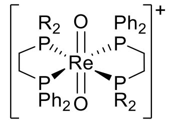

trans

R = $CH_2CH_2CO_2H$

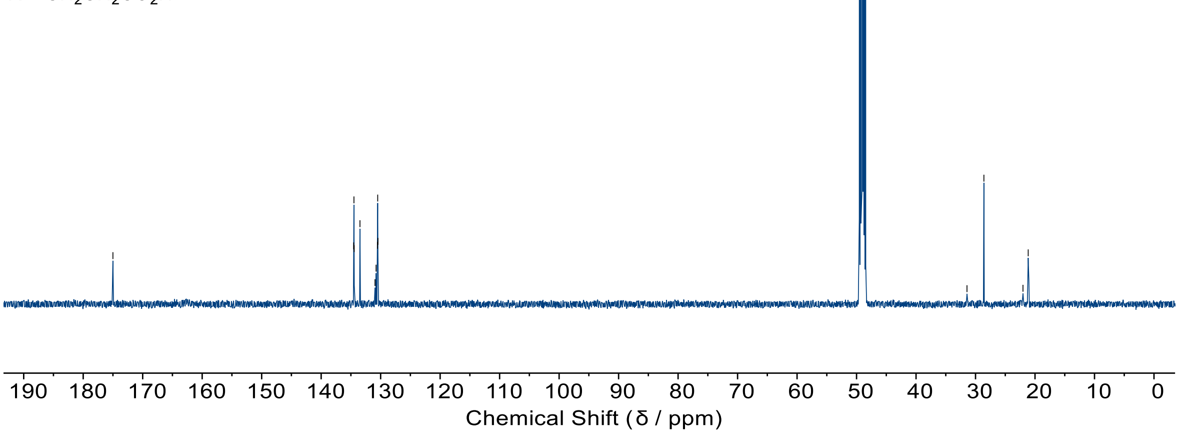

$^{31}P\{^1H\}$ NMR

cis-[Re(O)$_2$(**L2-H$_2$**)$_2$]$^+$ **2c**

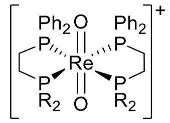

cis

R = $CH_2CH_2CO_2H$

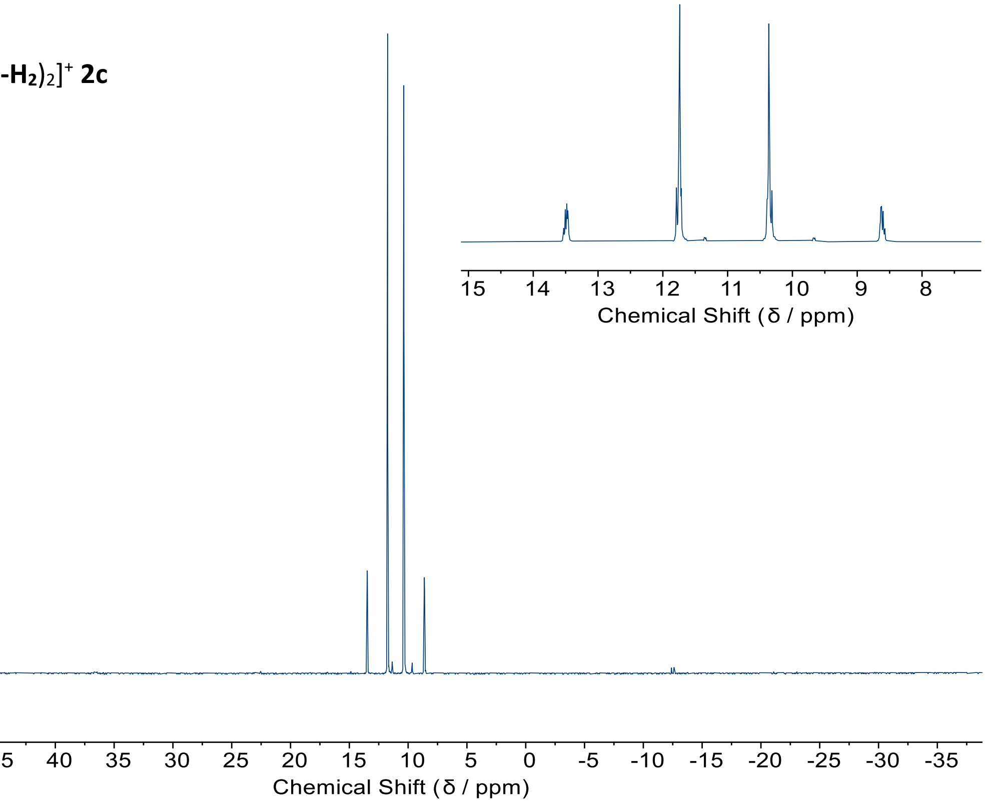

^{1}H NMR

cis-[Re(O)$_2$(**L2-H$_2$**)$_2$]$^+$ **2c**

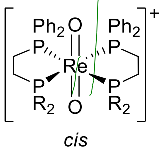

R = $CH_2CH_2CO_2H$

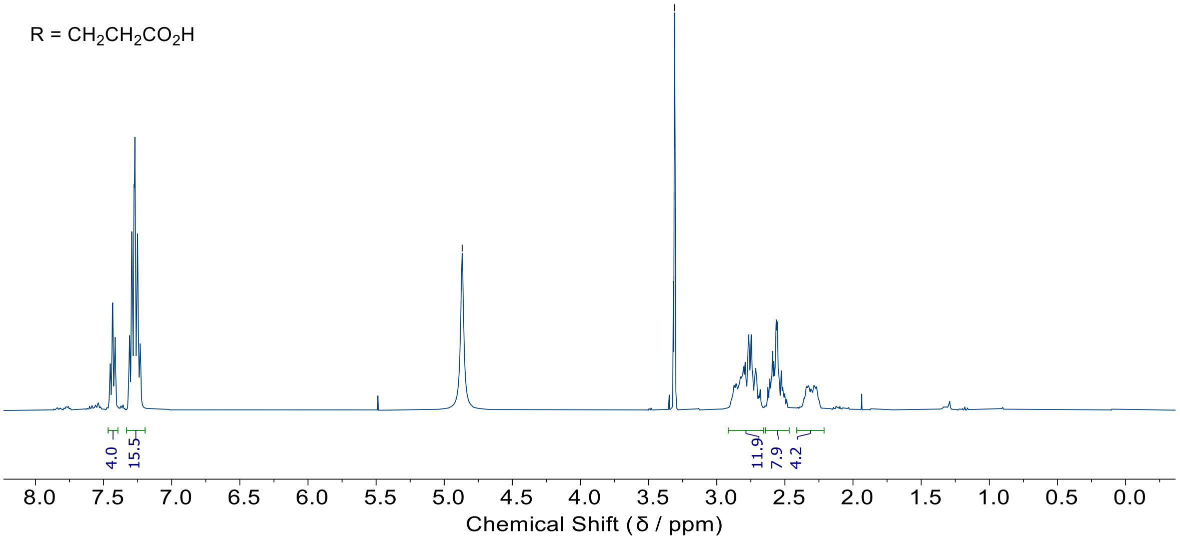

$^{13}C\{^{1}H\}$ NMR

cis-[Re(O)$_2$(**L2-H$_2$**)$_2$]$^+$ **2c**

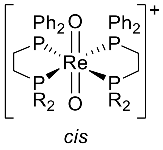

R = $CH_2CH_2CO_2H$

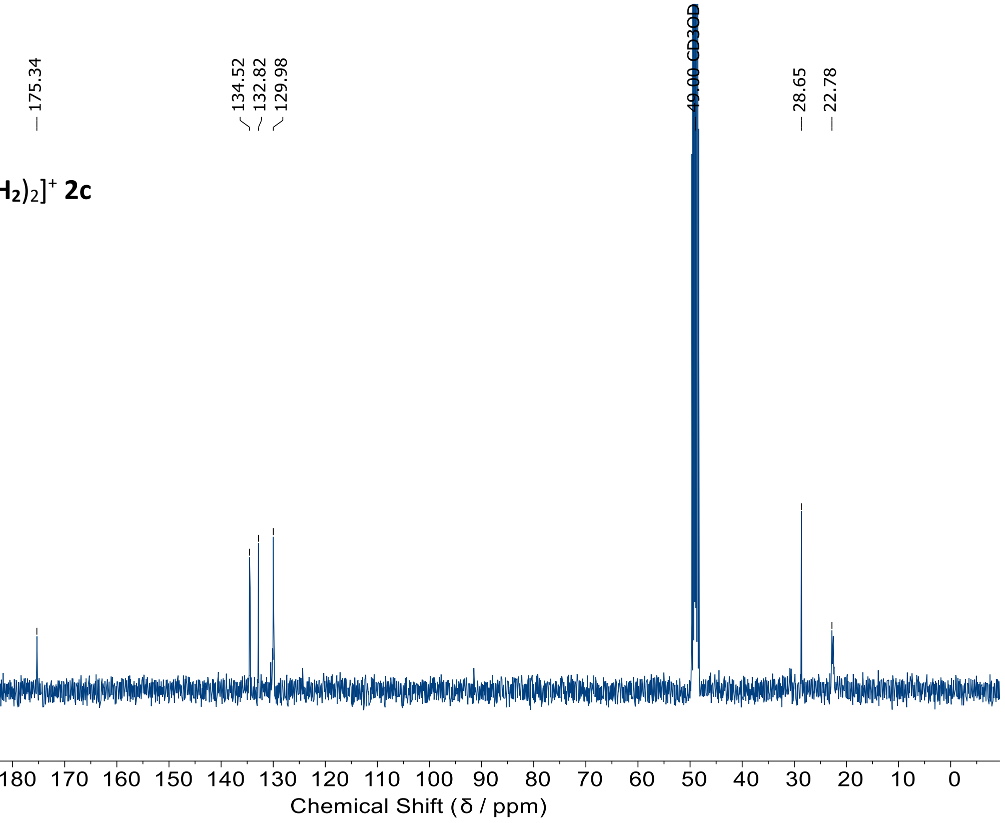

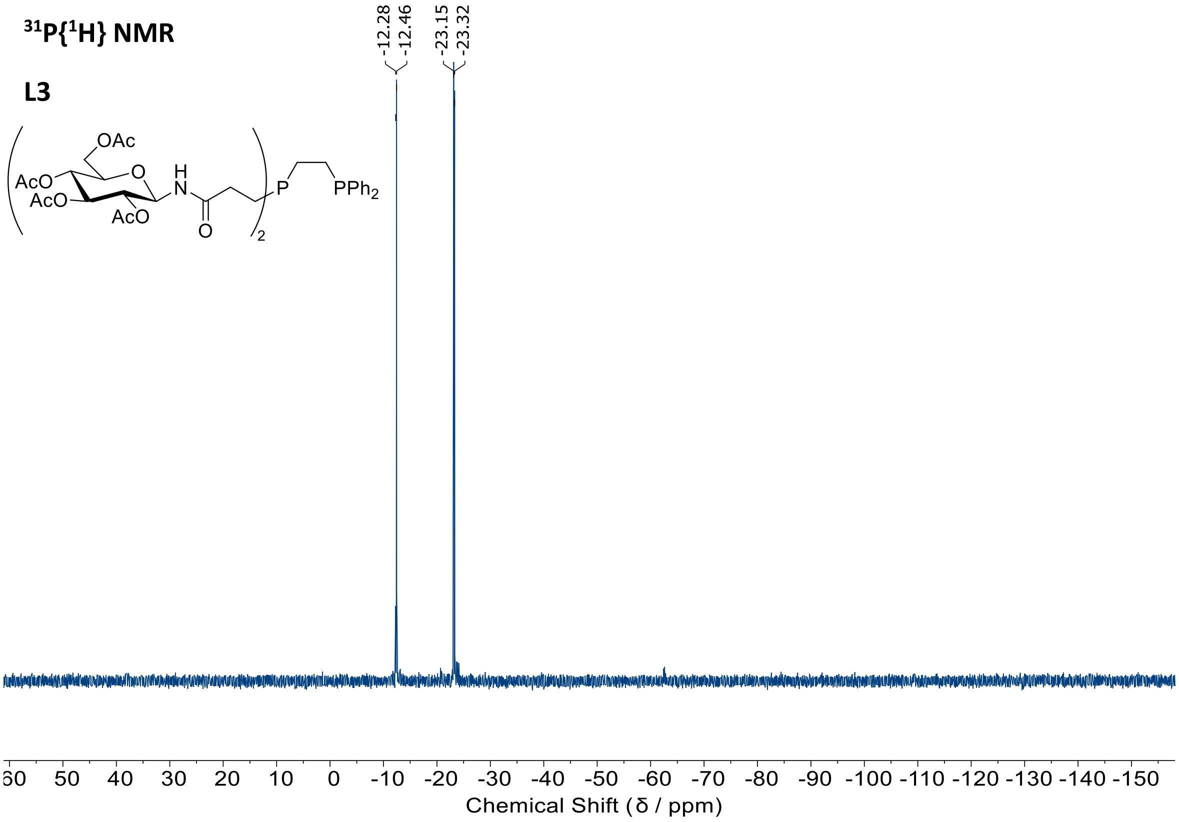
$^{31}P\{^{1}H\}$ NMR
L3
OAc
AcO
AcO
AcO
O
H
N
O
P
PPh2
2
-12.28
-12.46
-23.15
-23.32
60 50 40 30 20 10 0 -10 -20 -30 -40 -50 -60 -70 -80 -90 -100 -110 -120 -130 -140 -150
Chemical Shift (δ / ppm)

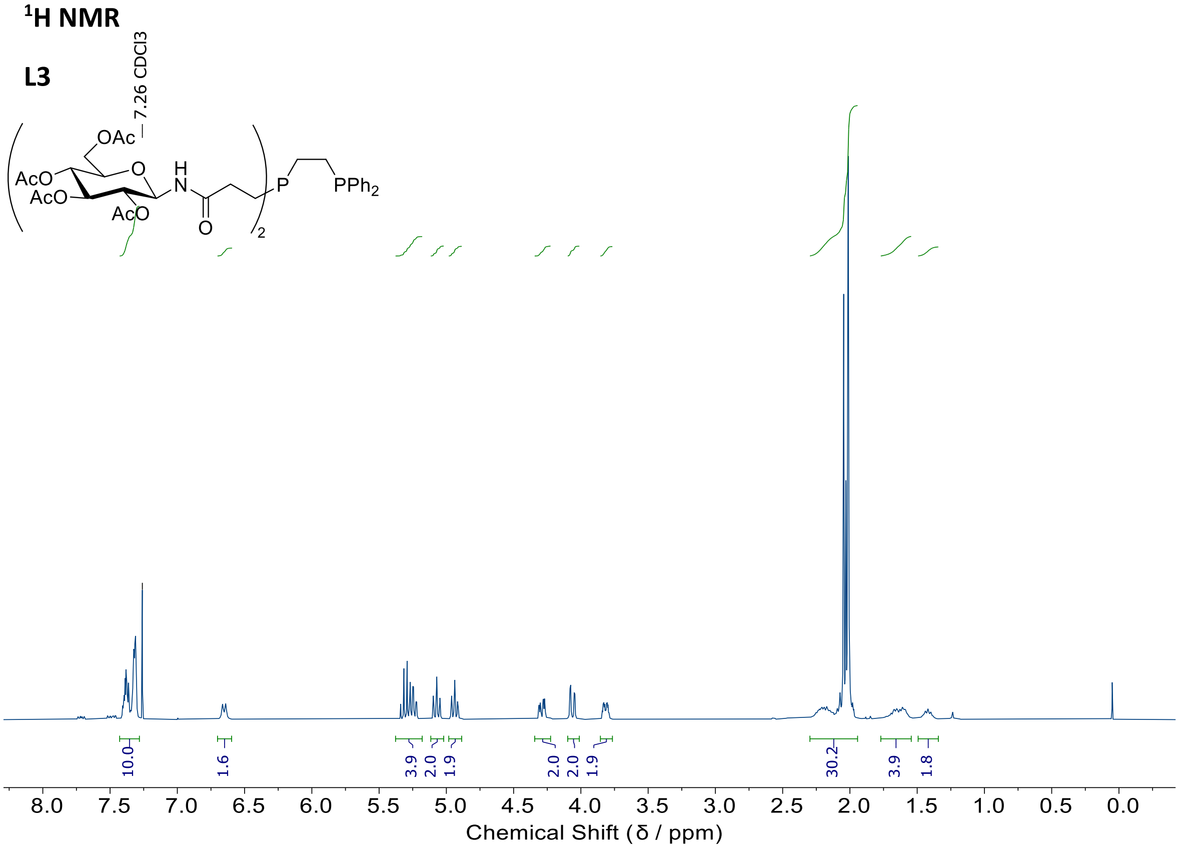
^{1}H NMR
L3
7.26 CDCl3
OAc
AcO
AcO
AcO
O
H
N
O
P
PPh2
2
10.0
1.6
3.9
2.0
1.9
2.0
2.0
1.9
30.2
3.9
1.8
8.0 7.5 7.0 6.5 6.0 5.5 5.0 4.5 4.0 3.5 3.0 2.5 2.0 1.5 1.0 0.5 0.0
Chemical Shift (δ / ppm)

$^{13}C\{^1H\}$ NMR

L3

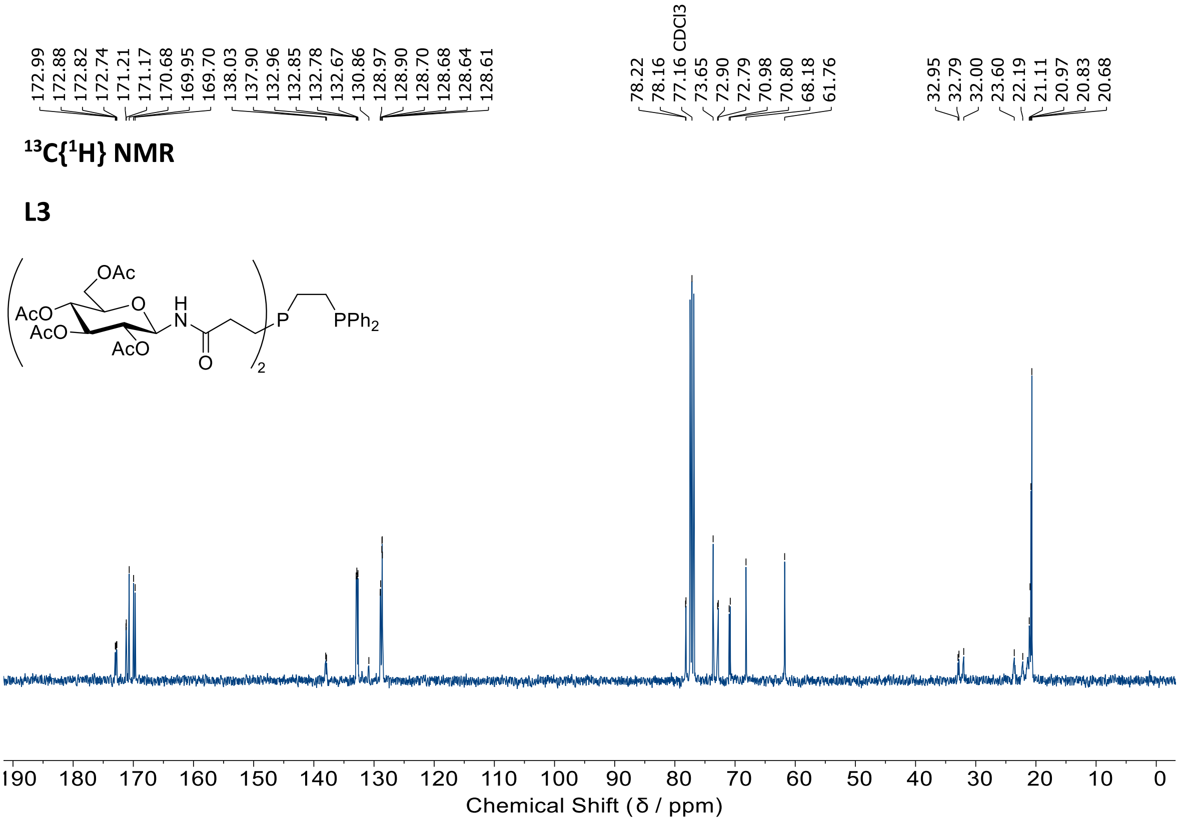

$^{31}P\{^1H\}$ NMR

L5

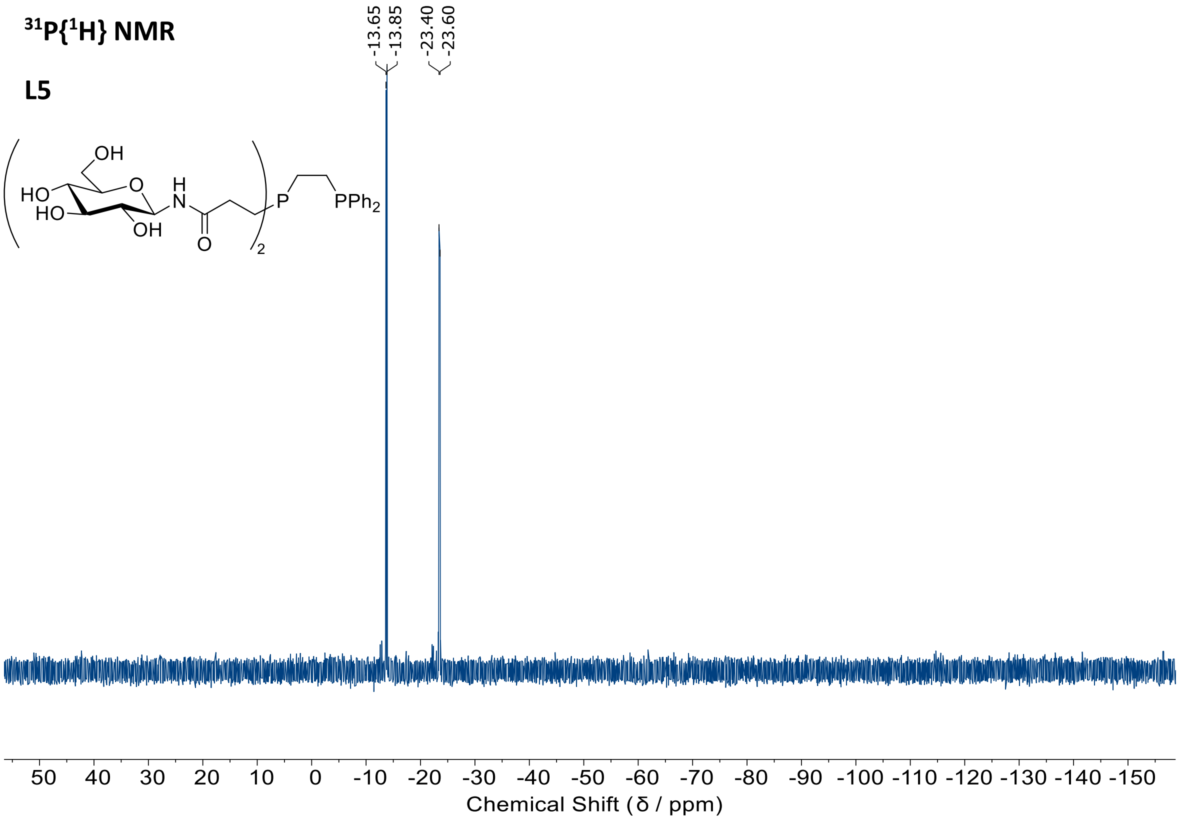

^{1}H NMR

L5

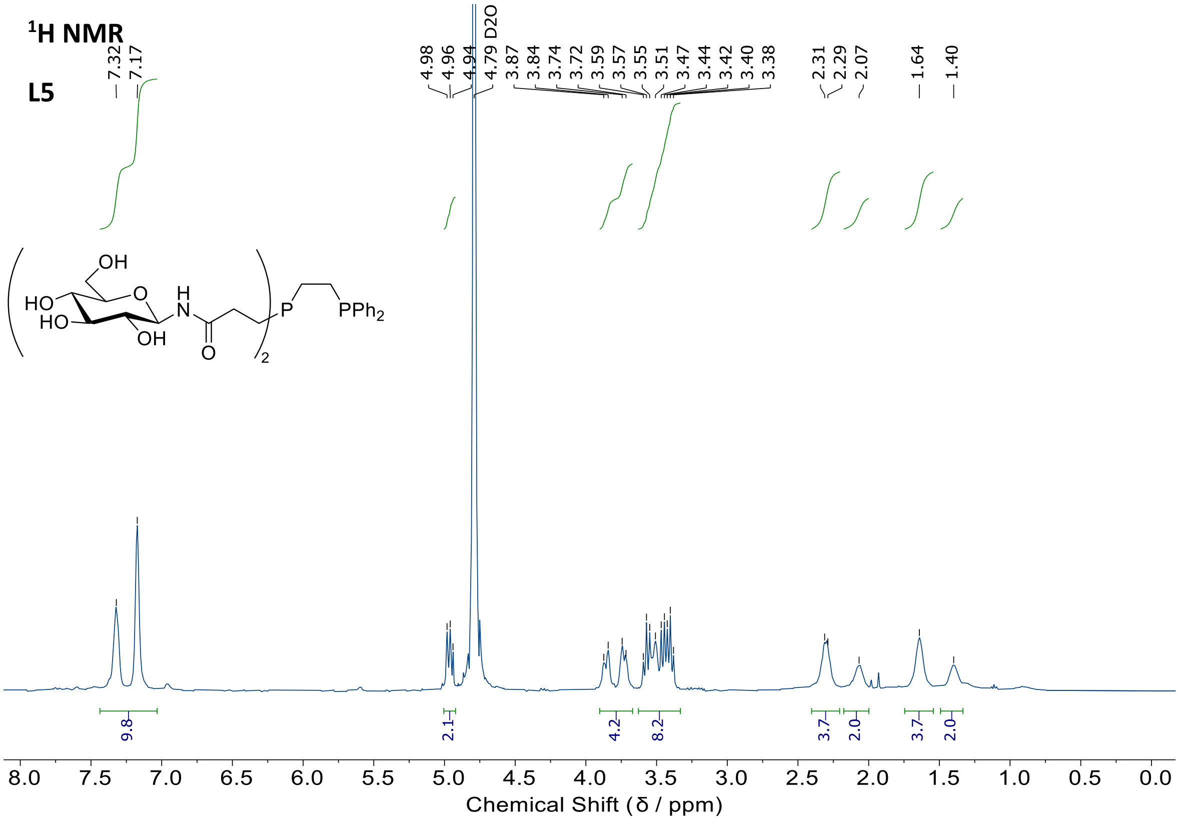

^{13}C{^{1}H} NMR

L5

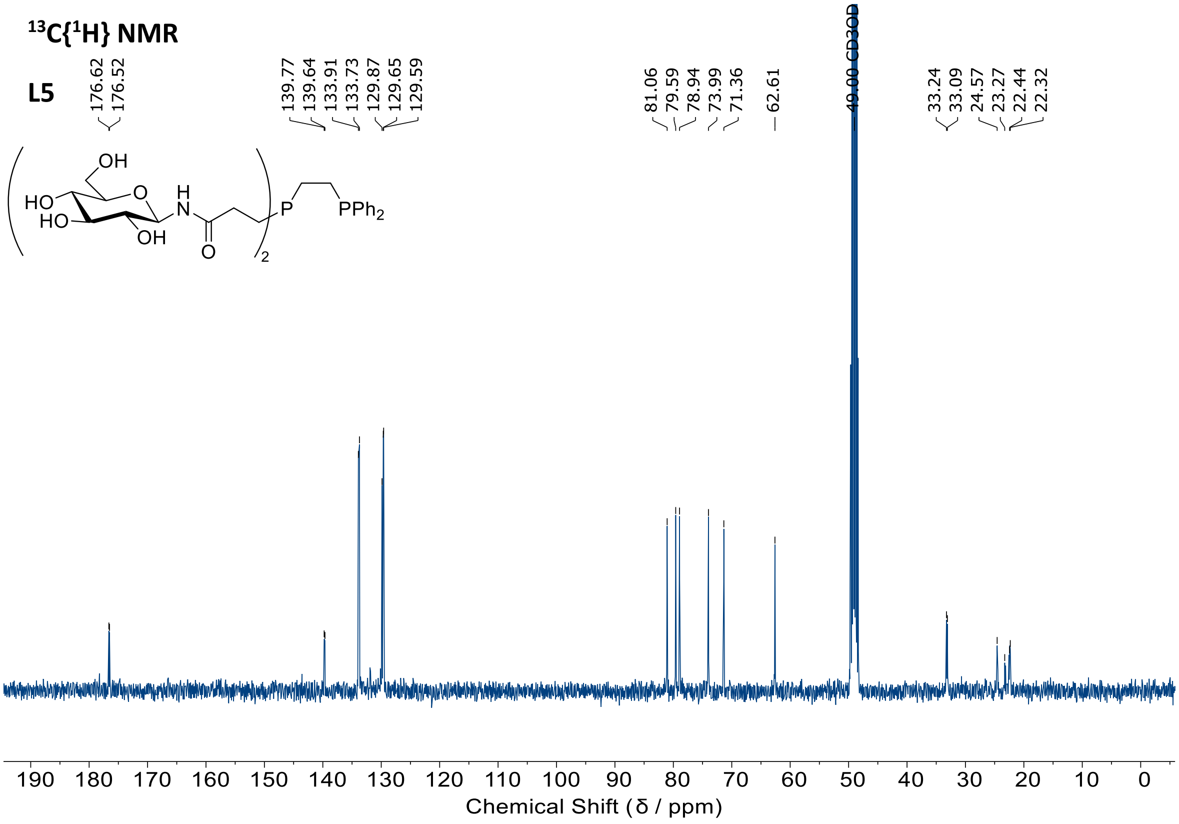

$^{31}P\{^{1}H\}$ NMR

trans-[Re(O)$_2$(**L5**)$_2$]$^+$ **7t**

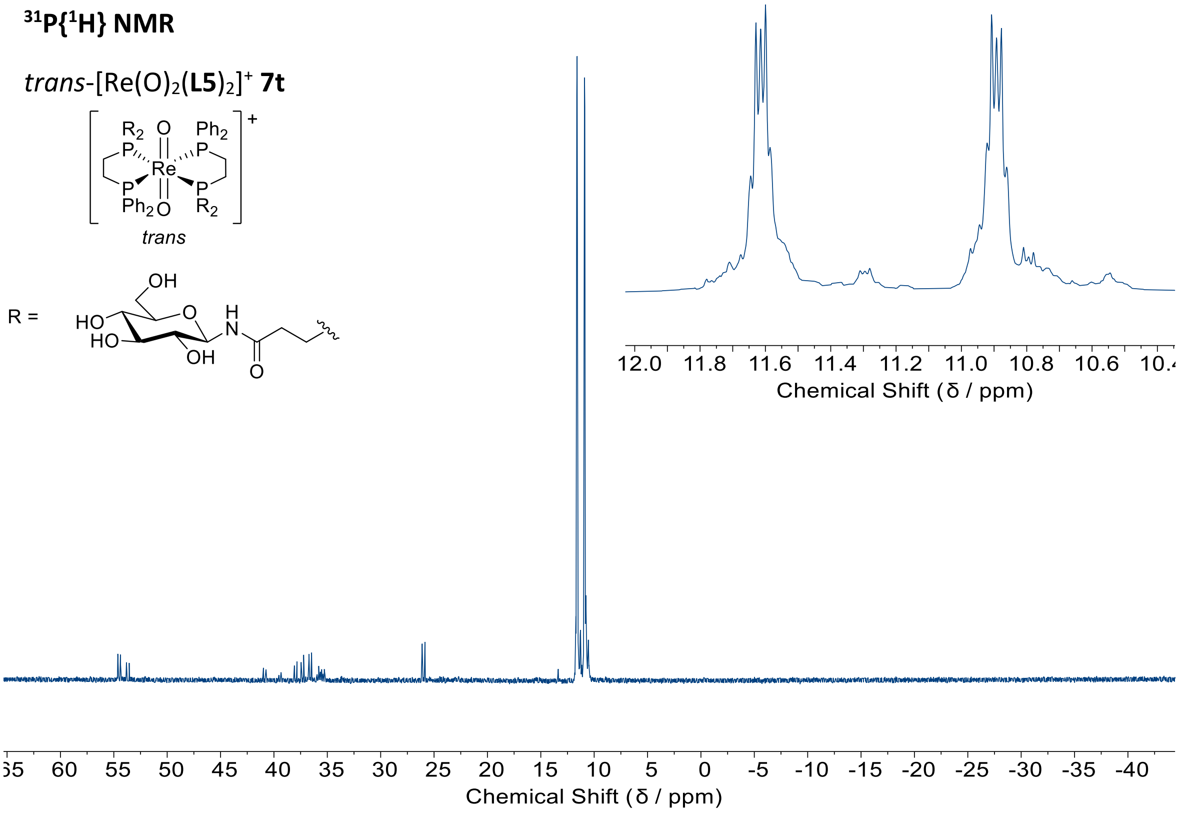

$^{31}P\{^{1}H\}$ NMR

cis-[Re(O)$_2$(**L5**)$_2$]$^+$ **7c**

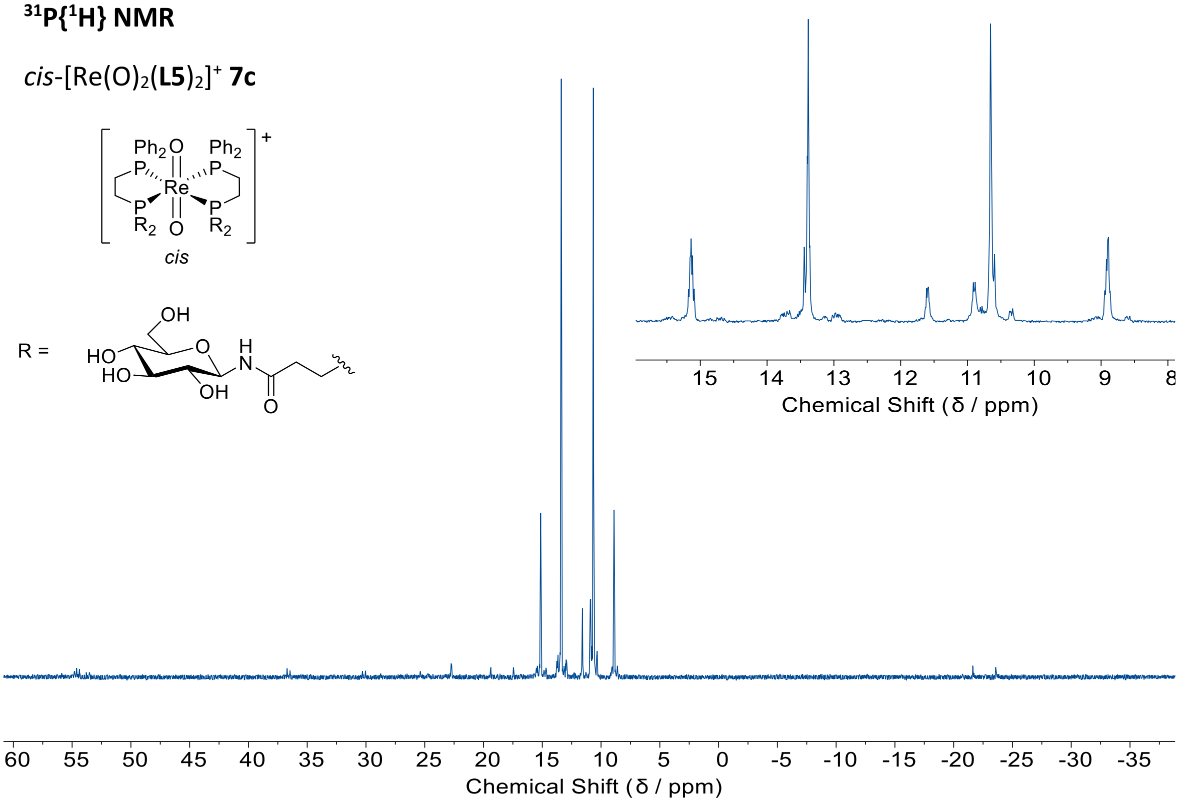

^{1}H NMR

6

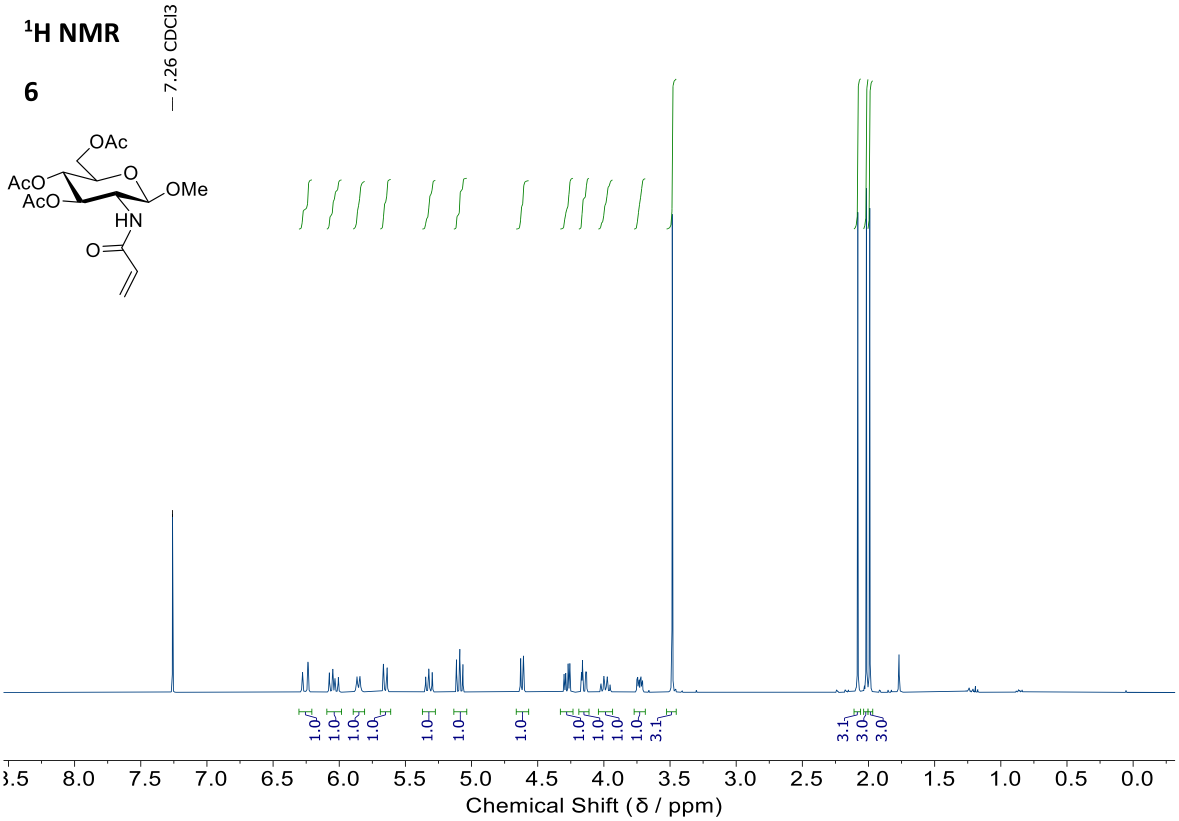

^{13}C{^{1}H} NMR

6

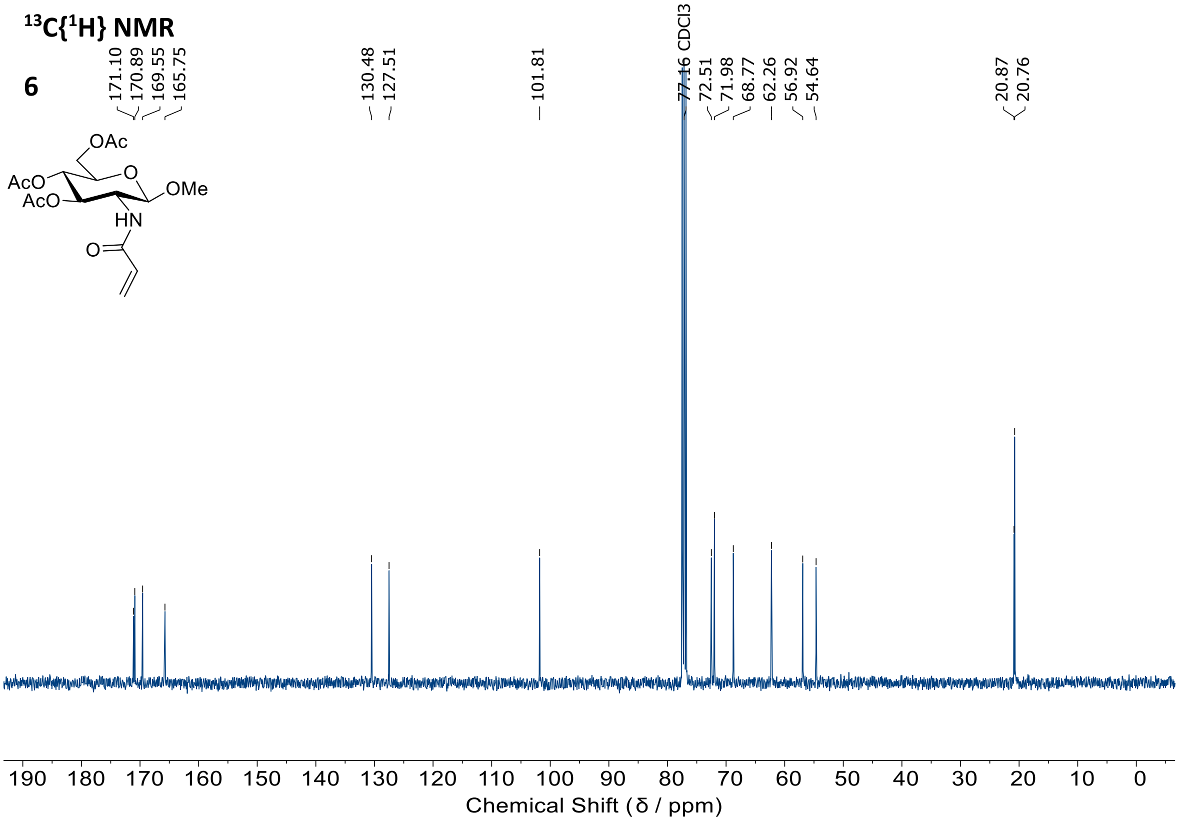

^{1}H NMR

S6

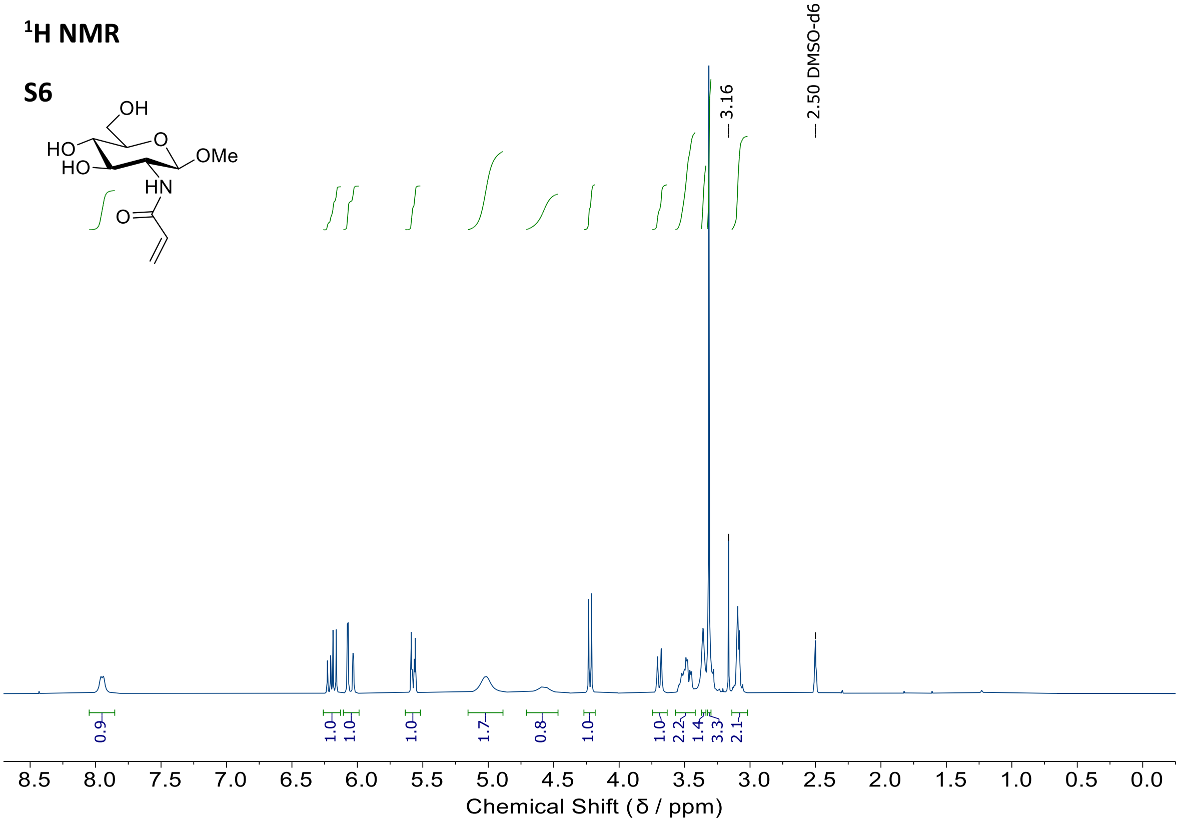

$^{13}C\{^{1}H\}$ NMR

S6

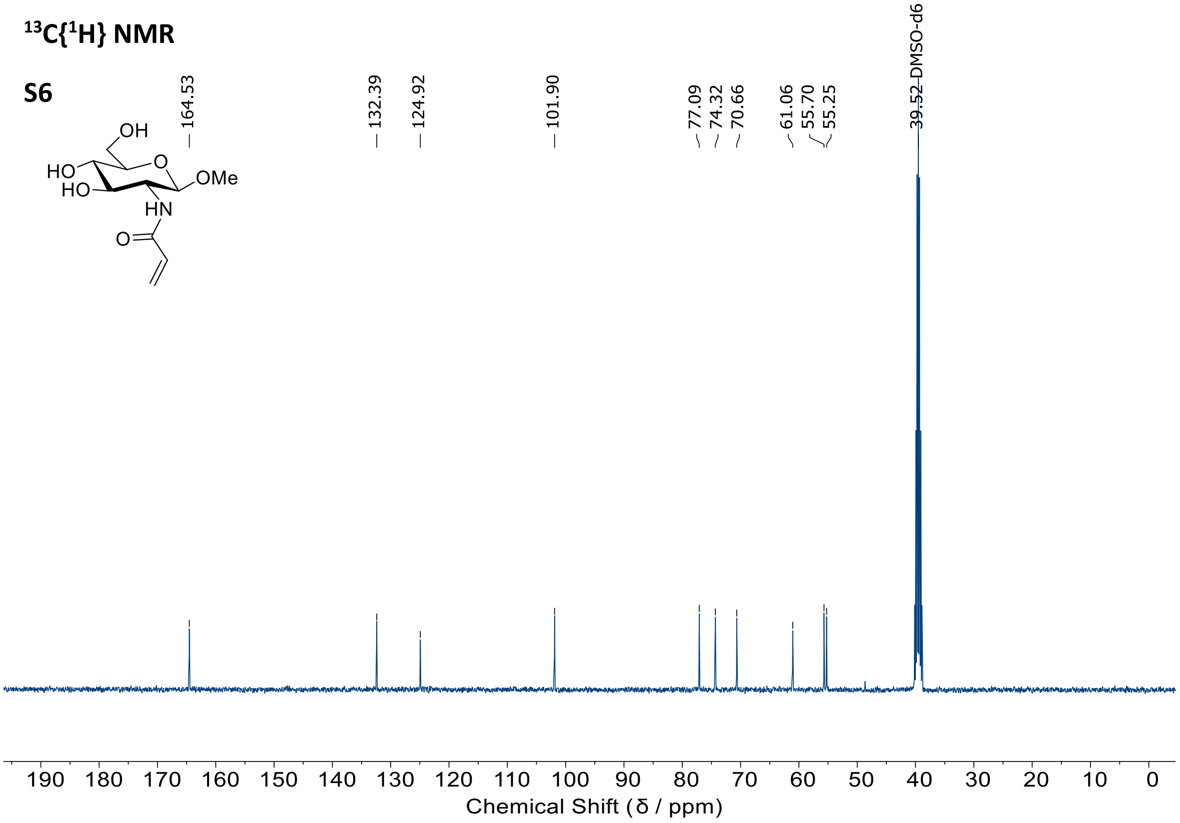

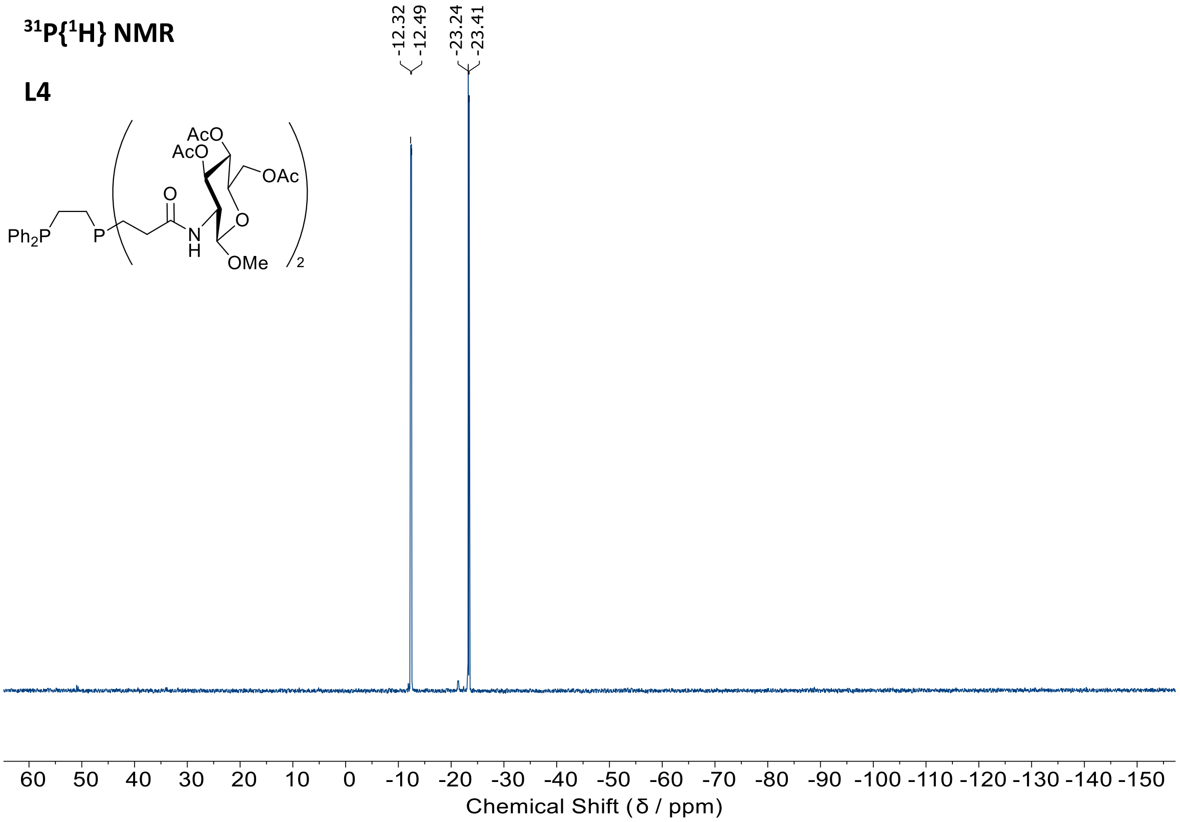

$^{31}P\{^1H\}$ NMR
L4
-12.32
-12.49
-23.24
-23.41
AcO
AcO
OAc
O
O
Ph$_2$P
P
N
H
OMe
2
60 50 40 30 20 10 0 -10 -20 -30 -40 -50 -60 -70 -80 -90 -100 -110 -120 -130 -140 -150
Chemical Shift (δ / ppm)

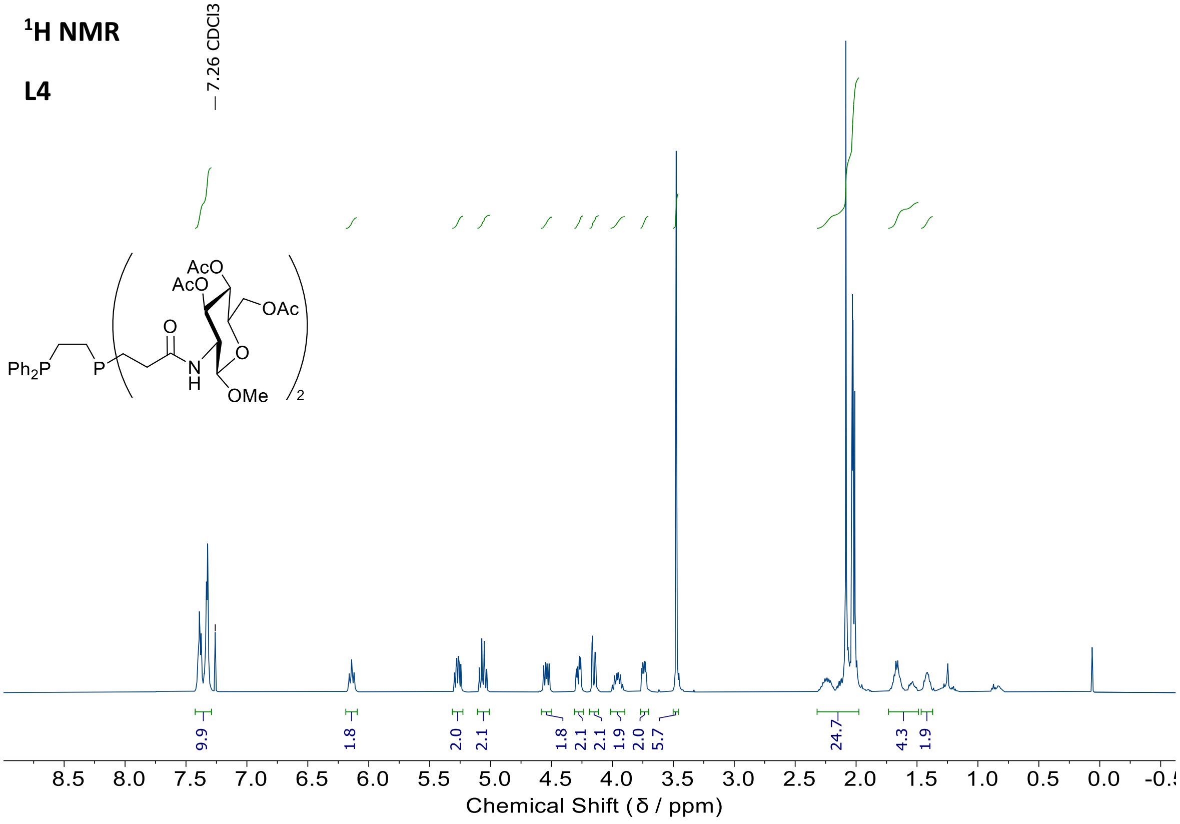

^1H NMR
L4
7.26 CDCl3
AcO
AcO
OAc
O
O
Ph$_2$P
P
N
H
OMe
2
9.9
1.8
2.0
2.1
1.8
2.1
2.1
1.9
2.0
5.7
24.7
4.3
1.9
8.5 8.0 7.5 7.0 6.5 6.0 5.5 5.0 4.5 4.0 3.5 3.0 2.5 2.0 1.5 1.0 0.5 0.0 -0.
Chemical Shift (δ / ppm)

$^{13}C\{^1H\}$ NMR

L4

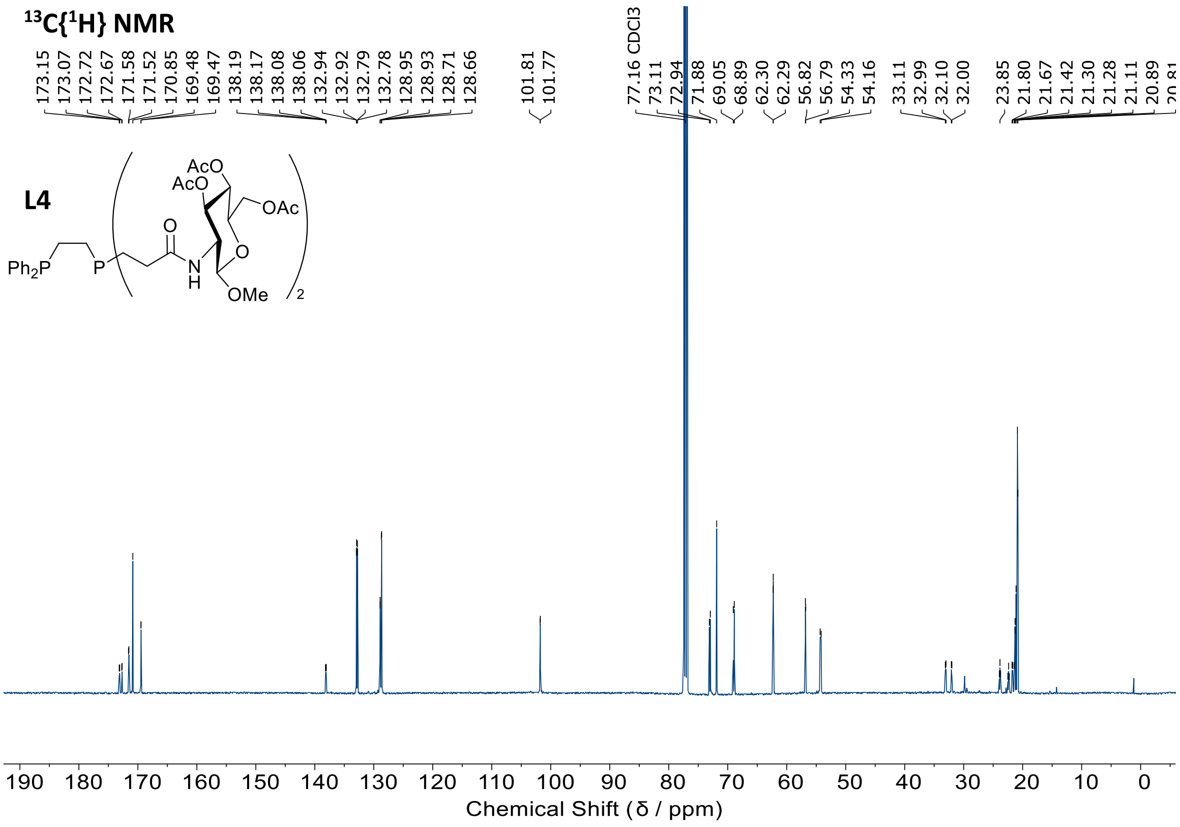

$^{31}P\{^1H\}$ NMR

L6

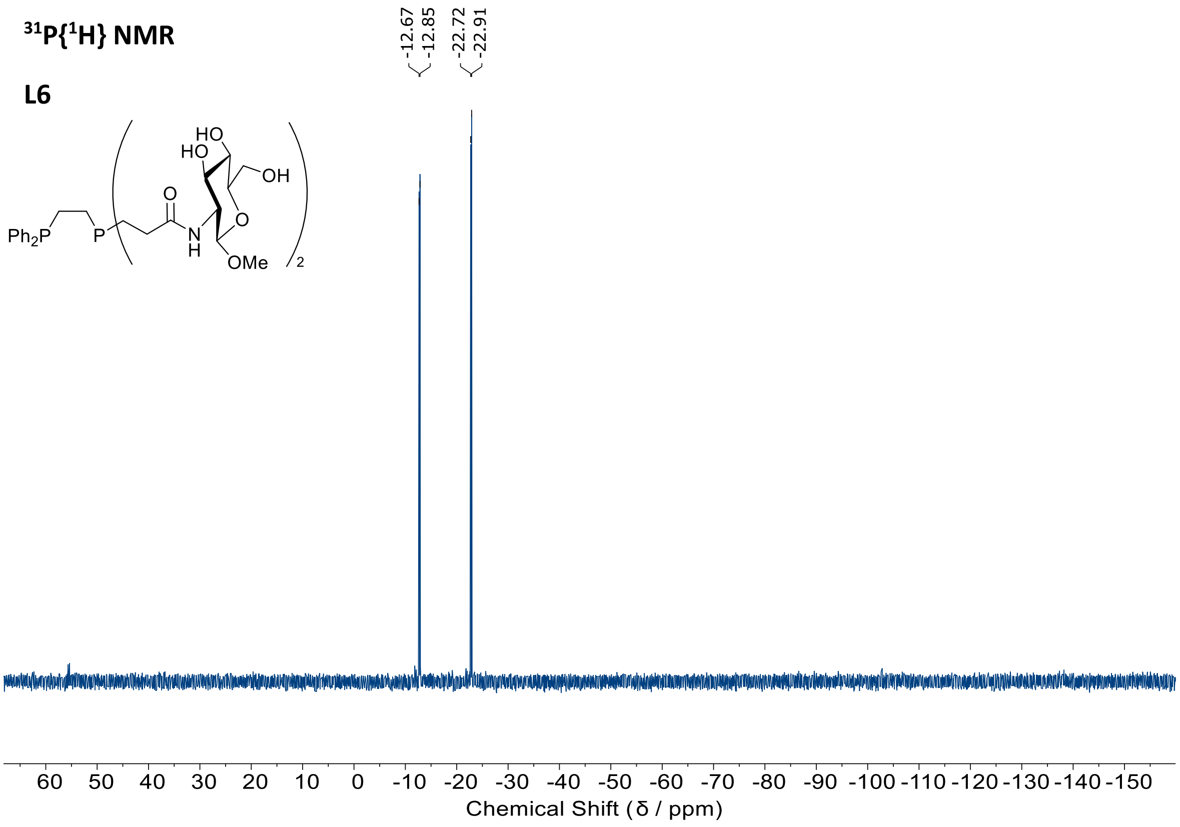

^{1}H NMR

L6

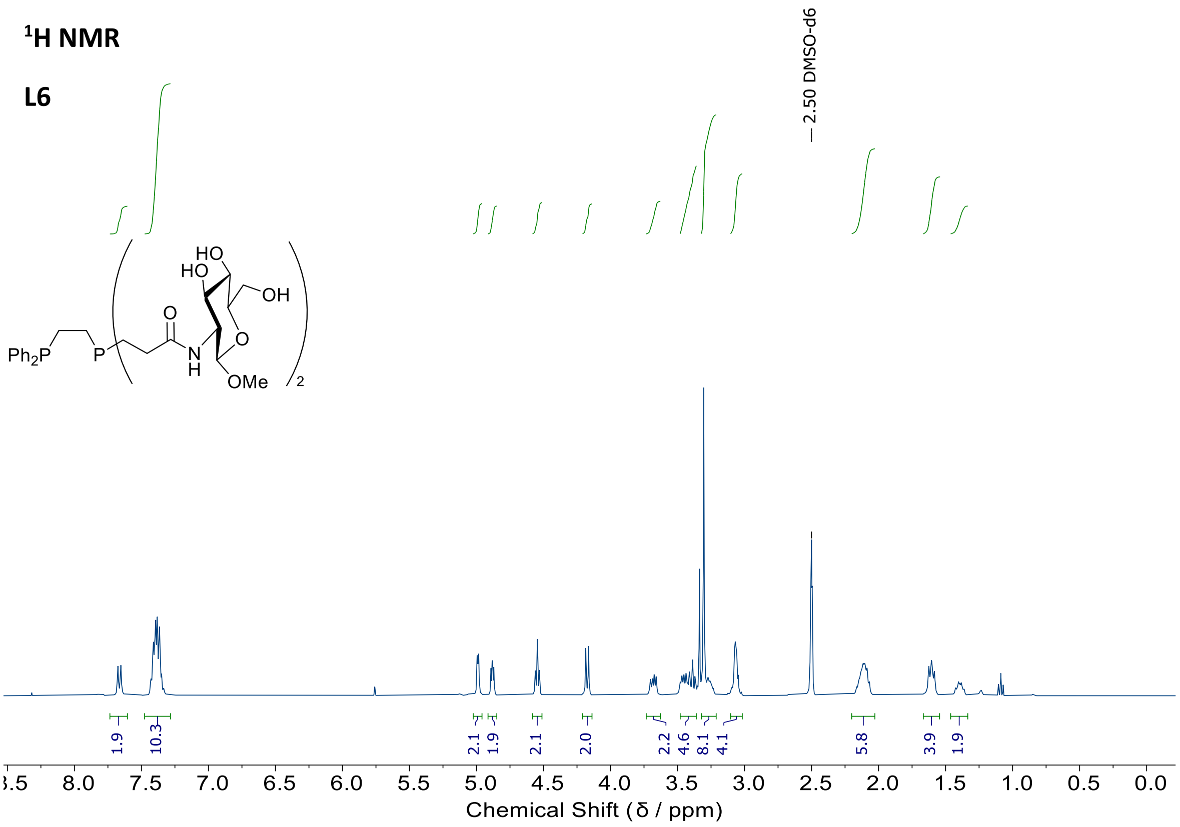

^{13}C{^{1}H} NMR

L6

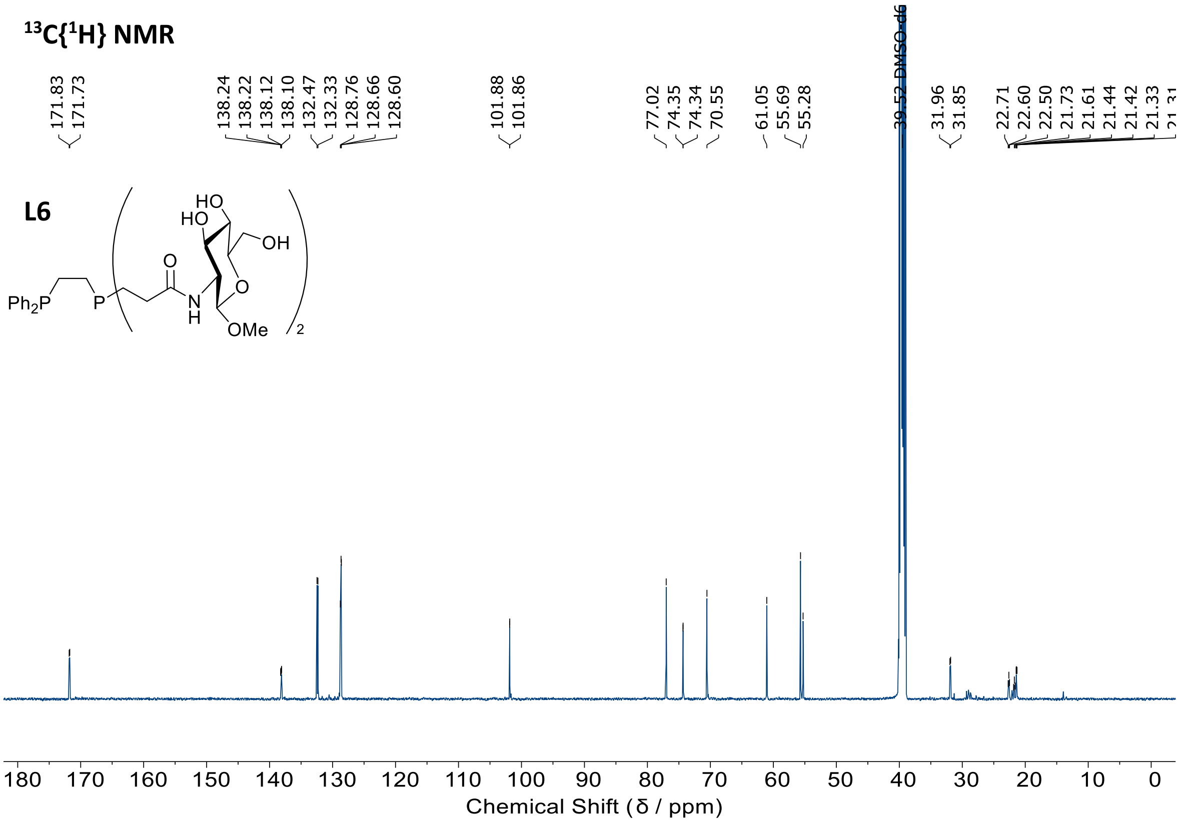

$^{31}P\{^1H\}$ NMR

$[Re(O)_2(\mathbf{L6})_2]I$, **8c** and **8t**

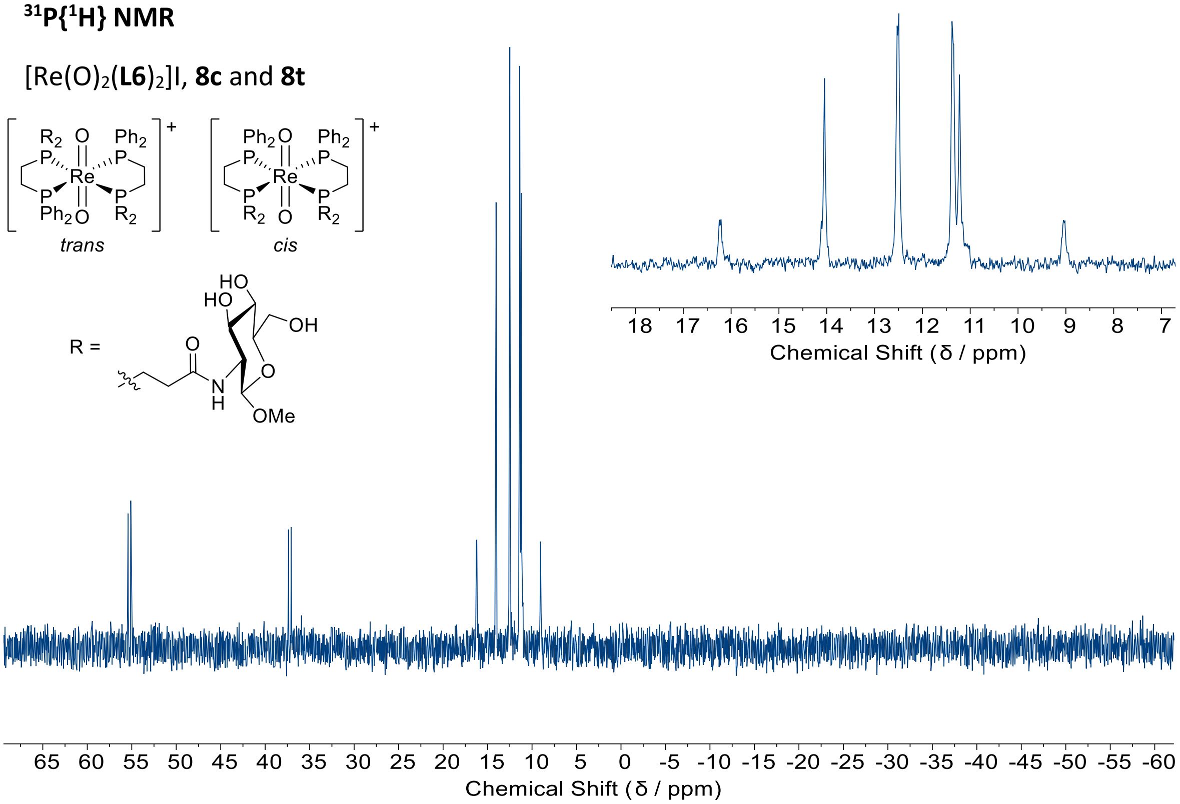

7 REFERENCES

(1) Tang, J.; Ozhegov, E.; Liu, Y.; Wang, D.; Yao, X.; Sun, X.-L. Straightforward Synthesis of N-Glycan Polymers from Free Glycans via Cyanoxyl Free Radical-Mediated Polymerization. *ACS Macro Lett.* **2017**, *6* (2), 107–111.

(2) Wiedner, E. S.; Roberts, J. A. S.; Dougherty, W. G.; Kassel, W. S.; Dubois, D. L.; Bullock, R. M. Synthesis and Electrochemical Studies of Cobalt(III) Monohydride Complexes Containing Pendant Amines. *Inorg. Chem.* **2013**, *52* (17), 9975–9988.

(3) Angelici, R. J. *Inorganic Syntheses: Reagents for Transition Metal Complex and Organometallic Syntheses, Vol. 28*; John Wiley & Sons: USA, 1990.

(4) Chadwick, A. C.; Heckenast, M. A.; Race, J. J.; Pringle, P. G.; Sparkes, H. A. Self-Replication of Chelating Diphosphines via Pt(0)-Catalyzed Hydrophosphination. *Organometallics* **2019**, *38* (19), 3871–3879 and references therein.

(5) Doyle, L. M.; O'Sullivan, S.; Di Salvo, C.; McKinney, M.; McArdle, P.; Murphy, P. V. Stereoselective Epimerizations of Glycosyl Thiols. *Org. Lett.* **2017**, *19* (21), 5802–5805.

(6) Lin, Y. A.; Chalker, J. M.; Davis, B. G. Olefin Cross-Metathesis on Proteins: Investigation of Allylic Chalcogen Effects and Guiding Principles in Metathesis Partner Selection. *J. Am. Chem. Soc.* **2010**,

132 (47), 16805–16811.

(7) Mangunuru, H. P. R.; Yerabolu, J. R.; Liu, D.; Wang, G. Synthesis of a Series of Glucosyl Triazole Derivatives and Their Self-Assembling Properties. *Tetrahedron Lett.* **2015**, *56* (1), 82–85.

(8) Ghirardello, M.; Ledru, H.; Sau, A.; Galan, M. C. Chemo-Selective Rh-Catalysed Hydrogenation of Azides into Amines. *Carbohydr. Res.* **2020**, *489*, 107948.

(9) Wang, Y.; Deng, L.-F.; Zhang, X.; Mou, Z.-D.; Niu, D. A Radical Approach to Making Unnatural Amino Acids: Conversion of C−S Bonds in Cysteine Derivatives into C−C Bonds. *Angew. Chem. Int. Ed.* **2021**, *60* (4), 2155–2159.

(10) Billing, J. F.; Nilsson, U. J. Cyclic Peptides Containing a δ-Sugar Amino Acid - Synthesis and Evaluation as Artificial Receptors. *Tetrahedron* **2005**, *61* (4), 863–874.

(11) Yeung, B. K. S.; Adamski-Werner, S. L.; Bernard, J. B.; Poulenat, G.; Petillo, P. A. The Mild Cleavage of 2-Amino-2-Deoxy-D-Glucoside Methoxycarbonyl Derivatives. *Org. Lett.* **2000**, *2* (20), 3135–3138.

(12) Bruker. *SAINT+ v8.38A Integration Engine, Data Reduction Software*; Bruker Analytical *X*-ray Instruments Inc.: Madison, Wisconsin, USA, 2015.

(13) Bruker. *SADABS 2014/5, Bruker AXS Area Detector Scaling and Absorption Correction*; Bruker Analytical *X*-ray Instruments Inc.: Madison, Wisconsin, USA.

(14) Palatinus, L.; Chapuis, G. SUPERFLIP – a Computer Program for the Solution of Crystal Structures by Charge Flipping in Arbitrary Dimensions. *J. Appl. Crystallogr.* **2007**, *40* (4), 786–790.

(15) Palatinus, L.; Prathapa, S. J.; van Smaalen, S. EDMA: A Computer Program for Topological Analysis of Discrete Electron Densities. *J. Appl. Crystallogr.* **2012**, *45* (3), 575–580.

(16) Sheldrick, G. M. Crystal Structure Refinement with SHELXL. *Acta Crystallogr. C* **2015**, *71* (1), 3–8.

(17) Sheldrick, G. M. A Short History of SHELX. *Acta Crystallogr. A* **2008**, *64*, 112–122.

(18) Dolomanov, O. V.; Bourhis, L. J.; Gildea, R. J.; Howard, J. A. K.; Puschmann, H. OLEX2: A Complete Structure Solution, Refinement and Analysis Program. *J. Appl. Crystallogr.* **2009**, *42* (2), 339–341.
